# Supplementary material for: Systematic review of infant and young child feeding practices in conflict areas: what the evidence advocates
Source: BMJ Open. 2020 Sep 13;10(9):e036757. doi: 10.1136/bmjopen-2020-036757 (PMC7488834; doi:10.1136/bmjopen-2020-036757)
Supplement: Supplementary data [file bmjopen-2020-036757supp001.pdf]

Appendix

Table 1: operational guidelines for IYCF in conflict

Components of breastfeeding	Operational Guidelines
**I.** Protecting, promoting and supporting breastfeeding	1. Infants should be exclusively breast fed for the first 6 months of life, followed by appropriate complementary feeding along with continued breastfeeding for two[1-14]<br>2. Relief agencies should create internationally-coordinated breastfeeding policies with practical guidelines explicitly stated for field workers and decision[2, 7, 10, 12, 13, 15, 16]<br>3. National policies and technical guidelines on IYCF- E should be explicitly stated and communicated to relevant staff in order to increase preparedness if an emergency strikes. This is particularly important for high-risk countries[2, 12, 13, 17, 18]<br>4. Funding should be provided for programs to support breastfeeding[2, 13, 19]<br>5. It should be ensured that optimal breastfeeding practices and maternal care is promoted according to 'UNICEF/WHO Baby Friendly Hospital Initiative's Ten Steps to Successful Breastfeeding'[2, 10, 20]<br>6. Ensure that relief workers, healthcare staff, technical and non-technical personnel are trained in appropriate infant and young child feeding practices using available training material and key information on IYCF integrated into routine assessment[2, 7, 10, 12-16, 20-23]<br>7. Appropriate and timely support and trainings for breastfeeding and young child feeding should be integrated at all levels of healthcare[2, 16, 21, 23]<br>8. Efforts should be made to raise awareness supporting the superiority of breastfeeding as a life-saving intervention to health personnel, relief staff, NGOs, stakeholders and the general public focusing primarily on pregnant and breastfeeding women[2, 10, 12, 20, 22, 24, 25]<br>9. In conflict and refugee settings, traditional birth attendants (TBAs) are more accessible to mothers than nurses and midwives, therefore TBAs should be trained regarding appropriate breastfeeding practices[17, 20, 24]<br>10. Pregnant women and breastfeeding mothers should be informed using clear language about maternal health, how to properly breastfeed, advantages and maintenance of breastfeeding, negative effects of bottle-feeding and the difficulty to reverse decision not to breastfeed[2, 6, 7, 10, 14, 17, 20, 22, 24, 25]<br>11. Encourage mothers to initiate breastfeeding within the first hour of birth, and to exclusively breastfeed for the first six months of life (do not give them extra water, juices, tea or food) unless medically indicated otherwise. Thereafter, solid foods should be introduced, but breastfeeding should continue for at least a year or two[2, 6, 7, 10, 11, 14, 15, 17, 26]<br>12. Educate mothers to not stop breastfeeding in emergency situations and spread awareness that exclusive breastfeeding provides the best nutrition to babies. Moreover, breastmilk contains ingredients that protects babies from infection, so it's particularly useful in emergency situations[2, 6, 10, 13, 15, 17]<br>13. Encourage mothers to breastfeed on demand[2, 7, 14, 17]<br>14. Practice rooming-in of infants and their mothers (allow mothers and infants to remain together 24 hours a day) to support breastfeeding practice [2, 7]<br>15. Colostrum should be given to the baby to improve its physical growth and feeding baby should not be interrupted in between. The baby lets go off the breast when he/she is done[10, 17, 26]<br>16. Discourage the use of artificial nipples or pacifiers[2, 7, 10, 24]

	17. Educate mothers on breastfeeding and the procedure to maintain lactation even when the mother-infant pair is temporarily separated [2, 7, 10, 24]<br>18. Ensure that mothers are facilitated by the provision of counseling and other forms of assistance for the purpose of re-lactation or difficulty in breastfeeding[2, 6, 7, 10, 11, 14, 21, 23, 26, 27]<br>19. Mothers should also be made aware that breast milk supply is not reduced by stress, though the release of milk could be affected[6, 8, 10, 14, 18]<br>20. Encourage mothers to practice skin to skin contact as this aids in reducing stress (cortisol) levels and helps the flow of mother's milk. This can be practiced using slings and wraps. Moreover, correct positioning of baby during breastfeeding is important as effective suckling also triggers breast milk production[6, 10, 11, 14, 15, 17, 24, 26]<br>21. Malnourished mothers can produce enough milk to breastfeed, however, she should be treated for malnourishment[8, 10, 14, 15, 17, 26]<br>22. Relief workers should provide nutritional support by giving a general ration to pregnant and lactating women. If full general ration is not possible, then food and micronutrient supplements should be sufficiently provided. This is because an optimally fed mother will be able to optimally feed her infant. Give adequate food to the malnourished mother to prevent depletion of her own nutrients and closely monitor the weight and urine production of the infant[6, 10, 11, 15, 17, 18, 21, 23, 24, 26]<br>23. In emergency settings, extra breastfeeding support should be provided by encouraging Baby Friendly Spaces, which provide a platform to support mothers/caregivers and promote appropriate infant and young children feeding practices, privacy and safety[2, 11, 12, 15, 18, 21-24, 26]<br>24. For refugees and displaced populations, establish rest areas for pregnant women/caregivers/mothers and children in transit. These should be secluded, private and culturally appropriate areas that assist women and children to relax and nurse[2, 6, 11, 15, 18, 21, 23, 24]<br>25. Establish a program to encourage mother to mother/ women to women support[2, 6, 7, 20, 21, 23, 24]<br>26. Establish registration of newborn infants, i.e. within two weeks of delivery, to ensure timely additional rations and breastfeeding support for lactating and breastfeeding mothers. Nutrition workers should help mothers to establish exclusive breastfeeding[2, 6, 12, 16, 18, 23, 24]<br>27. Vulnerable groups, especially newly arriving mothers and infants with severe feeding problems should be identified and referred for immediate assistance[2, 6, 12, 15, 16, 18, 21, 23]<br>28. Create referral and follow-up systems for mothers/caregivers and their infants[2, 3, 6, 7]<br>29. Avoid giving estrogen containing contraceptive pills to mothers because they decrease breastmilk production[24]<br>30. Encourage mothers to build their confidence which leads to production of oxytocin to improve blood flow[26]<br>31. Continue to breastfeed sick children or when they are less hungry. In case they are not hungry then put them to breast more repeatedly to ensure that they take enough breastmilk[6, 10, 11, 14, 17, 26]<br>32. When natural breastfeeding is not possible, available alternatives should be evaluated before an appropriate choice is made. This may include evaluating between: wet-nursing, the use of milk bank, home-modified milk and the use of locally purchased commercial infant formula or generic unbranded infant formula[10, 12, 15, 23, 24, 28]<br>33. Access to infant formula should be based on the guidelines set by the WHO International Code of Marketing of Breast Milk Substitutes, 1981[10, 25]<br>34. Support should be provided for artificial feeding and this should be distinct from the support being provided for breastfeeding[1, 2, 4, 6, 10, 12, 19, 23]<br>35. Lactating women can take most medicines (including antibiotics) and can be immunized as well, as recommended for adults and adolescents to protect against infectious diseases (measles, mumps, rubella, tetanus, diphtheria, pertussis, influenza, Streptococcus pneumoniae, Neisseria meningitidis, hepatitis A, hepatitis B, varicella, and inactivated polio)[15]<br>36. In case of radiation exposure

		a. Women who were exposed to radiation should be advised to temporarily stop breastfeeding unless there is no other source of feeding available for the infant. These mothers should pump and discard their milk until the infant can resume breastfeeding<br>b. In case of interruption of breastfeeding, preferred source of infant feed is human milk that was pumped and stored prior to the radiation exposure or ready-to-feed infant formula. Mothers should use powdered or concentrated formula only if they are sure that water used to reconstitute is free from radiation<br>c. Mothers can resume breastfeeding when advised by local health officials or when they have been evacuated from the radiation affected area<br>d. Mothers do not need to stop breastfeeding if both mother and infant have been given appropriate doses of potassium iodide at the right time, according to the advice of local health officials[15]
**II.**	Protecting non-breastfed infants and minimizing the risks of artificial feeding	1. Breast milk substitute (BMS) is not recommended for children <6 months of age[3, 4, 6, 7, 9, 10, 19, 21, 25, 28]<br>2. Procurement, management and distribution of breastmilk substitutes, bottles and teats, and commercial complementary foods should be controlled during emergencies and should comply with the 'no bottle' policy and International Code and WHA guidelines and violations should be reported[1-3, 10, 13, 16, 19, 21-23]<br>3. Procure BMS after conducting need assessment of artificial feeding at population level assessment. It may also include nutrition survey, household and community survey. This is recommended to be done in close collaboration with government bodies and by developing Program Cooperation Agreements (PCAs) with local bodies for task implementation[28]<br>4. On distribution of BMS, it should be ensured that workforce involved should have enough capacity for assessment, counselling, supply chain management and in providing support to families on WASH and IYCF practices[28]<br>5. Donations of free or subsidized breast-milk substitutes, bottles and teats and commercial baby foods at emergency sites should be refused and advocated against as this could put infants lives at risk and undermine breastfeeding practices[2-4, 6, 8, 11-13, 16, 21-23, 28]<br>6. The distribution, use and quantity of breast milk substitutes should be controlled by collecting unsolicited donations from all ports of entry and the recipient agencies, and should be stored and managed centrally by a single designated agency[1, 2, 4, 6, 10, 12, 13, 16, 19, 21, 23, 28]<br>7. BMS should only be distributed to the infants who really need it and have no viable breastmilk options, based on established criteria (where distribution can be targeted, the supply chain is secure, and the conditions for safe preparation and use can be met). This should be determined by a qualified health or nutrition worker trained in breastfeeding and infant feeding issues[1-4, 6, 7, 9-12, 14-16, 19, 21-23, 27]<br>8. Breast-milk substitutes, milk products, bottles and teats should never be part of a general or blanket distribution and their use should be avoided, especially in case of emergency situations or in situations where hygienic conditions can't be ensured and their use should be discouraged. Bottles should be exchanged for cups instead as it's easier to keep them clean[1-4, 6, 7, 10-12, 16, 18, 19, 21, 23, 24, 26-28]<br>9. Promotion of breast-milk substitutes at the point of distribution should be strictly discouraged. There should be no advertisement or display of products or items with milk company logos and BMS supplies should not be used as a sales inducement and there should be no provision of single tins/samples of BMS or gifts to mothers. Moreover, no incentives should be offered by manufacturers to health workers to promote BMS products[6, 9, 10, 21-23, 25, 28]<br>10. In case of crisis, WHO developmental partners and/or the designated nutrition co-ordinating agency should train as well as support training of staff and mothers on the proper and safe use of infant formula[2, 4, 10, 12, 16, 23]<br>11. When the use of infant formula is indicated, before its distribution, mothers and caregivers responsible for feeding should be educated on the specific care needed by a non-breast fed infant, and given practical training and one-

	on one demonstrations by a skilled health worker on safe and hygienic preparation and administration of infant formula[1, 2, 4, 6, 7, 10, 12, 19, 21, 23]<br>12. There should be regular follow-up visits, to caregivers of targeted infants receiving BMS comprising of regular infant health and growth monitoring and distribution of BMS should be frequent and regular (at least bimonthly), with minimum delays[1, 3, 7, 10, 12, 19, 21]<br>13. It should also be ensured that there is availability of fuel, safe water and sanitation and equipment for safe preparation of BMS in the house, prior to distribution of BMS and implementing a household based program[2-4, 6, 7, 9-11, 14, 15, 21, 23, 25, 26, 28]<br>14. For infants determined to be in need of infant formula, the adequate and continued provision of infant formula must continue for as long as they require it, even if it needs to be purchased. IYCF-E programs that include an infant formula provision component should plan to provide it for at least 6 months, or if there is no availability of adequate complementary food, then 12 months[3, 4, 6, 7, 9-12, 14, 19, 22]<br>15. Non-breastfed infants over 6 months of age, provided with 6 months of BMS, may require 2-4 weeks of buffer supply to offer transition to non BMS feeding[3, 10]<br>16. An improvement should be brought about in health services to manage the adverse effects of artificial feeding, especially the increase in incidence of severe diarrhea and respiratory infections in infants[2, 10]<br>17. Generic, unbranded formula is recommended for infants who require infant formula, followed by locally purchased and relabeled infant formula (to be in compliance with The International Code). Home modified milk should only be used temporarily as the last resort for infants less than 6 months old. The type of infant formula should have a shelf life of at least 6 months and be appropriate for the infant, including their age[4, 6, 7, 9, 10, 15, 19, 21, 23]<br>18. Labels must adhere to the specific labelling requirements of The International Code and should be in the language of the local population. The BMS packaging should have clear instructions, with pictures, on how to use it, along with clearly specifying the superiority of breast milk[4, 6, 7, 9, 19, 21, 25, 28]<br>19. The labels should include a sign of "Important Notice" stating importance of breastfeeding and a statement stating, "The product should be used only on the advice of a health worker as to the need for its use and the proper method of use." They instructions should also state about its safe preparation and health hazard on inappropriate use, and a warning against the health hazards on inappropriate preparation of infant formula[28]<br>20. Supply department should assure that BMS manufactured should follow Codex Alimentarius standards. UNICEF has not included BMS in it supply catalogue as a non-standard product[28]<br>21. For infants under 6 months of age, the only suitable BMS is infant formula. However, infants over the age of 6 months do not need infant formula but can use other sources of milk (pasteurized full-cream animal milk (cow, goat, sheep), Ultra High Temperature (UHT) milk, fermented milk or yogurt) as these are easier to find and are less dangerous than powdered milk. Condensed milk should not be used for infant feeding[4, 6, 10]<br>22. Liquid milk, if being used, should be consumed within a few hours of opening. Baby juices and teas should be avoided as they are low in nutrition and high in sugar. For infants over 6 months of age, infant formula can be mixed into the child's food instead of giving it to drink[6]<br>23. Milk products should only be received and distributed in a dry form and even dried milk products should be distributed only when pre-mixed with a milled staple food and should not be distributed as a single commodity. However, dried skim milk is not an appropriate BMS for infant, and even for older children it must be fortified with vitamin A and not given on its own[4, 7, 9, 19, 23]<br>24. It should be ensured that there are no spill-over risks of infant formula provision. Measures should be taken to reduce spill-over by ensuring that feeding BMS to a minority of children doesn't undermine breastfeeding practices of the majority. Advertising of infant formula should be stopped, and the provision of infant formula should be discrete, monetary support should be

	provided to breastfeeding mothers, and a separate space dedicated for breastfeeding support and counseling[1, 2, 4, 6, 10, 12, 19, 23]<br>25. When circumstances are not favorable for BMS distribution (safe preparation and use of infant formula can't be ensured), an on-site supplementary "wet" feeding program should be conducted in closed spaces under supervision[4, 7, 21, 23]<br>26. Infant formula should not be excluded from the commodities that a mother can access via general cash/voucher schemes. However, it should be accompanied by interventions such as providing essential information on breastfeeding and on how to reduce the risks of formula feeding[6]<br>27. Breast milk donations should be authorized and be regulated by the Ministry of Health. Until the arrival of the beneficiary, cold chain should be maintained for donated frozen breast milk[2, 26, 28]<br>28. The psychosocial impact of discontinued breastfeeding on children whose mothers have died or are no longer able to breastfeed should also be considered and support services should be set up to address this[12, 16]<br>29. One guideline mentioned that it should be known and taken care of that transitioning out from an emergency IYCF-E program with an infant formula provision component requires a solid, long-term exit strategy with ties to both facility and community-based structures[12]
**III.** Protecting, promoting and supporting appropriate, safe and timely complementary feeding	1. To promote growth and development of infants, encourage appropriate, timely and safe complementary feeding for infants (aged > 6 months) and young children (aged between 12 to < 24 months), along with continued breastfeeding[1-7, 9, 11, 12, 14, 18, 23]<br>2. Children over the age of 6 months of age should be given complementary foods (solid, semi-solid and soft foods) that are easy to eat and digest and nutritionally complement breast milk[1, 2, 6, 18]<br>3. Conduct discussion in groups on complementary feeding and weaning[26]<br>4. Promote the growth of locally produced food and encourage mothers/caregivers to use these locally-produced, inexpensive foods for complementary feeding. These include basic food commodities, such as: fruits, vegetables, rice, beans and lentils[9, 12, 18, 23]<br>5. Increase frequency and variety of complementary food with the growing age of child to meet his/her nutritional needs[26]<br>6. In emergencies, locally produced foods, micro-nutrient fortified blended foods, ferrous sulphate iron solution (iron drops), Ready-to-Use Supplementary Foods (RUSFs) or Lipid based Nutrient Supplements (LNS) can be used for complementary feeding depending on the nutritional situation[12, 18, 23, 28]<br>7. Special attention must be given to ensure that the food ration distributed for old infants and young children have an adequate nutritional value (food should be fortified with vitamins and minerals[4, 6, 16, 20, 23]<br>8. Establish services to extend nutritional support to vulnerable groups, such as: orphans and unaccompanied infants and young children[2, 6, 12, 15, 16, 21, 23]<br>9. Promote hygienic preparation of complementary foods by providing mothers/caregivers necessary information and support[6, 23]<br>10. In emergencies, special consideration should be made to mitigate the obstacles faced by mothers/caregivers in the preparation (cook, mash, etc.) of age-appropriate complementary foods. Measures needed to counter these obstacles should be incorporated in the program design[6, 12, 18]<br>11. IYCF-E programs relating to complementary feeding should include:<br>a. Group sensitization and education<br>b. Develop local food recipes focusing on nutritional value, affordability and food dietary diversity<br>c. Provide cooking demonstrations, and<br>d. Support mothers/caregivers to prepare complementary foods via initiatives such as fresh food vouchers[12]<br>12. Dependence upon commercially-produced complementary foods should be avoided[9, 23]<br>13. Mothers should be assured that in the absence of safe complementary foods, breastmilk is a significant source of nutrition for infants the first year of life and beyond[15]

	14. Donations of complementary foods, baby teas, or juices should be refused. If there are donated supplies, they should be directed to the designated coordinating agency on nutrition/health to be managed appropriately[6]<br>15. Promotion of a varied diet, dairy products in particular, to ensure that energy, protein, mineral and vitamin requirements are met. Moreover, when preparing food for infants such as porridges, mashed potatoes, etc., milk and infant formula can be added to it to increase the nutrient content[4, 6, 20, 26]<br>16. Pastoral communities mainly use milk and milk products, which contains a significant amount of nutrition for children over six months. As a part of complementary feeding, milk (such as animal milk) and milk products (such as yoghurt) can be provided to infants over 6 months of age. In such cases, distribution of milk products can only be conducted in controlled environments under strict supervision, such as on-the-spot feeding[3]<br>17. Good nutrient sources for infants are animal source foods, such as yogurt and cheese whereas products containing only fruits/vegetables are less energy and nutrient dense. Baby teas and juices should not be given to infants as they do not have much nutrient value[6]<br>18. Complementary food products should be labelled in the language of the targeted population and be acceptable to them, according to their culture. The products should also have information on how to prepare the food[6]<br>19. Moreover, these products should not have images of bottle feeding on them or recommend it in any way[6]<br>20. If needed, commercial 'baby' foods should be purchased and distributed to mothers or products recommended to mothers, for infants over 6 months of age, that have the most nutritional value[6]<br>21. It is recommended that children over the age of 12 months eat the same foods as older children[6]
**IV.** Caring for care-givers	1. Efforts should be made to increase caregivers' coping capacity because the number of caregivers is often reduced during emergencies and stress levels increase, so psychological support and encouragement plays an important role in enhancing optimal IYCF-E practices[1, 2, 15, 17, 18]<br>2. Treat the mother during illness, keep the infant close to the mother and do not stop breastfeeding. The mother should be educated on the appropriate method of milk expression as she can maintain breastmilk flow through milk expression[17, 24]<br>3. Breastfeeding mothers require identification, protection and active support. Conflict situations and displacement can negatively affect maternal confidence and breastfeeding practices. Breastfeeding mothers tend to stop or reduce breastfeeding in such situations and thus a lot of support and counselling should be provided to newly arriving refugee mothers, caregivers and infants with special needs (orphans and unaccompanied children)[2, 5, 6, 12, 17, 18, 21, 23]<br>4. Provide restorative care to mothers in case of trauma, stress, sexual violence, grief or infant rejection[24]<br>5. Adequately trained and skilled staff should support mothers with difficulties in breastfeeding their infants and provide private safe spaces for mothers to breastfeed and to connect with other mothers. Access to cleaning facilities should also be provided to mothers to wash feeding utensils, especially to mothers who are formula feeding. Prioritize mothers of infants and young children for basic screening of childhood illnesses, access to registration and basic services, shelter and non-food items[2, 6, 10, 15, 18]<br>6. It should be ensured that mothers/caregivers of artificially fed infants are given targeted support and receive counselling as well as kits for preparing BMS safely, including soap, fuel, water purification tablets[2, 18]
**V.** Protecting Children	1. Artificially-fed infants need more protection and support than breast-fed children[6, 10]<br>2. Infants are at higher risk of malnutrition and illness like diarrhea and chest infections if they are being fed infant formula. In emergency conditions, this could be fatal[2, 6, 10, 11]<br>3. In case the infant is ill (e.g. if the infant has diarrhea), continue feeding the infant since breastmilk contains water to replace losses through diarrhea and important minerals and vitamins to help prevent dehydration. It also contains

	proteins to help strengthen the immune system of the baby. Give smaller amounts of breastfeed and more frequently if the infant's appetite is reduced. However, in serious or prolonged cases of diarrhea, rehydration therapy may be required[6, 10, 11, 15, 26]<br>4. Newborn infants are the most vulnerable group which should be targeted and ensured that they are exclusively breast fed and artificial feeding is prevented[2, 6, 12, 16, 21, 23]<br>5. Support breastfeeding as the best way to safeguard infants against cholera. Infants with cholera should continue breastfeeding as soon as they are able to suckle, and mothers with cholera should re-initiate breastfeeding as soon as they are stable[12]<br>6. Efforts should be made to protect cholera-free breast-fed babies from cross-contamination[12]<br>7. IYCF managers, field staff and registration staff should collaborate and ensure screening of infants at registration to identify and refer infants at risk or those who are highly vulnerable (i.e. orphans and unaccompanied infants and young children, non-breastfed infants <6 months, ill infants or malnourished infants) and establish services to give these vulnerable infants nutritional support[2, 6, 12, 16, 18, 21, 23]<br>8. Prioritize support service:<br>a. For re lactation: Prioritize non-breastfed infants 0 - <6 months or of 0 - <2 months whose mother/wet nurse is willing to re lactate<br>b. Re-establish exclusive breastfeeding: Always prioritize infants aged 0 - <2 months (including newborns). Infants 0 - <4 months are a priority, but it's an ideal situation for infants 0 - <6 months<br>c. BMS provision and associated support services: prioritize not breastfed infants who are <6 months[3]<br>9. The psychosocial impact of discontinued breastfeeding on children whose mothers have died or are no longer able to breastfeed should also be considered and support services should be set up to address this[12, 16]<br>10. Consideration should also be given to special needs of artificially fed children (0-23 months) and PLW (i.e. insecticide treated mosquito nets; hygiene items including soap and washing containers; cooking and eating items; breastfeeding shawls; blankets and infant/young child clothing and shoes with thermal needs considered; potties, diapers), with a particular preference for items that can be locally sourced[2]
**VI.** Malnutrition	1. Ensure availability of endorsed nutritional strategies in policies and guidelines and establish cross-sectoral IYCG working group to discuss challenges, needs and success[2]<br>2. IYCF teams should coordinate and generate a treatment plan where mothers should be admitted at stabilization centers along with her children where they would be provided with nutritional support and counselling to improve lactation[2]<br>3. Select an IYCF specialist from each nutrition post or community group[2]<br>4. Standardize IYCF messages for caregivers and PLWs with children of 0-23 months and incorporate IYCF indicators in the monitoring system[2]<br>5. Develop standard procedure for health workers for identification and referral of malnourished children[2]<br>6. Promote IYCF practices (i.e. early initiation, exclusive breastfeeding and complementary feeding)[2]<br>7. Timely introduction of complementary food in infants of 6-9 months[2]<br>8. Micronutrients should be distributed to all PLWs in form of powders and tablets[2]<br>9. Malnutrition treatment and prevention programs should incorporate and prioritize infant and young child feeding in their agenda[2, 12]<br>10. Efforts should be made to investigate the underlying cause of malnutrition and measures should be taken to correct it[1, 2, 10]<br>11. Monitor nutritional status of mothers, infants and young children with the purpose of identifying, assessing, preventing and treating malnourished children[1, 2, 10]<br>12. Strong referral mechanisms should be put in place for acute malnutrition to cater to worsening nutritional status of infants[2, 12]

	13. Admit malnourished children along with their mothers to a nutritional rehabilitation program[12, 21, 24]<br>14. There should be community-based management of acute malnutrition, if conditions are favorable. In disasters, supplementary feeding should be the primary strategy for prevention and treatment of moderate acute malnutrition (MAM). Depending on vulnerable population groups and malnutrition level/risk of an increase in acute malnutrition, supplementary feeding can be blanket or targeted[2]<br>15. Pre-formulated therapeutic milk products or dried skimmed milk (DSM) may be used to treat cases of severe acute malnutrition (SAM). However, attention must be given to ensure that supplementary food aid commodities are carefully regulated and distributed to only target vulnerable groups[4, 9]<br>16. Therapeutic milks are not appropriate for BMS use. Therapeutic milk like F75 and F100 should be used for the treatment of children with severe acute malnutrition (SAM). It can be given to infants less than 6 months. SAM children require appropriate treatment at right time with immediate referral[28]<br>17. Cash/voucher programmes (conditional) could be started that promote good nutrition outcomes i.e. preventing malnutrition. Unconditional cash programs should be promoted in case of food security and livelihood. But in this case optimal IYCF practices should be considered through an expert. Women should be educated and sensitized for appropriate use on food and nonfood items for children of 0-23 months[2]<br>18. Community support networks should be educated on the prevention and treatment of acute malnutrition[2]
**VII.** The acute phase of emergencies (prevention through interventions)	1. In case of an emergency, interventions should start immediately to minimize the emergency's negative impact on feeding practices and every agency should develop a policy on infant feeding in emergencies, focusing on supporting caregivers and nutritional needs children[1, 2, 4, 23]<br>2. An appropriate agency should be appointed and resourced at the start of an emergency to co-ordinate IYCF-E practices and ensure the implementation of policies and it should be conveyed to all agencies working in the area[13]<br>3. In emergencies, donations of BMS are not needed and may put endanger infant lives due to poor hygienic conditions[2, 4, 10, 12, 13, 23]<br>4. If emergencies occur in places where there was already high infant formula use, promotion of IYCF-E can be even more difficult and WHO, and its developmental partners, along with local authorities and/or the national Nutrition Cluster (if activated) should ensure that appropriate IYCF-E is adequately promoted, protected and supported[2, 10, 12, 23]<br>5. Interventions should be undertaken to increase the prevalence of appropriate IYCF-E practices such as culturally-appropriate behavior -change approaches, along with capacity-building, to increase the rate of exclusive breastfeeding[12, 13, 23]<br>6. A joint statement for protection and support of appropriate IYCF-E should be released and ensured that BMS donations and distributions are carefully monitored[2, 4, 7, 12, 13, 23]<br>7. WHO, its developmental partners, local governments and national Nutrition Cluster (if activated) should work on this and provide this information to all staff, potential donors (including governments and the military) and the media, and ensure that no wrong messages are being disseminated both in emergency preparedness and particularly during the early phase of an emergency response[2, 4, 12, 13, 23]<br>8. Breastfeeding and IYCF support should be a major component of all services for mothers, infants and children and measures should be put in place to ensure that their needs are met in the early stages of an emergency[2, 4, 7, 16]<br>9. Support should also be provided to caregivers and infants with special needs (orphans and unaccompanied children)[2, 6, 12, 14-16, 21, 23]<br>10. It should be ensured that artificial feeding is strictly restricted to the targeted group of infants that require it and mothers who need help with breastfeeding are provided lactation support by mobilizing 'local breastfeeding facilitators'[7, 10]<br>11. Lactation should be reinforced by educating mothers to breastfeed every 2-3 hours at 'breastfeeding stations' scattered across refugee sites[7]

		12. One guideline also suggested the use of 'chlorpromazine' to stimulate milk production, according to the protocol and also 'wet nurses' to feed the infant[7]<br>13. Milk banks can also be used as an alternative to BMS and also as a source of employment in emergency setting[7, 10]
**VIII.**	Assessment, intervention and monitoring	1. In emergencies, co-ordinate, promote and carefully monitor optimal feeding in infants and young children[1, 2, 15, 23]<br>2. There should be systematic and comprehensive monitoring systems to track all infant feeding products being distributed[13]<br>3. Conduct mother – child pair assessment of HIV positive mothers and for individual child, assessment for artificial feeding (i.e. Simple Assessment and Full Assessment)[3, 24]<br>4. In areas with high prevalence of HIV, the risk of infant getting HIV via breastfeeding should be weighed against the risk of facing infection and malnutrition as a consequence of not being breastfed[1]<br>5. The prevalence of HIV in the affected population, knowledge of HIV status, and availability of counselling and testing facilities should be assessed (including pre-emergency estimates) using secondary sources and relevant information from health information systems[10]<br>6. In emergencies, implement an IYCF-E program focusing on infant feeding provision, with robust mechanisms that estimate the number of children that don't have access to breast milk and then provide them with targeted supply of infant formula[12]<br>7. Monitor the nutritional status of infants and young children, particularly weight monitoring for those receiving formula feed and assess intake, urination frequency, activity level, whether infant is feeding vigorously and weight gain[6, 10, 12, 15, 24, 26]<br>8. Establish a strong referral system to treat acute malnutrition should the infant's nutritional status deteriorate[2, 12]<br>9. To monitor and to conduct rapid assessments, gather information and statistics regarding: demographic profile, morbidity, mortality, predominant feeding practice, reported feeding problems for infants and young children including problems related to breastfeeding and complementary feeding, pre-crises approach to orphaned children, security risks and availability of conspicuous BMS products and bottles/ teats/ breast pumps[7, 10, 21, 23]<br>10. To monitor and assess, use qualitative methods to gather data regarding:<br>a. Appropriate complementary foods in the general ration or targeted feeding programs<br>b. Maternal and child health facilities including antenatal, delivery, postnatal and child care<br>c. Capacity of potential support-givers including breastfeeding mothers, trained health workers, trained counselors and experienced women from the community<br>d. Factors that may disturb breastfeeding practices<br>e. Key decision-makers at household, community and local health facility level that may influence infant and young child feeding practices<br>f. Cultural barriers affected practices of re-lactation, wet-nursing, etc.<br>g. General health environment including: water and sanitation, housing, facilities of food preparation and cooking[10, 21, 23, 28]<br>11. To monitor and assess, use quantitative methods to gather data regarding:<br>a. Estimated number of unaccompanied and accompanied children under two years of age, pregnant and lactating women<br>b. Statistics regarding morbidity, mortality, and levels of malnutrition<br>c. Information concerning nutritional adequacy of food rations<br>d. Pre-crisis and recent patterns in infant and young child feeding practices<br>e. Availability and management of BMS in accordance to The International Code[2, 10, 21, 23]<br>12. Governments should monitor and apply The International Code collaboratively with the assistance of International agencies such as WHO and UNICEF, NGO's, refugee camp staff, professional groups and customer organizations to ensure that manufacturers and distributors of BMS remain within the scope of the established Code[2, 10, 25]

	13. Manufacturers and distributors should monitor their market prices and the practices of their marketing personnel in accordance to The Code. Non-governmental organizations (NGO's) along with professional groups, institutions and concerned individuals should monitor and criticize manufacturers and distributors that don't follow the principles of The Code[10, 25]<br>14. Review and monitor the following:<br>a. Advice and knowledge regarding breastfeeding and BMS usage<br>b. Estimate the number of women breastfeeding, weaning and incorporating the use of BMS and bottle in the feed of their infant and young children<br>c. Constraints associated with hygienic BMS preparation<br>d. Availability and management of BMS[4, 7, 10, 20, 21, 23, 25]<br>15. Promote importance of breastfeeding and optimal hygiene practices especially handwashing before preparation of BMS. We should also dispel myths among mothers regarding breastfeeding[28]<br>16. Capacity Building of IYCF staff and outreach workers on nutrition (optimal IYCF practices, lifesaving IYCF practices, rapid IYCF assessment and detection of poor IYCF practices)[2]
**IX.** Breastfeeding, HIV, and other considerations	1. Generate policies on empowering women with HIV on decision making and infant feeding[8]<br>2. IYCF staff should take appropriate measures to prevent mother-to-child transmission of HIV, as well as focus on improving child survival from HIV[2]<br>3. Promote the use of optimal infant and young child feeding guidelines when the HIV status of the mother is unknown or she is HIV negative[2-4, 10, 23, 24, 29]<br>4. In case of unavailability of HIV-testing, it is recommended to breastfeed the infant for six months, followed by adequate complementary feeding and continued breast feeding for two years[24, 29]<br>5. Mothers should know their HIV status and receive appropriate counselling. Those diagnosed as HIV positive should make an informed decision about feeding options by balancing the prevention of HIV transmission with the nutritional requirements of infants[1, 2, 4, 8, 10, 12, 23, 24, 29]<br>6. HIV positive mother should exclusively breastfeed her child for first 6 months of life unless replacement feeding is affordable, sustainable and safe for their infants. In case, replacement feeding is not acceptable, then complementary feeding with continued breastfeeding at 6 months is recommended, while mother and baby will be assessed regularly[2, 10, 24]<br>7. Supportive arrangements and personal attachment for HIV positive mothers helps to reduce isolation, build confidence, reduce conflicting messages, encourage age appropriate feeding, provide privacy and educate family members[24]<br>8. Ensure access to sustainable medical care for mothers with an HIV positive status by supporting the provision of ART and ARV. If due to emergencies, the supply of these medications is hindered then immediate action should be taken for its re-establishment[12, 23, 24, 29]<br>9. In circumstances during acute emergencies, when Antiretroviral Drug (ARVs) are unavailable, it is recommended to breastfeed HIV-exposed infants to increase his/her survival[2, 3]<br>10. In emergency contexts, HIV-positive mothers should be supported to initiate or continue exclusive breastfeeding/ continued breastfeeding with adequate complementary feeding depending upon the age of the infant. The risk of infection or malnutrition through the use of Breast Milk Substitutes (BMS) outweighs the risk of HIV transmission through breastfeeding[2, 8, 12, 21, 23, 24]<br>11. Support replacement feeding *only* when this option meets the AFASS criteria, i.e. acceptable, feasible, affordable, sustainable and safe[3-5, 23, 24, 29]<br>12. All HIV- positive mothers should receive full support and get regular follow ups[2, 29]<br>13. HIV-positive and caregivers of children born to HIV-positive mothers, who have chosen to discontinue or not breastfeed, should be provided with targeted, appropriate, breast milk substitutes (Ready to Use Formula (RUIF) and Powdered Infant Formula (PIF)[2, 12]<br>14. Promote and support specific counseling concerned with risks of mixed feeding and HIV transmission. Additionally, ensure the provision of Safe BMS kits, i.e. adapted to the type of BMS administered[2, 12]

	15. Services and activities linked to the Prevention/Elimination of Mother-to-Child Transmission (E/PMTCT) should be provided routinely as a part of nutritional interventions[2, 12]<br>16. Measures taken by ICYF-E staff should be sensitive and should avoid actions that may exacerbate any HIV-related stigma[2, 12]<br>17. Wet nursing should also be considered in case of HIV positive mothers and for infants who have lost their mothers. It should be administered by any person (other than mother). The wet nurse should be counselled before and after wet nursing to prevent her from catching infection[10, 12, 24, 29]<br>18. WHO recommends flash heated breastmilk rather than boiling breast milk to prevent significant nutritional damage of breastmilk[29]

References:

1. WHO. Guiding principles for feeding infants and young children during emergencies. 2004.
2. UNHCR, children St. Infant and Young Child Feeding in Refugee Situations:

A Multi-Sectoral Framework for Action. 2018 May, 2018.
3. UNHCR. Infant and young child feeding practices

Standard Operating Procedures for the Handling of Breastmilk Substitutes (BMS) in Refugee Situations for children 0-23 months. 2015 August 2015.
4. UNHCR. UNHCR Policy Related To The Acceptance, Distribution And Use Of Milk Products In Refugee Settings 2006.
5. Save the Children U, Food Security and Nutrition Network. ICFY-E Toolkit: Summary of Feeding the Non-breastfed child 6- 24 months of Age 2007.
6. Refugees UHCf. Interim Operational Considerations for the feeding support of Infants and Young Children under 2 years of age in refugee and migrant transit settings in Europe. 2015.
7. OXFAM. Feeding in Emergencies For Infants Under Six Months: Practical Guidelines 1996.
8. Machel G. Impact of Armed Conflict on Children. United Nations; 1996 26 August 1996.
9. Joint UNICEF U, WFP, WHO Statement Policy on infant feeding in the Balkan Region.
10. ENN. Infant Feeding in Emergencies: Policy, Strategy and Practice. 1999 May 1999.
11. Association ILC. Breastfeeding: A Vital Emergency Response. Are You Ready? . 2009. p. 2.
12. ACF-International. Infant and Young Child Feeding in Emergencies. 2015 December 2015.
13. Borrel A, Taylor A, McGrath M, Seal A, Hormann E, Phelps L, et al. From policy to practice: challenges in infant feeding in emergencies during the Balkan crisis. Disasters. 2001;25(2):149-63.
14. Program WIEPoB. Infant and Young Child Feeding in Emergency Situations. 2005.
15. Pediatrics AAo. Infant feeding in disasters and emergencies. 2015.
16. Save the Children U, Food Security and Nutrition Network. IYCF-E Toolkit: Summary of Operational Guidance on ICF-E Key Points and WHA Resolution. 2007.
17. UNHCR. HOW TO GUIDE reproductive health in refugee setting (Strengthening Safe Motherhood Services). 1999 November 1998.
18. Children St. Assessment of infant and young child feeding practices among refugees on Lesvos Island, Greece. 2016.
19. Focus I. The Code and infant feeding in emergencies. 2009 May 2009.
20. USAID TSG, International Medical Corps Camp-based Barrier Analysis of Early Initiation of Breastfeeding, Iron-rich Food Consumption, and Early Antenatal Care Seeking Behaviors of Syrian Refugees in Azraq Camp, Jordan. 2016.

21. WHO, UNICEF, LINKAGES, IBFAN, ENN. Infant feeding in emergencies Module 1 (for emergency relief staff). 2001 November 2001.
22. WHO. WHA RESOLUTION 47.5

Infant and young child nutrition. 1994.
23. Group IC. Infant and Young Child Feeding in Emergencies Operational Guidance for Emergency Relief Staff and Programme Managers

2017 October 2017.
24. ENN, IBFAN-GIFA, Fondation Terre des hommes, Action Contre la Faim, CARE USA, LINKAGES, et al. Infant feeding in emergencies Module 2 Version 1.1 (for health and nutrition workers in emergency situations). 2007.
25. WHO. International Code of Marketing of Breast-milk Substitutes. Geneva; 1981.
26. ACF-International. Baby friendly spaces

Holistic approach for Pregnant, Lactating Women anf their very young children in Emergency. 2014 December 2014.
27. Action Against Hunger U. Infant Feeding and Weaning Report 2000.
28. UNICEF. UNICEF Guidance on the provision and use of breastmilk substitutes in humanitarian settings. 2018 June 2018.
29. UNHCR. Guidance on Infant feeding and HIV in the context of refugees and displaced populations. 2008 April 2008.
