# Supplementary material for: Systematic review of infant and young child feeding practices in conflict areas: what the evidence advocates
Source: BMJ Open. 2020 Sep 13;10(9):e036757. doi: 10.1136/bmjopen-2020-036757 (PMC7488834; doi:10.1136/bmjopen-2020-036757)
Supplement: Author's manuscript [file bmjopen-2020-036757.draft_revisions.pdf]

BMJ Open is committed to open peer review. As part of this commitment we make the peer review history of every article we publish publicly available.

When an article is published we post the peer reviewers' comments and the authors' responses online. We also post the versions of the paper that were used during peer review. These are the versions that the peer review comments apply to.

The versions of the paper that follow are the versions that were submitted during the peer review process. They are not the versions of record or the final published versions. They should not be cited or distributed as the published version of this manuscript.

BMJ Open is an open access journal and the full, final, typeset and author-corrected version of record of the manuscript is available on our site with no access controls, subscription charges or pay-per-view fees (http://bmjopen.bmj.com).

If you have any questions on BMJ Open's open peer review process please email info.bmjopen@bmj.com

BMJ Open

Ensuring Optimal Infant and Young Child Feeding Practices in conflict areas: What the evidence advocates

Journal:	*BMJ Open*
Manuscript ID	bmjopen-2020-036757
Article Type:	Original research
Date Submitted by the Author:	01-Jan-2020

Complete List of Authors:	Rabbani, Amna; Aga Khan University<br>Peera, Zahra; Aga Khan University, Paediatrics<br>Siddqui, Faareha; Institute of Business Administration<br>Das, Jai K.; Aga Khan University<br>Bhutta, Zulfiqar; Hospital for Sick Children

Keywords:	PUBLIC HEALTH, Health policy < HEALTH SERVICES ADMINISTRATION & MANAGEMENT, Community child health < PAEDIATRICS

SCHOLARONE™
Manuscripts

I, the Submitting Author has the right to grant and does grant on behalf of all authors of the Work (as defined in the below author licence), an exclusive licence and/or a non-exclusive licence for contributions from authors who are: i) UK Crown employees; ii) where BMJ has agreed a CC-BY licence shall apply, and/or iii) in accordance with the terms applicable for US Federal Government officers or employees acting as part of their official duties; on a worldwide, perpetual, irrevocable, royalty-free basis to BMJ Publishing Group Ltd ("BMJ") its licensees and where the relevant Journal is co-owned by BMJ to the co-owners of the Journal, to publish the Work in this journal and any other BMJ products and to exploit all rights, as set out in our licence.

The Submitting Author accepts and understands that any supply made under these terms is made by BMJ to the Submitting Author unless you are acting as an employee on behalf of your employer or a postgraduate student of an affiliated institution which is paying any applicable article publishing charge ("APC") for Open Access articles. Where the Submitting Author wishes to make the Work available on an Open Access basis (and intends to pay the relevant APC), the terms of reuse of such Open Access shall be governed by a Creative Commons licence – details of these licences and which Creative Commons licence will apply to this Work are set out in our licence referred to above.

Other than as permitted in any relevant BMJ Author's Self Archiving Policies, I confirm this Work has not been accepted for publication elsewhere, is not being considered for publication elsewhere and does not duplicate material already published. I confirm all authors consent to publication of this Work and authorise the granting of this licence.

For peer review only

Ensuring Optimal Infant and Young Child Feeding Practices in conflict areas: What the evidence advocates

Amna Rabbani[1], Zahra Ali Padhani[1], Faareha A Siddiqui[2], Jai K Das[1], Zulfiqar A Bhutta[3,4]

1. Division of Women and Child Health, Aga Khan University, Karachi, Pakistan
2. Institute of Business Administration, Karachi, Pakistan
3. Center of Excellence in Women and Child Health, Aga Khan University, Karachi, Pakistan
4. Centre for Global Child Health, The Hospital for Sick Children, Toronto, Canada

Amna Rabbani - amnarabbani@gmail.com
Zahra Ali Padhani - zahra.feroz@aku.edu
Faareha A Siddiqui - faareha.siddiqui@gmail.com
Jai K Das - jai.das@aku.edu
Zulfiqar A Bhutta - zulfiqar.bhutta@sickkids.ca

Corresponding Author

Zulfiqar A Bhutta
Centre for Global Child Health, The Hospital for Sick Children
686 Bay Street, Toronto, ON M5G 0A4, Canada
Tel: +1 416 813 7654x328532
Email: zulfiqar.bhutta@sickkids.ca

For peer review only

ABSTRACT

Background

Breastfeeding in conflict settings is known to be the safest way to protect infant and young children from malnourishment and increased risk of infections. This comprehensive review assesses the evidence on infant and young child feeding (IYCF) practices in conflict settings.

Methodology

We conducted an extensive search from the year 1980 until August, 2019. We included studies conducted in armed conflict settings and within five years of cessation of conflict; assessing IYCF practices, barriers, programs and guidelines to promote and improve IYCF practices. Two review authors independently assessed and screened studies for eligibility and extracted data, followed by a descriptive and thematic analysis.

Results

We included 56 studies in our review and broadly classified into four pre-determined sections; epidemiology, barriers/enablers, programs/interventions and implementation guidelines. Epidemiological evidence showed that IYCF practices were generally poor in conflict settings, with median prevalence of exclusive breastfeeding at 25%, continued breastfeeding 29%, and bottle-feeding rates 58.3%, and the prevalence of malnutrition and infectious diseases was high.

IYCF practices were affected by displacement, stress, maternal malnutrition and mental health, family casualties and free distribution of BMS. Several interventions were implemented including training of health workers, lactation-support service, BFHI, mother-baby friendly spaces and support groups. The evidence suggests that to improve IYCF, it is imperative to disseminate the guidelines and implementation plan early with coordination between all relevant stakeholders. A single lead entity should be responsible for implementing and monitoring IYCF guidelines and regulating distribution of BMS. There should be adequate dedicated funds and capacity building of all staff. Formula feeding should be the last resort for individuals after expert evaluation.

Conclusion

The evidence generated from our study shows that IYCF practices are generally poor in conflict settings but with much potential for improvement with effective interventions and proper implementation of IYCF guidelines.

Key Words

Breastfeeding, IYCF, conflict, epidemiology, barriers, programs, guidelines

Word Count: 6061 words

For peer review only

STRENGTHS AND LIMITATIONS OF THIS STUDY

1. This is a first comprehensive review of Infant and young child feeding practices in conflict settings looking at the evidence on the current practices of breastfeeding and complementary feeding and specific barriers to adapting optimal IYCF practices. This review also explores the evidence of the effective strategies to improve IYCF practices and the evidence from implementation guidelines to guide on what and how should we approach IYCF in such contexts.
2. The review highlights the high burden of disease and undernurition and low uptake of standard IYCF practices in children affected by conflict.
3. There is scarcity of evidence as many programs in conflict contexts are not reported and most of the included studies did not comprehensively evaluate the program impact.
4. The evidence from the strategies/interventions implemented in conflict areas to improve IYCF practices though weak, provides important insights to interventions to focus to improve IYCF practices.
5. The synthesis of the implementation guidelines framed by various stakeholders with experience in working in such contexts underscores the need to emphasize on IYCF and provides a roadmap how to holistically approach this.

For peer review only

INTRODUCTION

Optimal infant and young child feeding practices (IYCF) play a critical role in determining the nutritional status, health, growth and development of children, along with improving the health of mothers[1-4].

The current guidelines suggest breastfeeding to be initiated within the first hour of birth and infants exclusively breastfed for the first six-months of life, i.e. receive only breast milk, with the exception of oral rehydration solutions and drops/syrups of vitamins, minerals and medicines[2, 7, 45]. Exclusive breastfeeding (EBF) offers the required nourishments for normal growth and development till six months of age[1]; thereafter safe, timely and nutritionally adequate complementary foods should be added to the diet of infants, along with continued breastfeeding up to two years of age[2, 7, 45].

Children who have been breastfed for longer periods of time tend to exhibit lower odds of infectious morbidity and mortality[5], as infants who are not breastfed have six-fold greater risk of infections related in the first two-months of life when compared to infants that have been adequately breastfed[8]. The current evidence suggests that high-income countries (HICs) practice shorter duration of breastfeeding (<20%) compared to low and middle- income countries (LMICs)[5]. However, even within LMICs, approximately only 37% of infants younger than six-months are exclusively breastfed[5]. Just scaling up and promoting breastfeeding to an optimal level could possibly prevent 823,000 annual deaths in children under the age of five[5] and 13.8% of these under two years of age[5].

During times of armed conflict and emergencies; vulnerable groups including children bear the greatest negative consequences. The onset of conflict increases death rates by up to 24 times, with adverse effects especially for children under the age of five years[11]. Newborns are specifically at a higher risk of dying if they are poor, exposed to unsafe environments, or if they are within a conflict setting[8]. Armed conflict significantly impacts breastfeeding practices with lower rates of breastfeeding observed in war-torn areas[6]. Before Lebanon's conflict in 2006, approximately 27% of mothers exclusively breastfed for the first four-months of life and after the conflict escalated, the breastfeeding practices were severely affected with most mothers discontinuing breastfeeding altogether or initiating mixed feeding and/or reduced breastfeeding[10].

In emergency situations; breastfeeding is of crucial importance because it is regarded as the safest way to protect infants and young children from infections and malnourishment[9]. This can be corroborated by the fact that during emergency situations; mortality rates of artificially-fed infants are greatly elevated in comparison to breastfed babies[8], as the risk mortality due to diarrhea and other infectious diseases are 20 times higher than infants who have been exclusively breastfed[10]. This is due to prevalent unhygienic conditions coupled with lack of safe water and facilities to sterilize feeding bottles and prepare formula safely. Support for optimal breastfeeding and re-lactation should be the first choice of intervention during conflict situations to mitigate feeding problems for infants and young infants[12] and should not be undermined by inappropriate distribution of Breast Milk Substitutes (BMS)[1, 11, 12]. There is currently no existing comprehensive review for IYCF in conflict settings. We in this paper aim to generate the evidence on IYCF

For peer review only

practices, factors associated with IYCF, to assess the evidence of the interventions undertaken and guidelines to improve IYCF practices amongst populations affected by conflict.

CONCEPTUAL FRAMEWORK

During armed conflicts, IYCF practices are disrupted due to displacement, casualties, lack of resources, stress, and malnutrition, as well as due to unregulated BMS donations by agencies. We developed a conceptual framework to guide this review and highlighting the IYCF practices and factors responsible for improving IYCF practices in an armed conflict setting (Figure 1). It also focuses on the evidence of current IYCF practices, factors associated with improving IYCF and the evidence from interventions and programs implemented in these settings. We also explored existing implementation guidelines and program recommendations for improving IYCF practices from different agencies working in such settings.

METHODOLOGY

We conducted a systematic review for the available published and grey literature looking at four domains including epidemiology (coverage of key IYCF and malnutrition indicators), enablers/barriers (for recommended IYCF practices), interventions/programs (effectiveness in improving IYCF practices) and implementation guidelines to improve IYCF practices in conflict settings.

Eligibility Criteria

We included studies assessing the impact of conflict on IYCF practices and enablers/barriers, interventions/programs and guidelines to promote or facilitate IYCF in conflict settings. We included studies conducted in an armed conflict setting, defined as 'a political conflict in which armed combat involves the armed forces of at least one state (or one or more armed factions seeking to gain control of all or part of the state) and in which people have been killed by the fighting during the course of the conflict'[13]. We included studies conducted within five years of the cessation of conflict and included primary research articles, reports and grey literature and policy and guidelines. We included all studies conducted in conflict settings of low, low-middle income and upper-middle income countries classified by World Bank (WB)[14], during the period of 1980 to 2018 and we also included studies conducted in refugee camps in HIC.

We excluded studies conducted in HICs apart from refugee camps and on military personnel. Clinical studies (exclusively looking at microbiological/laboratory outcomes/screening or diagnostic test evaluations or surgical techniques/outcomes) and mathematical modeling or economic study (with no empirical data/information), systematic and literature reviews were excluded from the study. We also excluded studies conducted in humanitarian emergencies apart from armed conflict.

Data Management

We searched PubMed and CENTRAL using our search strategy constructed by using Medical Subject Headings (MESH) and key words (Box 1). The bibliographies of relevant systematic reviews

For peer review only

and included studies were searched to identify the missing records in the database search. We only included articles in English language. Additionally, a grey literature search was conducted on Google and websites and publications of relevant agencies such as WHO (GINA), UNICEF, UNHCR, International Baby Food Action Network, IBFAN, Emergency Nutrition Network (ENN), Baby Milk Action, Save the Children, Action contre la faim (ACF) and Wellstart International. We also searched for additional data by entering titles of all included studies on Google and reviewing the first 10 pages to include any relevant missing studies.

**Box 1: Search Strategy**
("Disasters"[Mesh] OR "Disaster Victims"[Mesh] OR "Natural Disasters"[Mesh] OR mass disaster OR catastrophe* OR crisis OR crises OR war OR "afghan campaign" OR "Armed Conflicts"[Mesh] OR conflict OR conflicts OR war OR avalanche* OR cyclone* OR drought* OR earthquake* OR flood* OR hurricane* OR landslid* OR land slide OR land slides OR mudslid* OR mudslides OR mud slide OR mud slides OR storm* OR tornado* OR tsunami* OR typhoon* OR volcano*) AND ("Refugees"[Mesh] OR refugee OR asylum seeker* OR returnees OR "IDP" OR "IDPs" OR internally displaced person* OR mother* OR "lactating" OR "lactating mother" OR "lactating mothers") AND (Breastfeed* OR "breast feeding" OR "breastfed" OR "exclusive breastfeeding" OR "human milk" OR "lactation" OR infant feed* OR complementary feed* OR "IYCF" OR "Infant and Young Child Feeding" OR "early initiation" OR "colostrum" OR "breastfeeding practices" OR "breastfeeding promotion" OR "breastfeeding facilitation" OR barriers OR "breast milk collection" OR "breast milk collections" OR "breast milk expression" OR "breast milk expressions" OR "breast pumping" OR "breast pumpings" OR "breastmilk collection" OR "breastmilk collections" OR "breastmilk expression" OR "breastmilk expressions" OR "milk bank" OR "feeding bank")

After running the electronic search, all records were imported to EndNote software[15] and were de-duplicated prior to title and abstract screening. Two reviewers independently screened titles and abstracts, followed by full text screening. All the discrepancies were resolved after discussion at each stage and a third reviewer was contacted incase two reviewers were unable to reach a consensus. Data was extracted independently by two reviewers from the included studies in an excel sheet after the full text screening. We calculated the median of all IYCF and malnutrition indicators from the studies identified and conducted a descriptive and thematic analysis of included studies to explore and synthesize information on the contextual factors and intervention and recommended implementation strategies.

We obtained ethical approval of this study from the Ethical Review Committee of Aga Khan University, Karachi and the National Bioethics Committee, Pakistan. The funding for this review was received from the Family Larsson-Rosenquist Foundation.

Patient and Public Involvement

This research was done without patient involvement. Patients were not invited to contribute to the writing or editing of this document for readability or accuracy.

RESULTS

We identified a total of 56 studies (Figure 2) which were broadly classified into the pre-determined four sections; epidemiology, enablers/barriers, programs/interventions and implementation guidelines.

Epidemiology

IYCF Practices

Twenty-four studies reported on the prevalence of breastfeeding and the burden of disease in different conflict-affected regions[9, 11, 16-37]. Eighteen were reports[9, 11, 16-18, 23, 26-37], five were cross sectional studies[19, 21, 22, 24, 25], and one was a cohort study[20]. Seven studies were conducted in Middle East[9, 17, 27-29, 31, 33], six in Europe [16, 17, 24, 25, 34, 35], five in Africa[16, 18-20, 36], four in Asia[26, 30, 32, 37] and two reported on prevalence in more than one region which included Africa[11], Asia[11, 23] and Middle East[23]. The included studies collected data through various sources and methods; five studies included data from national assessments done by the WHO and other developmental partners[11, 17, 23, 28, 29], five conducted cross sectional household surveys[21, 22, 24, 33, 34], four collected data from multiple indicator cluster survey (MICS) [9, 25-27], four conducted SMART surveys[16, 18, 30, 37], one distributed questionnaires[19], one collected data from national surveillance system registry[20], one through Disease Early Warning System (DEWS) surveillance network[32] and two did not specify[31, 36].

Twenty studies reported on IYCF according to the WHO IYCF indicators which are summarized in Table 1[16-28, 30, 31, 33-37]. These studies analyzed IYCF indicators divided into WHO eight core indicators (early initiation of breastfeeding, exclusive breastfeeding under six months, continued breastfeeding at one year, introduction of solid, semi-solid or soft foods, minimum dietary diversity, minimum meal frequency, minimum acceptable diet and consumption of iron-rich or iron-fortified foods) and seven optional indicators (children ever breastfed, continued breastfeeding at two years, age-appropriate breastfeeding, predominant breastfeeding under six months, duration of breastfeeding, bottle feeding and milk feeding frequency of non-breastfed children)[38]. The median prevalence of early initiation of breastfeeding in conflict-affected areas was 51% (range: 31.3% to 85%), EBF was 25% (5.5% to 77.1%), introduction of solid, semi-solid or soft foods was 71.1% (40.7% to 98.6%), minimum dietary diversity was 60.3% (9.2% to 79.4%), children ever breast-fed was 92% (62.8% to 98.4%), continued breastfeeding at two years was 29% (9% to 66%), age-appropriate breastfeeding was 43.2% (19.5% to 77.8%), predominant breastfeeding was 31.3% (7.1% to 77.3%) and bottle feeding was 58.3% (31.8% to 71.4%). The median prevalence of global acute malnutrition (GAM) was 9.95% (2.6% to 25.1%), moderate acute malnutrition (MAM) was 4.0% (0.27% to 25%) and severe acute malnutrition (SAM) was 1.5% (0.15% to 5.3%).

Two studies[19, 24] reported on association of breastfeeding with malnutrition in displaced Bosnian[24] and Saharawi children[19]. Infants were more likely to be malnourished (MUAC <125mm) who were never breastfed (Odds Ratio (OR): 1.78, 95% CI: 1.26, 2.52) or who were not breastfed for atleast four months (OR: 1.45, 95% CI: 1.02, 2.07) than those who were ever breastfed or were breastfed for four months. Malnutrition persisted among infants who were not exclusively breastfed and infants who were breastfed for less than five to six months were more likely to be malnourished (OR:1.98, 95% CI: 1.01, 7.35) than those who were breastfed for more than six months[24]. After adjusting for diseases, mother's BMI and child's age; prevalence of underweight and wasting was low among children who were predominantly or exclusively breastfed (MD: 0.62, 95%CI: 0.10, 1.13 and 0.41, 95% CI: -0.08, 0.91 respectively)[19].

Burden of disease

Three studies conducted in countries affected by armed conflict reported on morbidity and mortality of children under five years of age[11, 18, 20]. The crude mortality rate (CMR) was 2.78 deaths/10,000 per day in South Sudan during the crisis and the under-five mortality rate (U5MR) was 0.71 deaths/10,000 per day[18]. During the conflict, the U5MR was 58 in Guatemala, 250 in Afghanistan and 316 in Sierra Leone[11]. The infant mortality rate (IMR) in 43 in Guatemala, 165 in Afghanistan and 182 in Sierra Leone[11]. A study from Guinea Bissau reported a six-fold higher mortality (MR:5.73, 95%CI: 2.40, 13.71) among weaned children aged 9-35 months compared to breastfed children during the war[20] and this could be attributed to overcrowding, higher infection prevalence during the war, and poor access to care[20]. Five studies reported on prevalence of diarrhea in under-five children living in conflict-affected countries[11, 21, 30, 32, 37]; 28.9% children suffered from diarrhea in Yemen (Taiz governorate)[37], 30% in Guatemala[11] and during an outbreak 73.8% internally displaced children in Pakistan had diarrhea[32]. Mortality due to diarrhea among Afghan refugees in Pakistan was 22.3%[30]. Due to conflict, mortality from diarrhea increased from 20.0% to 87.0% amongst Rwandan refugees in Zaire in 1994, from 20% to 39.0% amongst IDPs in Somalia in 1992, and from 22.9% to 74% in Kurdish refugees in Iraq in 1991[11]. However, the mortality rates due to diarrhea amongst Rwandan refugees in Nepal from 1992-1993 remained the same (22.9%) and decreased amongst residents in eastern Democratic Republic of Congo (DRC) in 2000 from 20.0% to 11.0% during conflict[11]. This study also reported measles, meningitis, malaria and respiratory infection as major reason of under-five mortality during conflict[11]. Mortality rates due to measles increased from 10.1% to 36.5% amongst IDPs in Somalia in 1992 during conflict[11]. Deaths due to respiratory infections also increased from 26.2% to 41.4% among Rwandan refugees in Zaire in 1994, and deaths due to malaria increased from 15.5% to 26.0% in residents in eastern DRC in 2000[11].

Enablers/ Barriers

Eighteen studies reported on enablers/barriers to IYCF practices in conflict settings (Figure 3) [9, 17-19, 21-29, 34, 39-42]. Of these, 12 were reports[9, 17, 18, 23, 26-29, 34, 40-42] and six were cross sectional studies[19, 21, 22, 24, 25, 39]. Seven studies were conducted in Europe[21-25, 34, 41], five in Middle East[9, 17, 27-29], three in Africa[18, 19, 39], one in Asia[26], one study was conducted in both Asia and Europe[40], and one failed to report[42]. Nine studies reported on refugees[9, 17, 19, 23, 27, 28, 36, 40, 41], four on IDPs[18, 21, 22, 29], three on

For peer review only

non-displaced residents[24, 26, 39] and two failed to report on the target population[25, 42]. Nine studies were conducted in camps[9, 17, 19, 23, 27, 28, 36, 40, 41], seven in community[18, 21, 22, 24, 26, 29, 39] and two did not report[25, 42].

Breastfeeding declined significantly in regions with high levels of conflict-related violence relative to areas which were considered the 'safest areas' in Iraq[26]. A mother residing in dangerous areas of Iraq was 17.4% more likely to stop breastfeeding compared to mothers living in the safe areas[26]. In Bosnia-Herzegovina, illness of mother/baby disrupted breastfeeding and a few mothers made a personal decision not to breastfeed[25]. Other reasons that negatively affected breastfeeding practices in conflict affected areas were unavailability of trained healthcare professionals, and disruption in knowledge created by violent conflict[17, 26].

Ten studies reported the common misconception amongst refugees, media, and humanitarian workers that mothers produce poor quality, insufficient milk due to malnourishment and stress induced as an impact of war[17, 18, 21, 23, 26, 28, 29, 34, 40, 42]. These misconceptions led to mothers feeding undiluted animal milk to infants younger than four months [18]. In Bosnia-Herzegovina and Jordan, health professionals discouraged mothers from breastfeeding due to significant maternal weight loss during conflict[17, 21]. Breastfeeding practices of mothers were affected by mental trauma from fighting and due to war-related casualties in male members of the family[18, 24, 26, 28, 40]. In Bosnia-Herzegovina, infants were less likely to be breastfed for more than 4 months if they resided closer to the area of conflict and if the household was not receiving remittances from abroad[24].

Refugees in Lebanon and Greece considered BMS as necessary food for infants during emergencies and many mothers pursued mixed feeding because of the physician's prescription of infant formula[27, 34, 42]. The study on Syrian refugees in Lebanon stated that in children above six months, child refusal of food was due to inadequate knowledge regarding complementary feeding practices and the quantity of solid food that a young child should receive per day[28]. In Azraq refugee camp in Jordan, poor quality food and lack of iron-rich foods in the market was the reason for low consumption of iron-rich foods by the infants[33]. In Bosnia-Herzegovina and Lebanon, mothers introduced tea, sugar, water, juice and infant formula in the feeding practice to spend less time breastfeeding which negatively influenced the rate of EBF[25, 28]. Two studies' mentioned 'lack of milk' as the reason for early weaning by mothers[25, 34], and many mothers wrongly thought that weaning can't be reversed[42]. Another reason for suboptimal breastfeeding practices, mentioned by four studies from Iraq, Greece, Lebanon and Jordan, was the heavy marketing of artificial feeding and big push from infant formula companies, which made mothers believe that it was better[9, 27, 34, 40].

Studies conducted in Algeria, Jordan, and Northern Uganda mentioned factors leading to delayed initiation of breastfeeding by refugee mothers as home delivery, C-section, breast pain, discarding initial milk, illiteracy, lack of support from medical staff, previous experience, mother being ill and being the sole person responsible for decision of initiating breastfeeding[19, 33, 39]. Mothers used pre-lacteal feeds before initiating breastfeeding, as colostrum was believed to be of low nutritional value and was considered 'dirty' and harmful[19, 39]. The report from Iraq and Greece mentioned

For peer review only

that many mothers assumed that their milk was ‘dirty’ if a previous infant died while breastfeeding, resulting in them refraining from breastfeeding the next baby[40].

Early introduction of complementary food and infant formula also led to suboptimal breastfeeding practices[25, 27, 28, 34, 42]. They were introduced as early as 1-3 months because of the misconception that breastmilk was not sufficient for meeting the nutritional requirements of the infants[25, 28]. Seven studies focusing mainly on refugees from conflict affected countries, explored reasons for mix feeding or fully artificial feeding infants[17, 25, 27, 28, 34, 40, 42]. Breastfeeding practices were influenced by religious and cultural determinants and frequent migrations[26, 34]. Studies from Jordan and Bosnia-Herzegovina mentioned about the role of grandmothers in influencing breastfeeding practices and barriers[17, 25]. Grandmothers often pressurized mothers to feed BMS (water and herbs) to infants and asked mothers to follow their traditional approach. Moreover, there was a cultural belief that formula milk is safer than breast milk[17, 40].

Programs/ Interventions

Breastfeeding in an emergency is known to be the safest way to protect infants and young children from an increased risk of infection and undernutrition[9]. To reduce the burden of disease and poor IYCF practices; several programs were launched in conflict-affected countries by different organizations (WHO, international NGOs (INGOs) and local NGOs (LNGOs)). The interventions were mostly based on the WHO guidelines to promote, protect, and support appropriate IYCF practices.

We included fifteen studies which reported on promotion of optimal IYCF practices in conflict settings (Table 2) [9, 11, 16-18, 25, 27-29, 31, 32, 36, 40, 41, 43]. Fourteen were reports[9, 11, 16-18, 27-29, 31, 32, 36, 40, 41, 43] and one was a cross-sectional study[25]. These programs were formulated based on literature review, document review and extensive discussions of stakeholders during conferences, through semi-structured interviews (e.g., open group discussions of mothers/ caregivers), and knowledge, attitude and practices (KAP) surveys. Six studies were conducted in Middle East[9, 17, 27-29, 31], four in Africa[16, 18, 36, 43], three in Europe[11, 25, 41], one in Asia[32] and one study was conducted at more than one place i.e. Europe and Asia[40]. Eight of these focused on refugees[9, 11, 17, 27, 28, 36, 40, 41], three involved IDPs[18, 29, 32], one reported on both hosts and IDPs[43] and three studies failed to report on it[16, 25, 31]. Six studies were conducted in refugee camps[11, 27, 28, 36, 40, 41], five in community[16, 18, 29, 31, 43], three in both camps as well as in clinics[9, 17, 32], and one study failed to report on it[25].

The specific interventions in these programs included capacity building of healthcare staff, education and awareness activities for mothers, community mobilization, provision of baby friendly spaces, lactation support services, complementary and safe artificial feeding support services, and baby friendly hospital initiative (BFHI)[9, 11, 16-18, 25, 27-29, 31, 32, 36, 40, 41, 43]. Five studies focused only on one intervention for promotion of optimal breastfeeding practices[25, 29, 31, 32, 41], while rest had a multi-pronged approach[9, 11, 16-18, 27, 28, 36, 40, 43]. Most of the studies/programs did not evaluate the programs and its impact on IYCF practices or health and nutrition indicators. A follow-up survey after three months of intervention, assessed breastfeeding practices among Syrian refugees in Jordan, which showed an increase in breastfeeding knowledge from 49.5% in 2013 to 71% in community and 91.2% in health facility in 2014. However, no improvement in

For peer review only

breastfeeding practices was observed[17]. Similarly, in Greece, formula use in the refugee camps decreased from 60% to 0% within 6 months, through their Infant and Young Child Feeding Program in Emergencies (IYCF-E)[40].

Implementation Guidelines

We included 30 implementation guidelines by different organizations in our review, mentioning key policies and operational guidelines to be followed during conflicts to ensure optimal IYCF practices[1, 2, 33-35, 42, 45-68]. Eight guidelines were by INGOs[2, 34, 35, 42, 47, 52, 60, 68], nine by UN agencies[45, 48, 51, 54, 56-58, 66, 67], and three by academic organizations[49, 50, 65], while ten guidelines were formulated by collaboration between developmental partners and INGOs[1, 33, 46, 53, 55, 59, 61-64]. These implementation guidelines were formulated after conducting random cluster surveys (including quantitative and qualitative analysis), meetings to gathering empirical evidence, past emergency experiences, and technical guidance from community, stakeholders, development and implementation partners.

We included implementation guidelines from 1981 through 2018. Only one guideline was from 1981-1990[51], seven were from 1991-2000[35, 48, 52, 54, 55, 67, 68], 12 were from 2001-2010[1, 42, 45, 47, 49, 50 53, 56, 57, 63, 64, 69], and ten were from 2011-2018[2, 33, 34, 46, 58-60, 62, 65, 66]. These guidelines focused on refugees and IDPs affected by conflict. We summarized these operational guidelines from different organizations, to be used during conflict settings, using the components from 'WHO's guiding principles for feeding infants and young children during emergencies'[45] (Figure 4).

Breastfeeding

The guidelines on protecting, promoting and supporting breastfeeding were mentioned by 28 publications[1, 2, 33-35, 42, 45-56, 58-68]. They recommend to practice EBF till six months of age, followed by frequent breastfeeding until two years, along with complementary feeding to protect babies from infection, especially in crisis situations. There should be formation of practical guidelines, capacity building of healthcare staff, and continuous flow of funds for the sustainability of programs.

General population (including mothers) should be educated on breastfeeding, its benefits, colostrum use, feeding methods, and consequences of artificial feeding based on 'UNICEF/WHO Baby friendly Hospital's initiative's Ten Steps to breastfeeding'. Moreover, mothers should be counselled not to stop breastfeeding in emergency situations, sickness, and when malnourished. They should be encouraged to practice skin-to-skin contact, initiate breastfeeding within the first hour of birth, and use of pacifiers, artificial nipples and estrogen containing contraceptive pills should be discouraged.

Guidelines also recommend formation of baby friendly spaces and mother to mother support groups to encourage breastfeeding and privacy. There should be a system set in place for identification of newly arriving mothers and infants, registration for rations and referral for immediate assistance. Mothers should be facilitated for re-lactation if separated from the baby or

For peer review only - http://bmjopen.bmj.com/site/about/guidelines.xhtml

experiencing difficulties in breastfeeding. When natural breastfeeding is not possible, wet-nursing and the use of milk banks, should be considered before providing infant formula or home-modified milk.

Breast-Milk Substitutes (BMS)

Twenty-six guidelines reported on the use of BMS in emergency situations[1, 2, 34, 35, 42, 45-56, 58-63, 65, 66, 68]. Donations, procurement, promotion (through advertisements and gifts) and distribution of BMS, bottles and teats should be strictly controlled and should comply with the International Code and World Health Assembly (WHA) regulations, and all violations should be reported (see Box 2). The distribution should be managed by a single designated agency and blanket distribution of BMS should be discouraged in emergency situations, especially where hygienic conditions can't be ensured.

BMS are not recommended for children under 6 months of age and should only be distributed to infants who have no other viable breast milk options i.e. orphans, severe maternal illness or malnutrition (based on established criteria- where distribution can be targeted, the supply chain is secure, and the conditions for safe preparation and use can be met), determined by a qualified health worker trained in IYCF. There should be training of workforce handling BMS and demonstrations should be given to mothers on proper and safe use of infant formula, followed by regular infant health and growth monitoring. Availability of fuel, safe water and equipment should be ensured before BMS distribution and mothers should be advised to use cups instead of bottles for feeding. Moreover, it should be ensured that distribution of BMS to the targeted infant continues for as long as the infant needs (at least 6 months).

Infant formula (generic, unbranded formula) should be distributed with labels in the local language to the infants in need. These labels should have clear instructions on its safe preparation, along with stating the importance of breast milk. Infant formula should not be excluded from the commodities that a mother can access via cash/voucher schemes, but it should have clear information on superiority of breastfeeding. For infants under six months of age, the only suitable BMS is infant formula and condensed milk should not be used for infant feeding and home-modified milk should be the last resort.

Relief workers should ensure that milk products are received and distributed in a dry form and dried milk products are distributed only when pre-mixed with a milled staple food and should not be distributed as a single commodity. Moreover, it should be ensured that dried skimmed milk is not given to infants and for older children, it should be given after fortifying it with vitamin A. Relief and healthcare workers should counsel mothers to avoid baby juices and teas, and they should take appropriate measures to reduce spill-over by ensuring that feeding BMS to a minority of children doesn't undermine breastfeeding practices of the majority. In case of unfavorable circumstances for BMS distribution, an on-site supplementary "wet" feeding program should be conducted in closed spaces under supervision.

For peer review only - http://bmjopen.bmj.com/site/about/guidelines.xhtml

Box 2: Violation of IYCF-E guidelines in conflict settings (Case Studies)

Case Study 1: Campaigning by Blédina company during Hezbollah–Israel Conflict, 2006: Lebanon

A marketing campaign by Bledina (production company of infant formula and complementary foods), after the war in 2006 in Beirut, offered mothers gift packages containing promotional leaflets. The attending pediatrician also gave mothers a card with the Blédina hotline. Moreover, free Blédina gift packages were also distributed at a health center which had a high number of refugees, as a promotional campaign after the war, which was a violation of the IYCF-E guidelines[10, 47].

Case Study 2: Civil Unrest, 2002: East Timor

Healthcare facilities in Dili distributed infant formula donated by a service organization to assist infants who have been orphaned and mothers who are unable to breastfeed, which was a violation of the guidelines because health care facilities should not distribute infant formula as it can be wrongly interpreted as them promoting the products, even if they are doing so to meet the needs of the targeted population[47].

Case Study 3: Israel-Hezbollah conflict, 2006: Lebanon

1. During the Lebanon conflict, an NGO violated the IYCF-E guidelines by distributing 1,500 'baby kits', including infant formula and bottles, directly to displaced households, as well as to hospitals and local municipalities. Even after the conflict, the same NGO distributed 'village kits' containing infant formula (25 boxes containing 24 cans each) and baby food (80 units) along with other items to each village municipality[10, 47].
2. Another violation was the donation of infant formula to the healthcare facilities by an international NGO during the Lebanon conflict[10].
3. Moreover, one local NGO distributed the infant formula amongst the displaced population, without clearly specifying on the tins that it should only be used on the advice of a health worker thus violating the labelling requirements, and promoting infant formula use[10].
4. Another violation occurred when single tins/samples of infant formula were distributed to mothers by health workers, without the undertaking that the infant formula supplies would continue for as long as the concerned infant needs them[10].

Case Study 4: Israel-Hezbollah conflict, 2006: Lebanon

1. The formulas received by Lebanese government organizations and NGOs had labels written in English and/or Greek, instead of the local language, Arabic[10, 47].
2. One local NGO distributed infant formula, advertising and promoting artificial feeding, which was in violation of labelling requirements[10, 47].

414

Complementary Feeding

Twenty guidelines reported on complementary feeding in infants 6-24 months of age[2, 33, 42, 45, 46, 49, 50, 52, 55, 56, 58-60, 62-66]. For normal growth and development of infants (>6 months), easily digestible complementary foods should be started along with continuation of breast milk. Discussions should be conducted in groups, and mothers should be encouraged to increase the frequency and variety of complementary food with the growing age of child to meet their nutritional demands. Mothers should be encouraged to use locally produced, inexpensive complementary foods. However, in emergency situations, micro-nutrient fortified blended foods, Ready-to-Use Supplementary Foods (RUSFs), Lipid based Nutrient Supplements (LNS) or ferrous sulphate iron solution (iron drops) can be used depending on nutritional situation. Relief workers should ensure that complementary food products are labelled in local language with instructions on preparation and do not have the images of bottle feeding on them, and donations of complementary foods, baby teas or juices should be refused. For children over the age of 12 months, it is recommended that they should eat the same food as older children. If safe complementary foods are not available, mothers should be advised to continue breastfeeding.

Caring for Caregivers and Protecting Children

Thirteen guidelines reported on caring for caregivers during emergencies[2, 34, 45, 46, 53, 59, 61-63, 65-68] and nine reported on protecting children[2, 34, 46, 49, 50, 53, 58-60, 62, 63, 65, 68]. Guidelines recommend provision of psychological support and empowering mothers during crisis and advising them to continue breastfeeding (via milk expression). It should be ensured that infants are screened for childhood illnesses and mothers of artificially-fed infants have access to cleaning facilities for washing utensils for safe preparation of BMS as artificially-fed infants are known to be at a greater risk of malnutrition, diarrhea and chest infections. If an infant is ill with reduced appetite, mothers should continue breastfeeding with smaller amounts and increased frequency. Relief staff should prioritize infants for re-lactation, re-establishment of exclusive breastfeeding and BMS provision and associated support services.

Malnutrition

Nine guidelines reported on management of malnutrition in infants during emergencies[2, 45, 53, 55, 56, 59, 61, 66, 68]. Guidelines recommend continuous monitoring of nutritional status of mothers, infants and young children with the purpose of identifying, assessing, preventing and treating malnourished mothers and children. Malnutrition treatment and prevention programs should incorporate and prioritize IYCF in their agenda as the risk can be decreased with optimal IYCF practices. Education should be provided to community support networks on the prevention and treatment of acute malnutrition and cash/voucher programs should be introduced. For prevention of malnutrition, micronutrients should be distributed to all pregnant and lactating women (PLWs) in form of powders and tablets. It is recommended to identify malnourished children through regular monitoring and they should be referred and admitted along with their mothers to a nutritional rehabilitation program in case of severe malnourishment. In crisis situations, supplementary feeding should be the primary strategy for prevention and treatment of MAM and pre-formulated therapeutic milk products or dried skimmed milk (DSM) can be used to treat SAM.

The Acute Phase of Emergencies

Twelve guidelines reported on interventions to be undertaken during the acute phase of emergencies[1, 2, 42, 45, 46, 52, 56, 59, 62, 63, 65, 68]. In case of an emergency, interventions should start immediately with focus on capacity building to improve IYCF-E practices, supporting caregivers and catering to nutritional needs of children in order to minimize the negative impact of emergency. In places with prior high infant formula use, appropriate interventions should be undertaken by relevant organizations to increase prevalence of appropriate IYCF-E practices and increase the rate of EBF and measures should be taken to monitor BMS donations and distribution during early phases of emergencies. Moreover, mothers should be educated and encouraged to breastfeed every 2-3 hours at breastfeeding stations scattered across refugee sites. If mothers are experiencing difficulty in breastfeeding, chlorpromazine can be used to stimulate milk production and wet-nursing and milk banks can also be used as an alternative to BMS.

Assessment, Intervention and Monitoring

Fifteen guidelines mentioned assessment, intervention and monitoring during emergencies[1, 2, 33, 45, 46, 52, 53, 58-62, 65, 66, 68]. There should be regular systematic monitoring to track BMS distribution and careful monitoring of optimal feeding and nutritional status of infants and young children. It is recommended to conduct weight monitoring, assess intake, urination frequency, and activity level for those receiving BMS. Healthcare staff should use qualitative as well as quantitative methods to gather data regarding pre-crisis practices, demographics, morbidity, mortality, malnutrition and current IYCF practices.

Breastfeeding and HIV

Eleven guidelines reported on breastfeeding in HIV situations[2, 46, 48, 56-59, 61, 64, 68]. Guidelines recommend that appropriate measures should be undertaken to prevent mother-to-child transmission of HIV, and improve child survival from HIV. If the mother's HIV status is negative or unknown (or HIV testing is not available), she should be advised to continue age appropriate breastfeeding and replacement feeding should only be supported if is acceptable, feasible, affordable, sustainable and safe (meets the AFASS criteria). For sustainable access to medical care, HIV positive mothers should be provided with antiretroviral treatment (ART) and, in case of unavailability of ARTs, breastfeeding should be continued.

If HIV-positive mothers choose not to breastfeed the infant, appropriate BMS should be provided along with counselling on the risks of mixed feeding and artificial feeding. Wet nursing should also be considered in cases of HIV-positive mothers and wet nurse should be counseled on prevention of disease transmission. IYCF-E staff should make supportive arrangements for HIV-positive mothers to build confidence, reduce isolation, encourage age appropriate feeding and educate the family members to provide full support to the mothers and conduct regular follow ups. Furthermore, activities related to Prevention/Elimination of Mother-to-Child Transmission (PMTCT) should be regularly done as part of nutritional interventions and the measures taken by ICYF-E staff should be sensitive.

DISCUSSION

This review included a total of 56 primary studies and grey literature, covering IYCF practices, programs and guidelines for the countries affected by armed conflict. The review shows that the IYCF practices are very low and sub-optimal in conflict settings, as only half of the children receive early initiation of breastfeeding and a quarter of children are exclusively breastfed. The other IYCF indicators are also not very encouraging, with high rates of bottle-feeding. The review also shows a high burden of childhood malnutrition and infectious diseases especially diarrhea in conflict settings leading to a rise in diarrhea specific mortality. Conflict has a direct impact of IYCF practices as during the conflict in Iraq, the EBF decreased from 40% in the first month to 6% in the fifth month of conflict[26] and prevalence of GAM increased from 16.1% to 19.6% within a year of conflict in South Sudan[18].

There are many reasons for low IYCF indicators including displacements, stress, maternal malnourishment, lack of awareness, and unavailability of trained healthcare professionals. The death of the male members within the family also poses an additional barrier, as apart from increased maternal mental stress, it also leads to additional maternal responsibilities, hence compromising the attention to children. The review suggests there are misconceptions in the community that malnourishment and stress amongst mothers leads to decrease in the quantity and quality of breast milk, hence children should be supported with BMS. Health workers also in some instances advocate and prescribe BMS due to their lack of awareness and knowledge and the unregulated marketing, provision and distribution of BMS further compromises the situation.

There is a need to enhance the capacity of the health workers and enhance communication with the community utilizing various channels, and though various programs have been initiated in these conflict settings[44], but unfortunately none of these has been formally evaluated to gauge the impact of these different approaches. The major evidence and recommendations are from the implementation guidelines which have been formulated by various agencies with experience in working in these contexts. First and foremost, the importance of IYCF should be underscored and it should be a top priority for improving health of children in conflict settings. There should be an early dissemination of policies to all concerned agencies and healthcare workers with a greater emphasis on improving the capacity of the health workers. All the women and mothers should be registered whether in camps, or IDPs or living in conflict zones with possible tracking of their movement.

Educational approaches should be drafted with specific messages to alleviate the context specific common misconceptions within the community. Various communications platforms could be utilized to spread these messages including women support groups, involving prominent community members, designing pictorials, brochures or videos. There should be designated places like feeding tents which could provide a personal space for women to feed their children, seek support from peers and these could also be avenues for skin to skin care for preterm and low birthweight infants. The provision of clean hygienic utensils and safe drinking water should also be ensured for preparing complementary feeds. The BFHI should be strengthened in the functioning health facilities. The mothers apart from access to healthcare, should also be provided with

For peer review only - http://bmjopen.bmj.com/site/about/guidelines.xhtml

lactation and psychological support. The other factors include high turnover rate of health workers, lack of funds, poor multi-sectoral coordination, poor monitoring and evaluation system, more focus on malnutrition treatment than prevention, strengthened marketing efforts of BMS by industries, and poor capacity at community level.

The additional approaches like ‘wet nurse’ and ‘milk banks’ could be sought for only mothers who could not breastfeed and after confirmation from specialists, but BMS should be the last resort[3]. There should be stringent regulatory checks on BMS and nipples and pacifiers with no marketing and unchecked supply of formula milk[3, 71]. All of the commodities in a conflict setting should flow through a common medium and a single designated agency should be responsible for controlling donations and distributing appropriately labeled infant formula, and any violation to these should be reported with timely action taken. In cases of acute emergency, RUSF and LNS could be distributed.

A similar study by Seal 1999[69] and Seal 2001[12] reported on constraints in implementation of guidelines and policy on IYCF. Another study, Prudhon 2018 analyzed 28 reviews on interventions to promote breastfeeding from LMICs in conflict and disaster settings[70] and it also reported a dearth of evidence on IYCF-E outcomes. One of the major strengths was that our review was comprehensive looking at various areas of IYCF. The limitations of our review include restricted access to studies conducted by various NGOs/ agencies as most of them don’t report and we included articles published in English and some of the included studies were of poor quality which failed to report on study context (e.g. year and scale of conflict/surveys), outcomes, and on process indicators. We found only three studies from conflict-affected countries in Asian region. Furthermore, most of the included studies did not comprehensively evaluate the program impact so analysis couldn’t be performed. Therefore, there is a critical need of further research on the process of implementation, effectiveness of IYCF interventions and cost effectiveness of these interventions in conflict settings.

To ensure effective scale-up of interventions for promotion of IYCF, there should be evidence-based advocacy, need-based assessments, committed funding, political will, and structured, rigorous monitoring and evaluation system and multi-sectoral approach emphasizing on IYCF together with immunization, infectious diseases management and growth monitoring.

LIST OF ABBREVIATIONS

ACF	Action contre La Faim
AFASS	Acceptable, Feasible, Affordable, Sustainable and Safe
ARI	Acute Respiratory Infection
ART	Antiretroviral Treatment
BFHI	Baby Friendly Hospital Initiative
BMS	Breast-Milk Substitutes
CAR	Central African Republic
CMR	Crude Mortality Rate
DEWS	Disease Early Warning System
DRC	Democratic Republic of Congo
DSM	Dried Skimmed Milk
EBF	Exclusive Breastfeeding
ENN	Emergency Nutrition Network
GAM	Global Acute Malnutrition
GINA	Global Database on the Implementation of Nutrition Action
HIC	High Income Countries
HIV	Human Immunodeficiency Virus
IBFAN	The International Baby Food Action Network
IDPs	Internally Displaced Persons
IMR	Infant Mortality Rate
IYCF	Infant and Young Child Feeding
IYCF-E	Infant and Young Child Feeding in Emergencies
KAP	Knowledge, Attitude and Practices
LMIC	Low- and Middle-Income Countries
LNGO	Local Non-Governmental Organization
LNS	Lipid-based nutrient supplements
MAM	Moderate Acute Malnutrition
MESH	Medical Subject Headings
MICS	Multiple Indicator Cluster Survey
MR	Mortality Rate
MtMSG	Mother-to-Mother Support Groups
WB	World Bank
MUAC	Mean Upper Arm Circumference
NGO	Non-Governmental Organization
INGOs	International Non-Governmental Organization
PLW	Pregnant and Lactating Women
PMTCT	Prevention/Elimination of Mother-to-Child Transmission
RUSF	Ready-to-Use Supplementary Foods
SAM	Severe Acute Malnutrition
SMART	Standardized Monitoring and Assessment of Relief and Transition
SOPs	Standard Operating Procedures
TBA	Traditional Birth Attendants

For peer review only

U5MR	Under Five Mortality Rate
UN	United Nations
UNHCR	The United Nations High Commissioner for Refugees
UNICEF	United Nations International Children's Emergency Fund
WHA	World Health Assembly
WHO	World Health Organization

For peer review only

DECLARATIONS

Ethical Approval

We obtained ethical approval of this study from the Ethical Review Committee of Aga Khan University, Karachi and the National Bioethics Committee, Pakistan.

Consent for Publication

Not Applicable

Acknowledgements

None

Competing Interests

All the authors declare no conflict of interest. The corresponding author is the overall guarantor and affirms that the manuscript is an honest, accurate, and transparent account of the study.

Data Sharing

The datasets used for analysis in this study are available from the corresponding author on reasonable request. As this is a systematic review, dissemination to the groups is not applicable.

Funding

The Aga Khan University received a grant from the Family Larsson-Rosenquist Foundation. The funders had no role in the findings and writing of the manuscript.

Authors' Contributions

ZAB and JKD conceived the idea of the review. ZAP developed the search strategy; AR, ZAP and FAS conducted the search and data extraction with specific inputs from JKD. AR, ZAP and JKD developed the first draft of the paper. ZAB and JKD reviewed and finalized the final manuscript.

For peer review only

REFERENCES

1. Borrel A, Taylor A, McGrath M, Seal A, Hormann E, Phelps L, et al. From policy to practice: challenges in infant feeding in emergencies during the Balkan crisis. Disasters. 2001;25(2):149-63.
2. ACF-International. Infant and Young Child Feeding in Emergencies. 2015 December 2015.
3. Campbell S. Global challenges in protecting and promoting breastfeeding. Prim Health Care. 2008;18(1):41-48.
4. Sinha B, Chowdhury R, Sankar MJ, Martines J, Taneja S, Mazumder S, et al. Interventions to improve breastfeeding outcomes: a systematic review and meta-analysis. Acta paediatrica (Oslo, Norway : 1992). 2015;104(467):114-34.
5. Victora CG, Bahl R, Barros AJD, França GVA, Horton S, Krasevec J, et al. Breastfeeding in the 21st century: epidemiology, mechanisms, and lifelong effect. The Lancet. 2016;387(10017):475-90.
6. O'Connor ME, Burkle FM, Jr., Olness K. Infant feeding practices in complex emergencies: a case study approach. Prehosp Disaster Med. 2001;16(4):231-8.
7. Essential nutrition actions: mainstreaming nutrition through the life-course. Geneva: World Health Organization; 2019. Licence: CC BY-NC-SA 3.0 IGO.
8. Davanzo R. Newborns in adverse conditions: issues, challenges, and interventions. J Midwifery Womens Health 2004;49(4 Suppl 1):29-35.
9. Alsamman S. Managing infant and young child feeding in refugee camps in Jordan. ENN [Internet]. 2014 Nov [cited 2019 Apr 17]; (48):85. Available form: https://www.ennonline.net/fex/48/managinginfant.
10. Maclaine A. Infant feeding in emergencies: experiences from Lebanon. 2007. Contract No.: 37.
11. Bustreo F, Genovese E, Omobono E, Axelsson H, Bannon I. Improving child health in post-conflict countries: Can the World Bank contribute. The World Bank. 2005.
12. Seal A, Taylor A, Gostelow L, McGrath M. Review of policies and guidelines on infant feeding in emergencies: common ground and gaps. Disasters. 2001;25(2):136-48.
13. Ploughshares P. Armed Conflict 2016 [Available from: http://ploughshares.ca/armed-conflict/defining-armed-conflict/.
14. Bank W. New country classifications by income level: 2018-2019.; 2018 2018.
15. Clarivate. Endnote. In: Group WoS, editor. 7.8 ed2019.
16. Dozio E, Peyre L, Morel SO, Bizouerne C, Black. Integrated psychosocial and food security approach in an emergency context: Central African Republic. Intervention: Journal of Mental Health and Psychosocial Support in Conflict Affected Areas. 2016;14(3):257-71.
17. Fander G, Frega M. Responding to nutrition gaps in Jordan in the Syrian Refugee Crisis: Infant and Young Child Feeding education and malnutrition treatment. ENN [Internet]. 2014 Nov [cited 2019 Apr 17]; (48): 82. Available from: https://www.ennonline.net/fex/48/responding.
18. Ndungu P, Tanaka J. Using care groups in emergencies in South Sudan. ENN [Internet]. 2017 Feb [cited 2019 Apr 17]; (54): 95. Available from: https://www.ennonline.net/fex/54/emergenciesinsouthsudan.
19. Aakre I, Lilleengen AM, Aarsand ML, Strand TA, Barikmo I, Henjum S. Infant feeding practices in the Saharawi refugee camps Algeria, a cross-sectional study among children from birth to six months of age. International breastfeeding journal. 2016;12(1):8.

For peer review only

20. Jakobsen M, Sodemann M, Nylen G, Bale C, Nielsen J, Lisse I, et al. Breastfeeding status as a predictor of mortality among refugee children in an emergency situation in Guinea-Bissau. Trop Med Int Health. 2003;8(11):992-6.
21. Robertson A, Fronczak N, Jaganjac N, Hailey P, Copeland P, Duprat M. Nutrition and immunization survey of Bosnian women and children during 1993. Int J Epidemiol. 1995;24(6):1163-70.
22. Summers A, Bilukha OO. Suboptimal infant and young child feeding practices among internally displaced persons during conflict in eastern Ukraine. Public Health Nutr. 2018;21(5):917-26.
23. Theurich MA, Grote V. Are commercial complementary food distributions to refugees and migrants in Europe conforming to international policies and guidelines on infant and young child feeding in emergencies? Journal of Human Lactation. 2017;33(3):573-7.
24. Andersson N, Paredes-Solis S, Legorreta-Soberanis J, Cockcroft A, Sherr L. Breast-feeding in a complex emergency: four linked cross-sectional studies during the Bosnian conflict. Public health nutrition. 2010;13(12):2097-104.
25. Šumanović-Glamuzina D. Breastfeeding practice in Bosnia and Herzegovina. Journal of Pediatric and Neonatal Individualized Medicine (JPNIM). 2013;2(1):55-62.
26. Diwakar V, Malcolm M, Naufal G. Violent Conflict and Breastfeeding: The Case of Iraq. 2017.
27. Darjani P, Berbari LS. Infant and young child feeding support in Lebanon: strengthening the national system. ENN [Internet]. 2014 Nov [cited 2019 Apr 17]; (48):20. Available from: https://www.ennonline.net/fex/48/infant.
28. Seguin J. Challenges of IYCF and psychosocial support in Lebanon. ENN [Internet]. 2014 Nov [cited 2019 Apr 17]; (48): 24. Available from: https://www.ennonline.net/fex/48/challenges.
29. Khudari WHO response to malnutrition in Syria: a focus on surveillance, case detection and clinical management. ENN [Internet]. 2014 Nov [cited 2019 Apr 17]; (48): 118. Available from: https://www.ennonline.net/fex/48/whosyria.
30. UNHCR. UNHCR Nutrition Survey among Afghan Refugees Residing in Afghan Refugee Villages of Pakistan. 2014.
31. Sallam AK, Albably K, Zvandaziva C, Singh K. Community engagement through local leadership: Increasing access to nutrition services in a conflict setting in Yemen. ENN [Internet]. 2018 Jan [cited 2019 Apr 17]; (9):10. Available from: https://www.ennonline.net/nex/9nutserviceaccessyemen.
32. Bile KM, Hafeez A, Kazi GN, Southall D. Protecting the right to health of internally displaced mothers and children: the imperative of inter-cluster coordination for translating best practices into effective participatory action. Eastern Mediterranean Health Journal. 2011;17(12):981-9.
33. Amelia Reese Masterson, Shiromi M. Perera, Moghames P. Camp-based Barrier Analysis of Early Initiation of Breastfeeding, Iron-rich Food Consumption, and Early Antenatal Care Seeking Behaviors of Syrian Refugees in Azraq Camp, Jordan. International Medical Corps USAID; 2016.
34. Prudhon C. Assessment of Infant and Young Child Feeding Practices among Refugees on Lesvos Island, Greece 2016.
35. Hunger AA. Infant Feeding and Weaning Report 2000.
36. Mary S Lung'aho, Stone-Jiménez M. Mother-to-Mother Support Groups in the Dadaab Refugee Camps.

37. Children St. Emergency WASH & Nutrition for conflict affected people in Yemen – Taiz governorate. 2017.
38. WHO. Indicators for assessing infant and young child feeding practices. 2008.
39. Mukunya D, Tumwine JK, Nankabirwa V, Ndeezi G, Odongo I, Tumuhamye J, et al. Factors associated with delayed initiation of breastfeeding: a survey in Northern Uganda. Glob Health Action. 2017;10(1):1410975.
40. Holland R. Frontline: Support for breastfeeding in crisis. Lancet. 2017;390(10097):834.
41. Renata Jelušić R. Support for breastfeeding and young child feeding in emergencies. Experiences gained during the migrant and refugee crisis in Croatia in 2015 and 2016. 2017.
42. Program WIEPoB. Infant and Young Child Feeding in Emergency Situations. 2005.
43. Saraswati Khalsa EH, Eric Sarriot. Systems Effects Of Save The Children Emergency Health & Projects Sudan. 2019.
44. USAID Core Group members. Humanitarian Development Nexus Stakeholder and Literature Analysis. 2019.
45. WHO. Guiding principles for feeding infants and young children during emergencies. 2004.
46. Group IC. Infant and Young Child Feeding in Emergencies Operational Guidance for Emergency Relief Staff and Programme Managers. 2017 October 2017.
47. Focus I. The Code and infant feeding in emergencies. 2009 May 2009.
48. Machel G. Impact of Armed Conflict on Children. United Nations; 1996 26 August 1996.
49. Association ILC. Breastfeeding: A Vital Emergency Response. Are You Ready? . 2009.
50. Association ILC. Breastfeeding: A Vital Emergency Response. Are You Ready? For relief workers. 2009.
51. WHO. International Code of Marketing of Breast-milk Substitutes. Geneva; 1981.
52. OXFAM. Feeding in Emergencies For Infants Under Six Months: Practical Guidelines 1996.
53. WHO, UNICEF, LINKAGES, IBFAN, ENN. Infant feeding in emergencies Module 1 (for emergency relief staff). 2001 November 2001.
54. WHO. WHA RESOLUTION 47.5- Infant and young child nutrition. 1994.
55. Joint UNICEF U, WFP, WHO Statement Policy on infant feeding in the Balkan Region.
56. UNHCR. UNHCR Policy Related To The Acceptance, Distribution And Use Of Milk Products In Refugee Settings 2006.
57. UNHCR. Guidance on Infant feeding and HIV in the context of refugees and displaced populations. 2008 April 2008.
58. UNHCR. Infant and young child feeding practices- Standard Operating Procedures for the Handling of Breastmilk Substitutes (BMS) in Refugee Situations for children 0-23 months. 2015 August 2015.
59. UNHCR, children St. Infant and Young Child Feeding in Refugee Situations: A Multi-Sectoral Framework for Action. 2018 May, 2018.
60. ACF-International. Baby friendly spaces. Holistic approach for Pregnant, Lactating Women anf their very young children in Emergency. 2014 December 2014.
61. ENN, IBFAN-GIFA, Fondation Terre des hommes, Action Contre la Faim, CARE USA, LINKAGES, et al. Infant feeding in emergencies (for health and nutrition workers in emergency situations). 2007.
62. Refugees UHCf. Interim Operational Considerations for the feeding support of Infants and Young Children under 2 years of age in refugee and migrant transit settings in Europe. 2015.

For peer review only

63. Save the Children U, Food Security and Nutrition Network. IYCF-E Toolkit: Summary of Operational Guidance on IYCF-E Key Points and WHA Resolution. 2007.
64. Save the Children U, Food Security and Nutrition Network. IYCF-E Toolkit: Summary of Feeding the Non-breastfed child 6- 24 months of Age 2007.
65. Pediatrics AAo. Infant feeding in disasters and emergencies. 2015.
66. UNICEF. UNICEF Guidance on the provision and use of breastmilk substitutes in humanitarian settings. 2018 June 2018.
67. UNHCR. HOW TO GUIDE reproductive health in refugee setting (Strengthening Safe Motherhood Services). 1999 November 1998.
68. ENN. Infant Feeding in Emergencies: Policy, Strategy and Practice. 1999 May 1999.
69. McGrath M, Seal A, Taylor A, Gostelow L. Meeting the nutritional needs of infants during emergencies: Recent experiences and dilemmas: Report of an international workshop, Institute of Child Health, London, November 1999. 2000.
70. Prudhon C, Benelli P, Maclaine A, Harrigan P, Frize J. Informing infant and young child feeding programming in humanitarian emergencies: An evidence map of reviews including low and middle income countries. Matern Child Nutr. 2018;14(1).
71. Aguayo VM, Ross JS, Kanon S, Ouedraogo AN (2003) Monitoring compliance with the International Code of Marketing of Breastmilk Substitutes in west Africa: multisite cross sectional survey in Togo and Burkina Faso. British Medical Journal. 326, 127.

For peer review only

Figures and Tables

Table 1: IYCF practices in conflict settings
Table 2: Programs/ Interventions to Promote Optimal IYCF Practices
Figure 1: Conceptual Framework
Figure 2: Search Flow Diagram
Figure 3: Barriers to optimal breastfeeding practices
Figure 4: Implementation Guidelines

For peer review only

TABLES

Table 1: IYCF practices in conflict settings

	Indicators	Target Population: Country	Setting	Estimates Median (range)	Sample size Median (range)	Scale
**Core Indicators**	Early Initiation of Breastfeeding (n=10)[16, 18, 19, 22, 25, 28, 30, 33, 36, 37]	- IDPs: South Sudan, Ukraine<br>- Refugees: Algeria, Jordan, Kenya, Lebanon, Pakistan<br>- Not Specified: Bosnia-Herzegovina, CAR, Yemen	- Camp: Algeria, Jordan, Kenya, Lebanon<br>- Community: Pakistan, South Sudan, Ukraine, Yemen	51% (31.3% to 85%)	357 (111-1368)	- Camps: Algeria, Jordan, Kenya<br>- Village: Pakistan<br>- District: Yemen<br>- National: CAR
	Exclusive breastfeeding under 6 months (n=16)[11, 18, 19, 21-23, 25-28, 30, 33-37]	- IDPs: Bosnia-Herzegovina, Ukraine, Sierra Leone, South Sudan<br>- Refugees: Algeria, Greece, Jordan, Kenya, Lebanon, Pakistan<br>- Not Specified: Afghanistan, Bosnia-Herzegovina, Iraq, Kosovo, Lebanon, Syria, Yemen	- Camp: Algeria, Kenya, Lebanon<br>- Community: Bosnia-Herzegovina, Iraq, Kosovo, Pakistan, South Sudan, Ukraine, Yemen<br>- Community and Healthcare Facility: Jordan	25% (5.5% to 77.1%)	432 (58-250,000)	- Camps: Algeria, Jordan, Kenya<br>- Village: Pakistan<br>- District: Yemen<br>- National: Kosovo
	Continued Breastfeeding at one years (n=7)[22, 25, 28, 30, 34, 36, 37]	- IDPs: Ukraine<br>- Refugees: Greece, Kenya, Lebanon, Pakistan<br>- Not Specified: Bosnia-Herzegovina, Yemen	- Camp: Kenya, Lebanon<br>- Community: Pakistan, Ukraine, Yemen	57.20% (8.5% to 78.2%)	326 (38-1368)	- Camp: Kenya<br>- Village: Pakistan<br>- District: Yemen
	Introduction of Solid, Semi Solid or Soft Food (n=6) [19, 22, 25, 30, 34 36]	- IDPs: Ukraine<br>- Refugees: Algeria, Greece, Kenya, Pakistan<br>- Not Specified: Bosnia-Herzegovina,	- Camp: Algeria, Kenya<br>- Community: Pakistan, Ukraine	71.05% (40.7% to 98.6%)	477 (111-1368)	- Camps: Algeria, Kenya<br>- Village: Pakistan
	Minimum Dietary Diversity (n=5)[16, 18, 30, 34, 37]	- IDPs: South Sudan<br>- Refugees: Greece, Pakistan<br>- Not Specified: CAR, Yemen	- Community: Pakistan, South Sudan Yemen	60.25% (9.2% to 79.4%)	309 (148-1055)	- Village: Pakistan<br>- District: Yemen<br>- National: CAR
	Minimum Meal Frequency (n=6)[16, 18, 22, 30, 34, 37]	- IDPs: South Sudan, Ukraine<br>- Refugees: Greece, Pakistan<br>- Not Specified: CAR, Yemen	- Community: Pakistan, South Sudan, Ukraine, Yemen	58% (58% to 97.6%)	432 (148-1055)	- Village: Pakistan<br>- District: Yemen<br>- National: CAR

For peer review only

	Minimum Acceptable Diet (n=5)[16, 18, 30, 34, 37]	- IDPs: South Sudan<br>- Refugees: Greece, Pakistan<br>- Not Specified: CAR, Yemen	- Community: Pakistan, South Sudan, Yemen	24.95% (30.5% to 33%)	309 (148-1055)	- Village: Pakistan<br>- District: Yemen<br>- National: CAR
	Consumption of Iron Rich and Iron Fortified Food (n=2)[22, 33]	- IDPs: Ukraine<br>- Refugees: Jordan	- Camp: Jordan<br>- Community: Ukraine	51.61% (29% to 84.7%)	379 (281-477)	- Camp: Jordan
**Optional Indicators**	Children ever breastfed (n=5 [22, 24, 30, 34, 35]	- All (refugees, displaced and not displaced): Bosnia-Herzegovina<br>- IDPs: Ukraine<br>- Refugees: Greece, Macedonia, Pakistan	- Camp: Macedonia<br>- Community: Bosnia-Herzegovina, Pakistan, Ukraine	92% (62.8% to 98.4%)	766 (148-1123)	- Camp: Macedonia<br>- Village: Pakistan<br>- National: Bosnia-Herzegovina
	Continued Breastfeeding at two years (n=6)[22, 25, 26, 28, 30, 34]	- IDPs: Ukraine<br>- Refugees: Lebanon, Pakistan, Greece<br>- Not Specified: Bosnia-Herzegovina, Iraq	- Camp: Lebanon<br>- Community: Iraq, Pakistan, Ukraine	29% (9% to 66%)	477 (148- 55194)	- Village: Pakistan<br>- National: Iraq
	Age appropriate breastfeeding (n=3)[25, 30, 34]	- Refugees: Greece, Pakistan<br>- Not Specified: Bosnia-Herzegovina	- Community: Pakistan	43.20% (19.5% to 77.8%)	602 (148-1055)	- Village: Pakistan
	Predominant breastfeeding under 6 months (n=5)[19, 25, 30, 34, 35]	- Refugees: Algeria, Greece, Pakistan<br>- Not Specified: Bosnia-Herzegovina, Kosovo	- Camp: Algeria<br>- Community: Kosovo, Pakistan	31.30% (7.1% to 77.30%)	176 (111-1055)	- Village: Pakistan
	Duration of breastfeeding (n=2)[20, 24]	- All (refugees, displaced and not displaced): Bosnia-Herzegovina<br>- IDPs: Guinea-Bissau	- Community: Bosnia-Herzegovina, Guinea-Bissau	22.7 months	2149 (1741-2556)	- City: Guinea-Bissau<br>- National: Bosnia-Herzegovina
	Bottle feeding (n=3)[22, 28, 30]	- IDPs: Ukraine<br>- Refugees: Lebanon, Pakistan	- Camp: Lebanon<br>- Community: Pakistan, Ukraine	58.30% (31.8% to 71.4%)	477 (174-1055)	- Village: Pakistan
	Milk feeding frequency of non-breastfed children (n=1)[34]	- Refugees: Greece	- Not Stated	33.90%	148	- Not Stated
Malnutrition	Underweight (n=4)[11, 19, 30, 37]	- IDPs: Afghanistan, Sierra Leone<br>- Refugees: Algeria, Pakistan<br>- Not Specified: East Timor, Yemen	- Camp: Algeria<br>- Community: Pakistan, Yemen	33.10% (12.01% to 48%)	1055 (111-250,000)	- Camp: Algeria<br>- Village: Pakistan<br>- District: Yemen
	Acute Malnutrition (n=3)[11, 22, 32]	- All ((IDPs, refugees and residents): Southern Somalia<br>- IDPs: Ukraine, Pakistan	- Camp: Southern Somalia, Ukraine<br>- Camps, hospitals and mobile clinics: Pakistan	30.10% (0.5% to 81%)	80,000 (477-3,000,000)	- Camps and Healthcare facilities: Pakistan

For peer review only

	GAM (n=7)[9, 16, 18, 30, 31, 36, 37]	- IDPs: South Sudan<br>- Refugees: Jordan, Kenya, Pakistan<br>- Not Specified: CAR, Yemen	- Camp: Jordan, Kenya<br>- Community: Pakistan, South Sudan, Yemen	9.95% (2.6% to 25.1%)	498 (208-1368)	- Camp: Jordan, Kenya<br>- Village: Pakistan, Yemen<br>- District: Yemen
	MAM (n=9)[11, 17-19, 21, 22, 30, 37, 43]<br>(Z- score <-2 to <-3 SD)	- IDPs: Afghanistan, Bosnia-Herzegovina, Sierra Leone, South Sudan, Sudan, Ukraine<br>- Refugees: Algeria, Jordan, Pakistan<br>- Not Specified: East Timor, Yemen	- Camp: Algeria, Ukraine<br>- Community: Bosnia- Herzegovina, Jordan, Pakistan, South Sudan, Sudan, Yemen	4.0% (0.27 to 25%)	563 (111-250,000)	- Camp: Algeria<br>- Village: Pakistan<br>- District: Yemen<br>- Governorates: Jordan
	SAM (n=8)[16-18, 30, 31, 36, 37, 43]<br>(Z- score <-3 SD)	- IDPs: South Sudan, Sudan<br>- Refugees: Jordan, Kenya, Pakistan<br>- Not Specified: CAR, Yemen	- Camp: Kenya<br>- Community: Jordan, Pakistan, South Sudan, Sudan, Yemen	1.50% (0.15% to 5.3%)	809 (208-46,383)	- Camp: Kenya<br>- Village: Pakistan, Yemen<br>- District: Yemen<br>- Governorates: Jordan
Chronic Malnutrition	Stunting (n=7)[11, 16, 18, 19, 30, 31, 37]	- IDPs: Afghanistan, Sierra Leone, South Sudan<br>- Refugees: Algeria, Pakistan<br>- Not Specified: East Timor, CAR, Yemen	- Camp: Algeria<br>- Community: Pakistan, South Sudan, Yemen	38.60% (13.6% to 53%)	432 (111-1055)	- Camp: Algeria<br>- Village: Pakistan, Yemen<br>- District: Yemen
	Overweight (n=1)[30]	- Refugees: Pakistan	- Community	18.1%	1055	- Village
	Anemia (n=1)[30]	- Refugees: Pakistan	- Community	23.2%	1055	- Village
Other Indicators	Diarrhea Prevalence (n=3)[11, 32, 37]	- IDPs: Guatemala, Pakistan<br>- Not Specified: Yemen	- Camps, hospital and mobile clinics: Pakistan<br>- Community: Yemen	30% (28.9% -73.8%)	303 (42-563)	- Healthcare facilities: Pakistan<br>- District: Yemen
	Mortality due to Diarrhea (n=3)[11, 21, 30]	- IDPs: Bosnia-Herzegovina, Somalia<br>- Refugees: Iraq, Nepal, Pakistan, Uganda, Zaire<br>- Residents: Eastern DRC	- Camps: Southern Somalia<br>- Community: Bosnia-Herzegovina, Pakistan	39% (22.3% to 87%)	100, 000 (1055-3,000,000)	- Village: Pakistan

For peer review only

Table 2: Programs/ Interventions to Promote Optimal IYCF Practices

Program	Target Population/ Countries	Setting	Health Workforce Involved	Program/ Intervention Details	Outcomes
Capacity Building and Program-Strengthening Projects for Health Workers (n=7)[9, 17, 27, 29, 36 ,40, 43]	- IDPs: Sudan, Syria<br>- Refugees: Jordan, Kenya, Lebanon<br>- Not Specified: Greece, Iraq	- Camps: Jordan<br>- Fixed and mobile clinics: Jordan, Lebanon	- Trained Physician (doctors, nurses)<br>- Paramedic Staff (midwives, TBAs)<br>- Community Workers (facility and community based IYCF counsellors, local reproductive health worker)	- Training sessions on IYCF<br>- Malnutrition screening and treatment<br>- Continuous follow up and co-ordination	**Coverage of Training**:<br>- In Lebanon and Syria: >190 doctors and health workers[27, 29]
Education and Awareness Activities for Mothers (n=7)[9, 16 17 , 27, 28, 32, 36]	-IDPs: Pakistan<br>-Refugees: Jordan, Kenya, Lebanon<br>-Not specified: CAR	- Camps: Jordan, Kenya, Lebanon, Pakistan<br>- Community: CAR, Lebanon<br>- Fixed and mobile clinics: Jordan, Pakistan<br>- Healthcare facility: Jordan Pakistan	- Trained Physician (lactation specialists)<br>- Community Workers (Nutrition officer, IYCF educator and a community mobiliser)	- Counselling sessions on optimal IYCF-E practices<br>- Educational materials and counselling cards distributed<br>- Sensitizations on childcare practices and cooking demonstrations given to PLW<br>- Distribution of hygiene and baby kits (soaps, blankets, baby spoons, and cups for children), and bangles for mothers	**Coverage of IYCF Counselling**<br>- In Jordan: 30-40 mothers counselled/day[9]; 4,690 PLWs and 919 mothers counselled in 10 months[17]<br>- In Lebanon: 10,000 mothers were counselled in one year[27]<br>- In CAR: 758/900 (84.2%) PLW participated[16]
Community Networking and Mobilization (n=8)[9, 17, 18, 27, 31, 36, 40, 43]	- IDPs: South Sudan, Sudan<br>- Refugees: Jordan, Kenya, Lebanon<br>- Not Specified: Greece, Iraq, Yemen	- Camps: Jordan, Kenya<br>- Community: Lebanon, South Sudan, Sudan, Yemen	- Community Workers (refugee mothers, community leaders, 'leader mothers' (mothers trained by promotors to teach neighbor women), and 'neighbor women' (chosen by community) as community mobilisers)	- Training of community mobilizers by IYCF counsellors, educators, 'promotors', program supervisors and IYCF coordinators on IYCF and the importance of exclusive breastfeeding, nutrition<br>- Dissemination of messages among mothers through household visits, demonstrations and information sharing within care groups<br>- Screening and referring malnourished mothers	**Coverage of Health Workers Training:**<br>- In South Sudan, 320 'leader mothers' trained IYCF[18]<br>**Coverage of IYCF Counselling**<br>- In Jordan: 4,977 PLWs and 31,485 caregivers in 10 months[17]<br>- In South Sudan: 320 neighborhood groups, reaching 3,832 women[18]<br>- In Yemen, 50-60% increase in awareness of mothers/caregivers on nutritious food along with an increase in utilization of local foods for preparing nutritious meals for infants[31]<br>**Mass Screening and SAM/MAM Treatment**<br>- In Yemen: 90% of children <2 years screened for SAM and MAM, 2,563 children were treated for SAM, reduction in number of cases of SAM and MAM children; zero cases of SAM (MUAC <115mm) in 13/68 model villages by the end of the project[31]<br>- In Sudan: 150,617 children screened for malnutrition[43]<br>- In Yemen: Reduction in bottle feeding to almost zero[31]
Mother Baby Friendly Spaces and Mother to Mother Support	- IDPs: South Sudan, Sudan	- Camps: Albania, Kenya, Lebanon<br>- Caravans: Jordan	- Trained Physician: Pediatrician, lactation consultant<br>- Community Workers: Relief workers, psychosocial workers, pediatrician,	- Construction of Mother-baby friendly spaces, caravans and mother baby center for counselling and 24-hour support	**Coverage of IYCF counselling in baby friendly spaces and mother-to- mother support groups:**<br>- In Jordan: 15,600 mothers in 18 months in Jordan[9]<br>- In Kenya: 581 facilitators trained in IYCF[36]

For peer review only

Groups (n=10)[9, 11, 16-18, 27, 28, 36, 41, 43]	- Refugees: Albania, Croatia, Jordan, Kenya, Lebanon<br>- Not Specified: CAR	- Community: South Sudan, Sudan<br>- Containers: Croatia<br>- Primary healthcare centers: Lebanon	lactation consultant, IYCF counsellors, and community mobilisers	- Formation of mother to mother/caregiver support groups<br>- Distribution of high energy biscuit, a bottle of water, and shawls for privacy<br>- Distribution of food vouchers to mothers for nutritional security and psychological support<br>- Screening of children for malnutrition	- In CAR: 199 mothers/ caregivers given psychosocial support[16]<br>**Other Outcomes:**<br>- In Jordan: 120-150 mothers/day visited baby friendly spaces in Jordan[9]<br>- In Jordan: 50 women attended support group gatherings[17]<br>- In Sudan: 14,272 infant/ mother pairs attended MtMSG[43]<br>- In Kenya: 713 MtMSG (2801 mothers/caregivers in MtMSG)[36]
Lactation Support Service (n=3)[11, 17, 27]	- Refugees: Albania, Jordan, Lebanon,	- Camps: Albania, Jordan<br>- Community: Lebanon	- Trained Physician (Lactation specialists, obstetrician/gynecologist)<br>- Paramedic Staff (midwives)	- Assist mothers for re-lactation and in breastfeeding difficulties (painful nursing, latching problems, low breast milk production, and on correct positioning for feeding)	**Coverage of counselling:**<br>- In Lebanon: 3,150 mothers in 6 months[27]
Baby Friendly Hospital Initiative (n=2) [25, 27]	- Refugees: Lebanon<br>- Not Specified: Bosnia-Herzegovina	- Camps: Lebanon	- Not Specified	- Labelling of maternity wards as "baby-friendly" to support breastfeeding<br>- Capacity building of health workforce<br>- Provision of tools and equipment to support breastfeeding, reducing use of BMS	In Bosnia-Herzegovina, from 1997-1999:<br>- Predominant breastfeeding increased from 64.3% to 77.3%<br>- Continued breastfeeding at 2 years increased from 8.5% to 40.7%
Breast Milk Substitutes (n=5)[9, 17, 27, 28, 40]	- Refugees: Jordan, Lebanon<br>- Not Specified: Greece, Iraq	- Camps: Greece, Iraq, Jordan and Lebanon	- Trained Physician (lactation specialists, and obstetrician/ gynecologist)<br>- Paramedic Staff (IYCF midwife)	- Training of healthcare staff and mothers on artificial feeding<br>- Counselling of mothers on importance of breastfeeding, appropriate use of infant formula and on adverse effects of artificial feeding on infant's health<br>- Monitor and control the distribution of infant formula- - Provision of BMS supplies and kits (cups and clean water) for safe preparation of infant formula	- In Lebanon: 50 infants were assisted with artificial feeding support[27]<br>- In Jordan: Seven mothers received artificial milk supplies[9]
**Key:** Breast Milk Substitutes (BMS); Internally Displaced Persons (IDPs); Infant and Young Child Feeding (IYCF); Infant and Young Child Feeding in Emergencies (IYCF-E); Traditional Birth Attendants (TBAs), Pregnant and Lactating Women (PLWs); Exclusive Breastfeeding (EBF); Central African Republic (CAR), Mother to mother support groups (MtMSG)					

For peer review only

For peer review only

FIGURES

Figure 1: Conceptual Framework

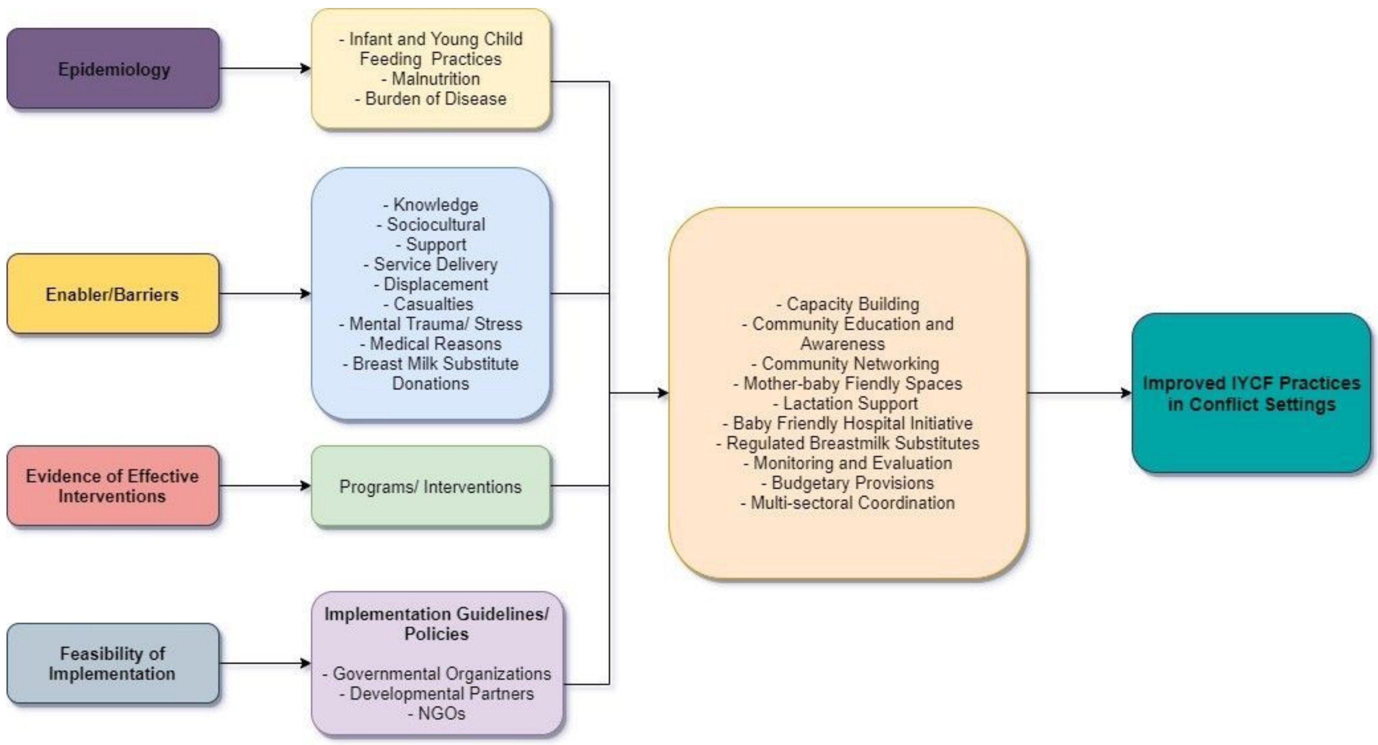

Figure 2: Search Flow Diagram

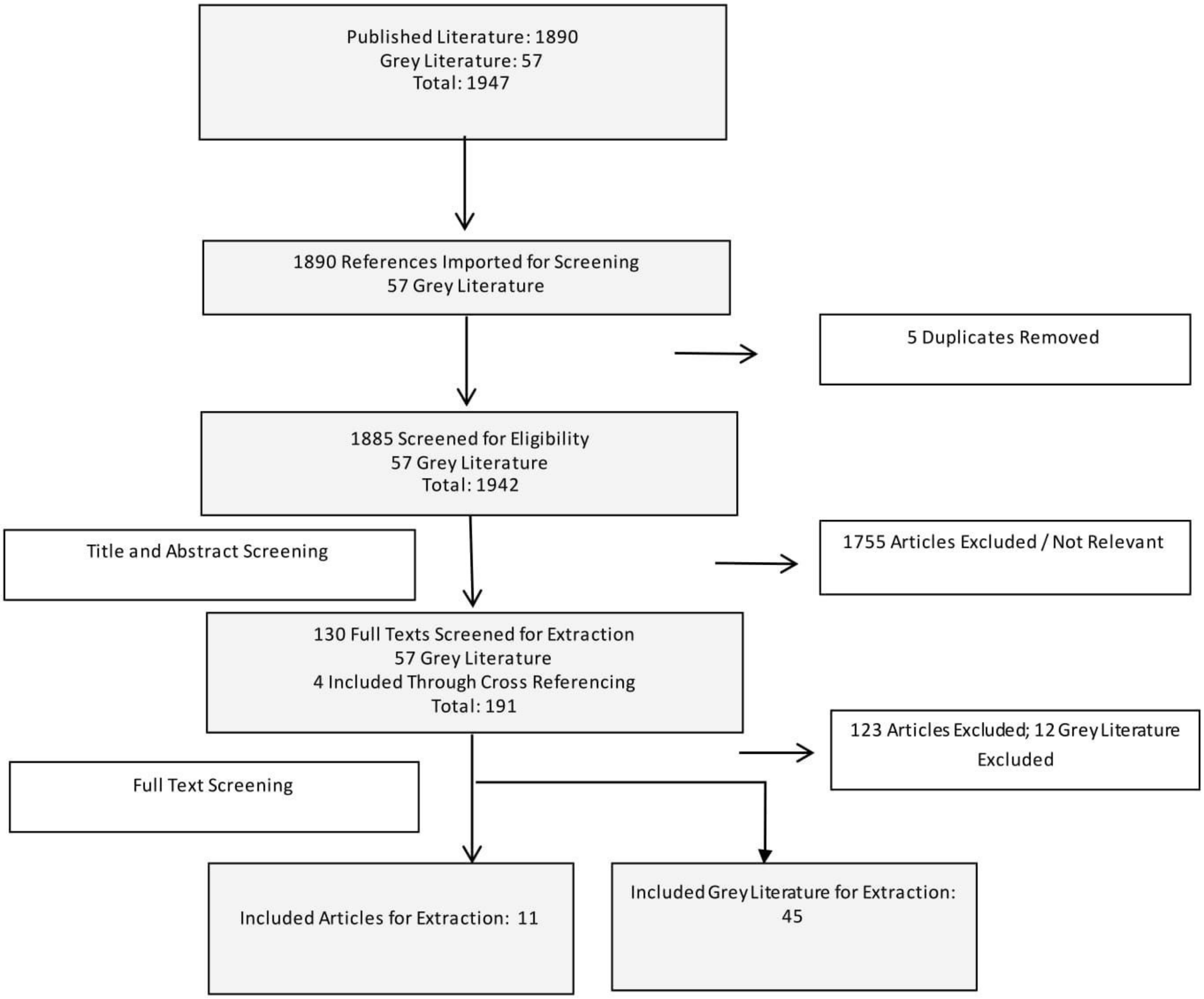

Figure 3: Barriers to optimal breastfeeding practices

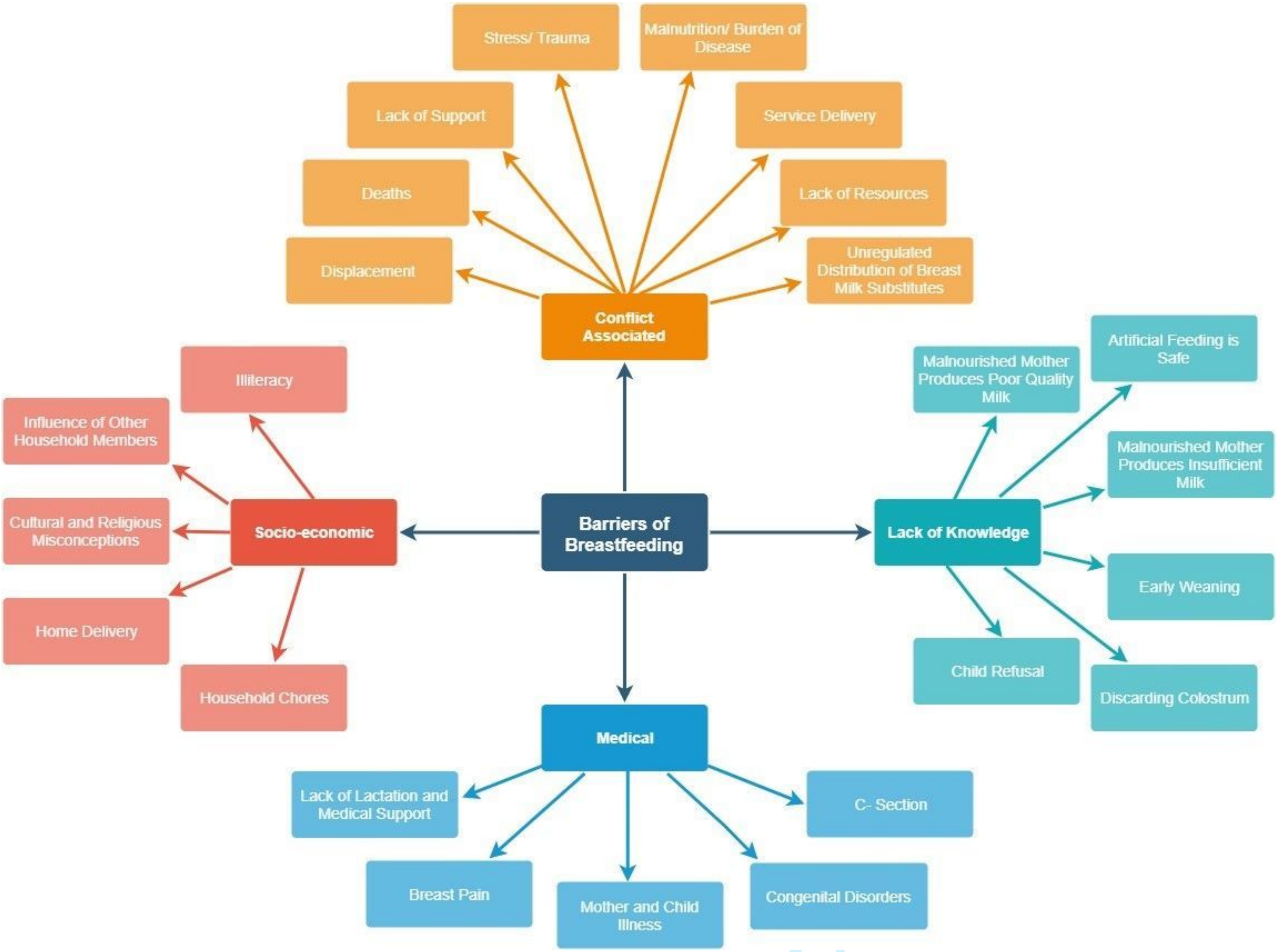

Figure 4: Implementation Guidelines

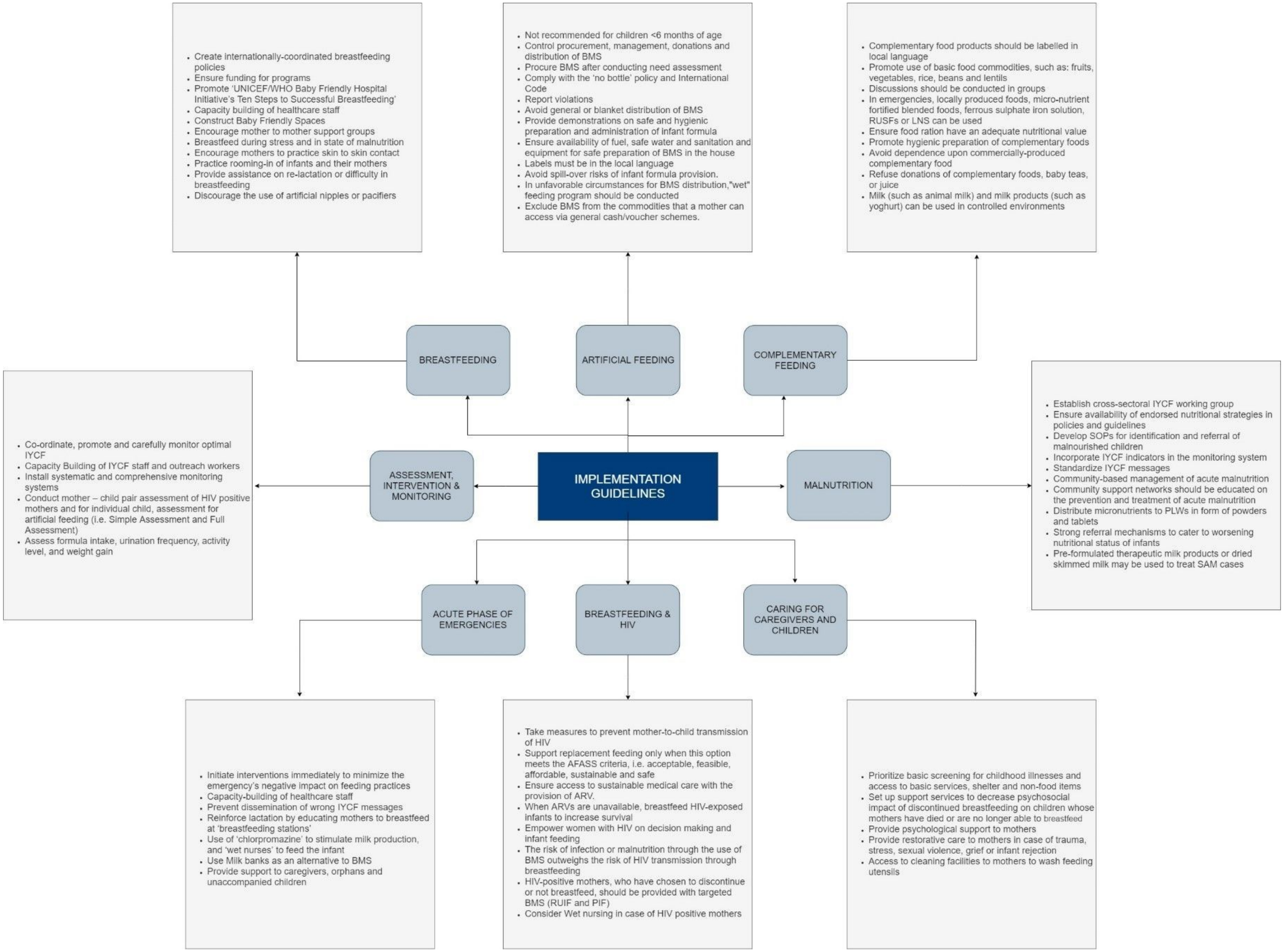

For peer review only

Components of breastfeeding	Operational Guidelines
**I.** Protecting, promoting and supporting breastfeeding	1. Infants should be exclusively breast fed for the first 6 months of life, followed by appropriate complementary feeding along with continued breastfeeding for two[1-14]<br>2. Relief agencies should create internationally-coordinated breastfeeding policies with practical guidelines explicitly stated for field workers and decision[2, 7, 10, 12, 13, 15, 16]<br>3. National policies and technical guidelines on IYCF- E should be explicitly stated and communicated to relevant staff in order to increase preparedness if an emergency strikes. This is particularly important for high-risk countries[2, 12, 13, 17, 18]<br>4. Funding should be provided for programs to support breastfeeding[2, 13, 19]<br>5. It should be ensured that optimal breastfeeding practices and maternal care is promoted according to 'UNICEF/WHO Baby Friendly Hospital Initiative's Ten Steps to Successful Breastfeeding'[2, 10, 20]<br>6. Ensure that relief workers, healthcare staff, technical and non-technical personnel are trained in appropriate infant and young child feeding practices using available training material and key information on IYCF integrated into routine assessment[2, 7, 10, 12-16, 20-23]<br>7. Appropriate and timely support and trainings for breastfeeding and young child feeding should be integrated at all levels of healthcare[2, 16, 21, 23]<br>8. Efforts should be made to raise awareness supporting the superiority of breastfeeding as a life-saving intervention to health personnel, relief staff, NGOs, stakeholders and the general public focusing primarily on pregnant and breastfeeding women[2, 10, 12, 20, 22, 24, 25]<br>9. In conflict and refugee settings, traditional birth attendants (TBAs) are more accessible to mothers than nurses and midwives, therefore TBAs should be trained regarding appropriate breastfeeding practices[17, 20, 24]<br>10. Pregnant women and breastfeeding mothers should be informed using clear language about maternal health, how to properly breastfeed, advantages and maintenance of breastfeeding, negative effects of bottle-feeding and the difficulty to reverse decision not to breastfeed[2, 6, 7, 10, 14, 17, 20, 22, 24, 25]<br>11. Encourage mothers to initiate breastfeeding within the first hour of birth, and to exclusively breastfeed for the first six months of life (do not give them extra water, juices, tea or food) unless medically indicated otherwise. Thereafter, solid foods should be introduced, but breastfeeding should continue for at least a year or two[2, 6, 7, 10, 11, 14, 15, 17, 26]<br>12. Educate mothers to not stop breastfeeding in emergency situations and spread awareness that exclusive breastfeeding provides the best nutrition to babies. Moreover, breastmilk contains ingredients that protects babies from infection, so it's particularly useful in emergency situations[2, 6, 10, 13, 15, 17]<br>13. Encourage mothers to breastfeed on demand[2, 7, 14, 17]<br>14. Practice rooming-in of infants and their mothers (allow mothers and infants to remain together 24 hours a day) to support breastfeeding practice [2, 7]<br>15. Colostrum should be given to the baby to improve its physical growth and feeding baby should not be interrupted in between. The baby lets go off the breast when he/she is done[10, 17, 26]<br>16. Discourage the use of artificial nipples or pacifiers[2, 7, 10, 24]<br>17. Educate mothers on breastfeeding and the procedure to maintain lactation even when the mother-infant pair is temporarily separated [2, 7, 10, 24]<br>18. Ensure that mothers are facilitated by the provision of counseling and other forms of assistance for the purpose of re-lactation or difficulty in breastfeeding[2, 6, 7, 10, 11, 14, 21, 23, 26, 27]<br>19. Mothers should also be made aware that breast milk supply is not reduced by stress, though the release of milk could be affected[6, 8, 10, 14, 18]<br>20. Encourage mothers to practice skin to skin contact as this aids in reducing stress (cortisol) levels and helps the flow of mother's milk. This can be practiced using slings and wraps. Moreover, correct positioning of baby

For peer review only

during breastfeeding is important as effective suckling also triggers breast milk production[6, 10, 11, 14, 15, 17, 24, 26]

21. Malnourished mothers can produce enough milk to breastfeed, however, she should be treated for malnourishment[8, 10, 14, 15, 17, 26]
22. Relief workers should provide nutritional support by giving a general ration to pregnant and lactating women. If full general ration is not possible, then food and micronutrient supplements should be sufficiently provided. This is because an optimally fed mother will be able to optimally feed her infant. Give adequate food to the malnourished mother to prevent depletion of her own nutrients and closely monitor the weight and urine production of the infant[6, 10, 11, 15, 17, 18, 21, 23, 24, 26]
23. In emergency settings, extra breastfeeding support should be provided by encouraging Baby Friendly Spaces, which provide a platform to support mothers/caregivers and promote appropriate infant and young children feeding practices, privacy and safety[2, 11, 12, 15, 18, 21-24, 26]
24. For refugees and displaced populations, establish rest areas for pregnant women/caregivers/mothers and children in transit. These should be secluded, private and culturally appropriate areas that assist women and children to relax and nurse[2, 6, 11, 15, 18, 21, 23, 24]
25. Establish a program to encourage mother to mother/ women to women support[2, 6, 7, 20, 21, 23, 24]
26. Establish registration of newborn infants, i.e. within two weeks of delivery, to ensure timely additional rations and breastfeeding support for lactating and breastfeeding mothers. Nutrition workers should help mothers to establish exclusive breastfeeding[2, 6, 12, 16, 18, 23, 24]
27. Vulnerable groups, especially newly arriving mothers and infants with severe feeding problems should be identified and referred for immediate assistance[2, 6, 12, 15, 16, 18, 21, 23]
28. Create referral and follow-up systems for mothers/caregivers and their infants[2, 3, 6, 7]
29. Avoid giving estrogen containing contraceptive pills to mothers because they decrease breastmilk production[24]
30. Encourage mothers to build their confidence which leads to production of oxytocin to improve blood flow[26]
31. Continue to breastfeed sick children or when they are less hungry. In case they are not hungry then put them to breast more repeatedly to ensure that they take enough breastmilk[6, 10, 11, 14, 17, 26]
32. When natural breastfeeding is not possible, available alternatives should be evaluated before an appropriate choice is made. This may include evaluating between: wet-nursing, the use of milk bank, home-modified milk and the use of locally purchased commercial infant formula or generic unbranded infant formula[10, 12, 15, 23, 24, 28]
33. Access to infant formula should be based on the guidelines set by the WHO International Code of Marketing of Breast Milk Substitutes, 1981[10, 25]
34. Support should be provided for artificial feeding and this should be distinct from the support being provided for breastfeeding[1, 2, 4, 6, 10, 12, 19, 23]
35. Lactating women can take most medicines (including antibiotics) and can be immunized as well, as recommended for adults and adolescents to protect against infectious diseases (measles, mumps, rubella, tetanus, diphtheria, pertussis, influenza, Streptococcus pneumoniae, Neisseria meningitidis, hepatitis A, hepatitis B, varicella, and inactivated polio)[15]
36. In case of radiation exposure
    a. Women who were exposed to radiation should be advised to temporarily stop breastfeeding unless there is no other source of feeding available for the infant. These mothers should pump and discard their milk until the infant can resume breastfeeding
    b. In case of interruption of breastfeeding, preferred source of infant feed is human milk that was pumped and stored prior to the radiation exposure or ready-to-feed infant formula. Mothers should use powdered or concentrated formula only if they are sure that water used to reconstitute is free from radiation

For peer review only

		c. Mothers can resume breastfeeding when advised by local health officials or when they have been evacuated from the radiation affected area<br>d. Mothers do not need to stop breastfeeding if both mother and infant have been given appropriate doses of potassium iodide at the right time, according to the advice of local health officials[15]
**II.**	Protecting non-breastfed infants and minimizing the risks of artificial feeding	1. Breast milk substitute (BMS) is not recommended for children <6 months of age[3, 4, 6, 7, 9, 10, 19, 21, 25, 28]<br>2. Procurement, management and distribution of breastmilk substitutes, bottles and teats, and commercial complementary foods should be controlled during emergencies and should comply with the 'no bottle' policy and International Code and WHA guidelines and violations should be reported[1-3, 10, 13, 16, 19, 21-23]<br>3. Procure BMS after conducting need assessment of artificial feeding at population level assessment. It may also include nutrition survey, household and community survey. This is recommended to be done in close collaboration with government bodies and by developing Program Cooperation Agreements (PCAs) with local bodies for task implementation[28]<br>4. On distribution of BMS, it should be ensured that workforce involved should have enough capacity for assessment, counselling, supply chain management and in providing support to families on WASH and IYCF practices[28]<br>5. Donations of free or subsidized breast-milk substitutes, bottles and teats and commercial baby foods at emergency sites should be refused and advocated against as this could put infants lives at risk and undermine breastfeeding practices[2-4, 6, 8, 11-13, 16, 21-23, 28]<br>6. The distribution, use and quantity of breast milk substitutes should be controlled by collecting unsolicited donations from all ports of entry and the recipient agencies, and should be stored and managed centrally by a single designated agency[1, 2, 4, 6, 10, 12, 13, 16, 19, 21, 23, 28]<br>7. BMS should only be distributed to the infants who really need it and have no viable breastmilk options, based on established criteria (where distribution can be targeted, the supply chain is secure, and the conditions for safe preparation and use can be met). This should be determined by a qualified health or nutrition worker trained in breastfeeding and infant feeding issues[1-4, 6, 7, 9-12, 14-16, 19, 21-23, 27]<br>8. Breast-milk substitutes, milk products, bottles and teats should never be part of a general or blanket distribution and their use should be avoided, especially in case of emergency situations or in situations where hygienic conditions can't be ensured and their use should be discouraged. Bottles should be exchanged for cups instead as it's easier to keep them clean[1-4, 6, 7, 10-12, 16, 18, 19, 21, 23, 24, 26-28]<br>9. Promotion of breast-milk substitutes at the point of distribution should be strictly discouraged. There should be no advertisement or display of products or items with milk company logos and BMS supplies should not be used as a sales inducement and there should be no provision of single tins/samples of BMS or gifts to mothers. Moreover, no incentives should be offered by manufacturers to health workers to promote BMS products[6, 9, 10, 21-23, 25, 28]<br>10. In case of crisis, WHO developmental partners and/or the designated nutrition co-ordinating agency should train as well as support training of staff and mothers on the proper and safe use of infant formula[2, 4, 10, 12, 16, 23]<br>11. When the use of infant formula is indicated, before its distribution, mothers and caregivers responsible for feeding should be educated on the specific care needed by a non-breast fed infant, and given practical training and one-on one demonstrations by a skilled health worker on safe and hygienic preparation and administration of infant formula[1, 2, 4, 6, 7, 10, 12, 19, 21, 23]<br>12. There should be regular follow-up visits, to caregivers of targeted infants receiving BMS comprising of regular infant health and growth monitoring and distribution of BMS should be frequent and regular (at least bimonthly), with minimum delays[1, 3, 7, 10, 12, 19, 21]<br>13. It should also be ensured that there is availability of fuel, safe water and sanitation and equipment for safe preparation of BMS in the house, prior to

For peer review only

	distribution of BMS and implementing a household based program[2-4, 6, 7, 9-11, 14, 15, 21, 23, 25, 26, 28]<br>14. For infants determined to be in need of infant formula, the adequate and continued provision of infant formula must continue for as long as they require it, even if it needs to be purchased. IYCF-E programs that include an infant formula provision component should plan to provide it for at least 6 months, or if there is no availability of adequate complementary food, then 12 months[3, 4, 6, 7, 9-12, 14, 19, 22]<br>15. Non-breastfed infants over 6 months of age, provided with 6 months of BMS, may require 2-4 weeks of buffer supply to offer transition to non BMS feeding[3, 10]<br>16. An improvement should be brought about in health services to manage the adverse effects of artificial feeding, especially the increase in incidence of severe diarrhea and respiratory infections in infants[2, 10]<br>17. Generic, unbranded formula is recommended for infants who require infant formula, followed by locally purchased and relabeled infant formula (to be in compliance with The International Code). Home modified milk should only be used temporarily as the last resort for infants less than 6 months old. The type of infant formula should have a shelf life of at least 6 months and be appropriate for the infant, including their age[4, 6, 7, 9, 10, 15, 19, 21, 23]<br>18. Labels must adhere to the specific labelling requirements of The International Code and should be in the language of the local population. The BMS packaging should have clear instructions, with pictures, on how to use it, along with clearly specifying the superiority of breast milk[4, 6, 7, 9, 19, 21, 25, 28]<br>19. The labels should include a sign of “Important Notice” stating importance of breastfeeding and a statement stating, “The product should be used only on the advice of a health worker as to the need for its use and the proper method of use.” They instructions should also state about its safe preparation and health hazard on inappropriate use, and a warning against the health hazards on inappropriate preparation of infant formula[28]<br>20. Supply department should assure that BMS manufactured should follow Codex Alimentarius standards. UNICEF has not included BMS in it supply catalogue as a non-standard product[28]<br>21. For infants under 6 months of age, the only suitable BMS is infant formula. However, infants over the age of 6 months do not need infant formula but can use other sources of milk (pasteurized full-cream animal milk (cow, goat, sheep), Ultra High Temperature (UHT) milk, fermented milk or yogurt) as these are easier to find and are less dangerous than powdered milk. Condensed milk should not be used for infant feeding[4, 6, 10]<br>22. Liquid milk, if being used, should be consumed within a few hours of opening. Baby juices and teas should be avoided as they are low in nutrition and high in sugar. For infants over 6 months of age, infant formula can be mixed into the child’s food instead of giving it to drink[6]<br>23. Milk products should only be received and distributed in a dry form and even dried milk products should be distributed only when pre-mixed with a milled staple food and should not be distributed as a single commodity. However, dried skim milk is not an appropriate BMS for infant, and even for older children it must be fortified with vitamin A and not given on its own[4, 7, 9, 19, 23]<br>24. It should be ensured that there are no spill-over risks of infant formula provision. Measures should be taken to reduce spill-over by ensuring that feeding BMS to a minority of children doesn’t undermine breastfeeding practices of the majority. Advertising of infant formula should be stopped, and the provision of infant formula should be discrete, monetary support should be provided to breastfeeding mothers, and a separate space dedicated for breastfeeding support and counseling[1, 2, 4, 6, 10, 12, 19, 23]<br>25. When circumstances are not favorable for BMS distribution (safe preparation and use of infant formula can’t be ensured), an on-site supplementary "wet" feeding program should be conducted in closed spaces under supervision[4, 7, 21, 23]<br>26. Infant formula should not be excluded from the commodities that a mother can access via general cash/voucher schemes. However, it should be

For peer review only

	accompanied by interventions such as providing essential information on breastfeeding and on how to reduce the risks of formula feeding[6]<br>27. Breast milk donations should be authorized and be regulated by the Ministry of Health. Until the arrival of the beneficiary, cold chain should be maintained for donated frozen breast milk[2, 26, 28]<br>28. The psychosocial impact of discontinued breastfeeding on children whose mothers have died or are no longer able to breastfeed should also be considered and support services should be set up to address this[12, 16]<br>29. One guideline mentioned that it should be known and taken care of that transitioning out from an emergency IYCF-E program with an infant formula provision component requires a solid, long-term exit strategy with ties to both facility and community-based structures[12]
**III.** Protecting, promoting and supporting appropriate, safe and timely complementary feeding	1. To promote growth and development of infants, encourage appropriate, timely and safe complementary feeding for infants (aged > 6 months) and young children (aged between 12 to < 24 months), along with continued breastfeeding[1-7, 9, 11, 12, 14, 18, 23]<br>2. Children over the age of 6 months of age should be given complementary foods (solid, semi-solid and soft foods) that are easy to eat and digest and nutritionally complement breast milk[1, 2, 6, 18]<br>3. Conduct discussion in groups on complementary feeding and weaning[26]<br>4. Promote the growth of locally produced food and encourage mothers/caregivers to use these locally-produced, inexpensive foods for complementary feeding. These include basic food commodities, such as: fruits, vegetables, rice, beans and lentils[9, 12, 18, 23]<br>5. Increase frequency and variety of complementary food with the growing age of child to meet his/her nutritional needs[26]<br>6. In emergencies, locally produced foods, micro-nutrient fortified blended foods, ferrous sulphate iron solution (iron drops), Ready-to-Use Supplementary Foods (RUSFs) or Lipid based Nutrient Supplements (LNS) can be used for complementary feeding depending on the nutritional situation[12, 18, 23, 28]<br>7. Special attention must be given to ensure that the food ration distributed for old infants and young children have an adequate nutritional value (food should be fortified with vitamins and minerals[4, 6, 16, 20, 23]<br>8. Establish services to extend nutritional support to vulnerable groups, such as: orphans and unaccompanied infants and young children[2, 6, 12, 15, 16, 21, 23]<br>9. Promote hygienic preparation of complementary foods by providing mothers/caregivers necessary information and support[6, 23]<br>10. In emergencies, special consideration should be made to mitigate the obstacles faced by mothers/caregivers in the preparation (cook, mash, etc.) of age-appropriate complementary foods. Measures needed to counter these obstacles should be incorporated in the program design[6, 12, 18]<br>11. IYCF-E programs relating to complementary feeding should include:<br>a. Group sensitization and education<br>b. Develop local food recipes focusing on nutritional value, affordability and food dietary diversity<br>c. Provide cooking demonstrations, and<br>d. Support mothers/caregivers to prepare complementary foods via initiatives such as fresh food vouchers[12]<br>12. Dependence upon commercially-produced complementary foods should be avoided[9, 23]<br>13. Mothers should be assured that in the absence of safe complementary foods, breastmilk is a significant source of nutrition for infants the first year of life and beyond[15]<br>14. Donations of complementary foods, baby teas, or juices should be refused. If there are donated supplies, they should be directed to the designated coordinating agency on nutrition/health to be managed appropriately[6]<br>15. Promotion of a varied diet, dairy products in particular, to ensure that energy, protein, mineral and vitamin requirements are met. Moreover, when preparing food for infants such as porridges, mashed potatoes, etc., milk and infant formula can be added to it to increase the nutrient content[4, 6, 20, 26]

For peer review only

		16. Pastoral communities mainly use milk and milk products, which contains a significant amount of nutrition for children over six months. As a part of complementary feeding, milk (such as animal milk) and milk products (such as yoghurt) can be provided to infants over 6 months of age. In such cases, distribution of milk products can only be conducted in controlled environments under strict supervision, such as on-the-spot feeding[3]<br>17. Good nutrient sources for infants are animal source foods, such as yogurt and cheese whereas products containing only fruits/vegetables are less energy and nutrient dense. Baby teas and juices should not be given to infants as they do not have much nutrient value[6]<br>18. Complementary food products should be labelled in the language of the targeted population and be acceptable to them, according to their culture. The products should also have information on how to prepare the food[6]<br>19. Moreover, these products should not have images of bottle feeding on them or recommend it in any way[6]<br>20. If needed, commercial 'baby' foods should be purchased and distributed to mothers or products recommended to mothers, for infants over 6 months of age, that have the most nutritional value[6]<br>21. It is recommended that children over the age of 12 months eat the same foods as older children[6]
**IV.**	Caring for care-givers	1. Efforts should be made to increase caregivers' coping capacity because the number of caregivers is often reduced during emergencies and stress levels increase, so psychological support and encouragement plays an important role in enhancing optimal IYCF-E practices[1, 2, 15, 17, 18]<br>2. Treat the mother during illness, keep the infant close to the mother and do not stop breastfeeding. The mother should be educated on the appropriate method of milk expression as she can maintain breastmilk flow through milk expression[17, 24]<br>3. Breastfeeding mothers require identification, protection and active support. Conflict situations and displacement can negatively affect maternal confidence and breastfeeding practices. Breastfeeding mothers tend to stop or reduce breastfeeding in such situations and thus a lot of support and counselling should be provided to newly arriving refugee mothers, caregivers and infants with special needs (orphans and unaccompanied children)[2, 5, 6, 12, 17, 18, 21, 23]<br>4. Provide restorative care to mothers in case of trauma, stress, sexual violence, grief or infant rejection[24]<br>5. Adequately trained and skilled staff should support mothers with difficulties in breastfeeding their infants and provide private safe spaces for mothers to breastfeed and to connect with other mothers. Access to cleaning facilities should also be provided to mothers to wash feeding utensils, especially to mothers who are formula feeding. Prioritize mothers of infants and young children for basic screening of childhood illnesses, access to registration and basic services, shelter and non-food items[2, 6, 10, 15, 18]<br>6. It should be ensured that mothers/caregivers of artificially fed infants are given targeted support and receive counselling as well as kits for preparing BMS safely, including soap, fuel, water purification tablets[2, 18]
**V.**	Protecting Children	1. Artificially-fed infants need more protection and support than breast-fed children[6, 10]<br>2. Infants are at higher risk of malnutrition and illness like diarrhea and chest infections if they are being fed infant formula. In emergency conditions, this could be fatal[2, 6, 10, 11]<br>3. In case the infant is ill (e.g. if the infant has diarrhea), continue feeding the infant since breastmilk contains water to replace losses through diarrhea and important minerals and vitamins to help prevent dehydration. It also contains proteins to help strengthen the immune system of the baby. Give smaller amounts of breastfeed and more frequently if the infant's appetite is reduced. However, in serious or prolonged cases of diarrhea, rehydration therapy may be required[6, 10, 11, 15, 26]<br>4. Newborn infants are the most vulnerable group which should be targeted and ensured that they are exclusively breast fed and artificial feeding is prevented[2, 6, 12, 16, 21, 23]

For peer review only

	5. Support breastfeeding as the best way to safeguard infants against cholera. Infants with cholera should continue breastfeeding as soon as they are able to suckle, and mothers with cholera should re-initiate breastfeeding as soon as they are stable[12]<br>6. Efforts should be made to protect cholera-free breast-fed babies from cross-contamination[12]<br>7. IYCF managers, field staff and registration staff should collaborate and ensure screening of infants at registration to identify and refer infants at risk or those who are highly vulnerable (i.e. orphans and unaccompanied infants and young children, non-breastfed infants <6 months, ill infants or malnourished infants) and establish services to give these vulnerable infants nutritional support[2, 6, 12, 16, 18, 21, 23]<br>8. Prioritize support service:<br>a. For re lactation: Prioritize non-breastfed infants 0 - <6 months or of 0 - <2 months whose mother/wet nurse is willing to re lactate<br>b. Re-establish exclusive breastfeeding: Always prioritize infants aged 0 - <2 months (including newborns). Infants 0 - <4 months are a priority, but it's an ideal situation for infants 0 - <6 months<br>c. BMS provision and associated support services: prioritize not breastfed infants who are <6 months[3]<br>9. The psychosocial impact of discontinued breastfeeding on children whose mothers have died or are no longer able to breastfeed should also be considered and support services should be set up to address this[12, 16]<br>10. Consideration should also be given to special needs of artificially fed children (0-23 months) and PLW (i.e. insecticide treated mosquito nets; hygiene items including soap and washing containers; cooking and eating items; breastfeeding shawls; blankets and infant/young child clothing and shoes with thermal needs considered; potties, diapers), with a particular preference for items that can be locally sourced[2]
**VI.** Malnutrition	1. Ensure availability of endorsed nutritional strategies in policies and guidelines and establish cross-sectoral IYCG working group to discuss challenges, needs and success[2]<br>2. IYCF teams should coordinate and generate a treatment plan where mothers should be admitted at stabilization centers along with her children where they would be provided with nutritional support and counselling to improve lactation[2]<br>3. Select an IYCF specialist from each nutrition post or community group[2]<br>4. Standardize IYCF messages for caregivers and PLWs with children of 0-23 months and incorporate IYCF indicators in the monitoring system[2]<br>5. Develop standard procedure for health workers for identification and referral of malnourished children[2]<br>6. Promote IYCF practices (i.e. early initiation, exclusive breastfeeding and complementary feeding)[2]<br>7. Timely introduction of complementary food in infants of 6-9 months[2]<br>8. Micronutrients should be distributed to all PLWs in form of powders and tablets[2]<br>9. Malnutrition treatment and prevention programs should incorporate and prioritize infant and young child feeding in their agenda[2, 12]<br>10. Efforts should be made to investigate the underlying cause of malnutrition and measures should be taken to correct it[1, 2, 10]<br>11. Monitor nutritional status of mothers, infants and young children with the purpose of identifying, assessing, preventing and treating malnourished children[1, 2, 10]<br>12. Strong referral mechanisms should be put in place for acute malnutrition to cater to worsening nutritional status of infants[2, 12]<br>13. Admit malnourished children along with their mothers to a nutritional rehabilitation program[12, 21, 24]<br>14. There should be community-based management of acute malnutrition, if conditions are favorable. In disasters, supplementary feeding should be the primary strategy for prevention and treatment of moderate acute malnutrition (MAM). Depending on vulnerable population groups and malnutrition level/risk

For peer review only

	of an increase in acute malnutrition, supplementary feeding can be blanket or targeted[2]<br>15. Pre-formulated therapeutic milk products or dried skimmed milk (DSM) may be used to treat cases of severe acute malnutrition (SAM). However, attention must be given to ensure that supplementary food aid commodities are carefully regulated and distributed to only target vulnerable groups[4, 9]<br>16. Therapeutic milks are not appropriate for BMS use. Therapeutic milk like F75 and F100 should be used for the treatment of children with severe acute malnutrition (SAM). It can be given to infants less than 6 months. SAM children require appropriate treatment at right time with immediate referral[28]<br>17. Cash/voucher programmes (conditional) could be started that promote good nutrition outcomes i.e. preventing malnutrition. Unconditional cash programs should be promoted in case of food security and livelihood. But in this case optimal IYCF practices should be considered through an expert. Women should be educated and sensitized for appropriate use on food and nonfood items for children of 0-23 months[2]<br>18. Community support networks should be educated on the prevention and treatment of acute malnutrition[2]
**VII.** The acute phase of emergencies (prevention through interventions)	1. In case of an emergency, interventions should start immediately to minimize the emergency's negative impact on feeding practices and every agency should develop a policy on infant feeding in emergencies, focusing on supporting caregivers and nutritional needs children[1, 2, 4, 23]<br>2. An appropriate agency should be appointed and resourced at the start of an emergency to co-ordinate IYCF-E practices and ensure the implementation of policies and it should be conveyed to all agencies working in the area[13]<br>3. In emergencies, donations of BMS are not needed and may put endanger infant lives due to poor hygienic conditions[2, 4, 10, 12, 13, 23]<br>4. If emergencies occur in places where there was already high infant formula use, promotion of IYCF-E can be even more difficult and WHO, and its developmental partners, along with local authorities and/or the national Nutrition Cluster (if activated) should ensure that appropriate IYCF-E is adequately promoted, protected and supported[2, 10, 12, 23]<br>5. Interventions should be undertaken to increase the prevalence of appropriate IYCF-E practices such as culturally-appropriate behavior -change approaches, along with capacity-building, to increase the rate of exclusive breastfeeding[12, 13, 23]<br>6. A joint statement for protection and support of appropriate IYCF-E should be released and ensured that BMS donations and distributions are carefully monitored[2, 4, 7, 12, 13, 23]<br>7. WHO, its developmental partners, local governments and national Nutrition Cluster (if activated) should work on this and provide this information to all staff, potential donors (including governments and the military) and the media, and ensure that no wrong messages are being disseminated both in emergency preparedness and particularly during the early phase of an emergency response[2, 4, 12, 13, 23]<br>8. Breastfeeding and IYCF support should be a major component of all services for mothers, infants and children and measures should be put in place to ensure that their needs are met in the early stages of an emergency[2, 4, 7, 16]<br>9. Support should also be provided to caregivers and infants with special needs (orphans and unaccompanied children)[2, 6, 12, 14-16, 21, 23]<br>10. It should be ensured that artificial feeding is strictly restricted to the targeted group of infants that require it and mothers who need help with breastfeeding are provided lactation support by mobilizing 'local breastfeeding facilitators'[7, 10]<br>11. Lactation should be reinforced by educating mothers to breastfeed every 2-3 hours at 'breastfeeding stations' scattered across refugee sites[7]<br>12. One guideline also suggested the use of 'chlorpromazine' to stimulate milk production, according to the protocol and also 'wet nurses' to feed the infant[7]<br>13. Milk banks can also be used as an alternative to BMS and also as a source of employment in emergency setting[7, 10]

For peer review only

**VIII.**	Assessment, intervention and monitoring	1. In emergencies, co-ordinate, promote and carefully monitor optimal feeding in infants and young children[1, 2, 15, 23]<br>2. There should be systematic and comprehensive monitoring systems to track all infant feeding products being distributed[13]<br>3. Conduct mother – child pair assessment of HIV positive mothers and for individual child, assessment for artificial feeding (i.e. Simple Assessment and Full Assessment)[3, 24]<br>4. In areas with high prevalence of HIV, the risk of infant getting HIV via breastfeeding should be weighed against the risk of facing infection and malnutrition as a consequence of not being breastfed[1]<br>5. The prevalence of HIV in the affected population, knowledge of HIV status, and availability of counselling and testing facilities should be assessed (including pre-emergency estimates) using secondary sources and relevant information from health information systems[10]<br>6. In emergencies, implement an IYCF-E program focusing on infant feeding provision, with robust mechanisms that estimate the number of children that don't have access to breast milk and then provide them with targeted supply of infant formula[12]<br>7. Monitor the nutritional status of infants and young children, particularly weight monitoring for those receiving formula feed and assess intake, urination frequency, activity level, whether infant is feeding vigorously and weight gain[6, 10, 12, 15, 24, 26]<br>8. Establish a strong referral system to treat acute malnutrition should the infant's nutritional status deteriorate[2, 12]<br>9. To monitor and to conduct rapid assessments, gather information and statistics regarding: demographic profile, morbidity, mortality, predominant feeding practice, reported feeding problems for infants and young children including problems related to breastfeeding and complementary feeding, pre-crises approach to orphaned children, security risks and availability of conspicuous BMS products and bottles/ teats/ breast pumps[7, 10, 21, 23]<br>10. To monitor and assess, use qualitative methods to gather data regarding:<br>a. Appropriate complementary foods in the general ration or targeted feeding programs<br>b. Maternal and child health facilities including antenatal, delivery, postnatal and child care<br>c. Capacity of potential support-givers including breastfeeding mothers, trained health workers, trained counselors and experienced women from the community<br>d. Factors that may disturb breastfeeding practices<br>e. Key decision-makers at household, community and local health facility level that may influence infant and young child feeding practices<br>f. Cultural barriers affected practices of re-lactation, wet-nursing, etc.<br>g. General health environment including: water and sanitation, housing, facilities of food preparation and cooking[10, 21, 23, 28]<br>11. To monitor and assess, use quantitative methods to gather data regarding:<br>a. Estimated number of unaccompanied and accompanied children under two years of age, pregnant and lactating women<br>b. Statistics regarding morbidity, mortality, and levels of malnutrition<br>c. Information concerning nutritional adequacy of food rations<br>d. Pre-crisis and recent patterns in infant and young child feeding practices<br>e. Availability and management of BMS in accordance to The International Code[2, 10, 21, 23]<br>12. Governments should monitor and apply The International Code collaboratively with the assistance of International agencies such as WHO and UNICEF, NGO's, refugee camp staff, professional groups and customer organizations to ensure that manufacturers and distributors of BMS remain within the scope of the established Code[2, 10, 25]<br>13. Manufacturers and distributors should monitor their market prices and the practices of their marketing personnel in accordance to The Code. Non-

For peer review only

	governmental organizations (NGO's) along with professional groups, institutions and concerned individuals should monitor and criticize manufacturers and distributors that don't follow the principles of The Code[10, 25]<br>14. Review and monitor the following:<br>a. Advice and knowledge regarding breastfeeding and BMS usage<br>b. Estimate the number of women breastfeeding, weaning and incorporating the use of BMS and bottle in the feed of their infant and young children<br>c. Constraints associated with hygienic BMS preparation<br>d. Availability and management of BMS[4, 7, 10, 20, 21, 23, 25]<br>15. Promote importance of breastfeeding and optimal hygiene practices especially handwashing before preparation of BMS. We should also dispel myths among mothers regarding breastfeeding[28]<br>16. Capacity Building of IYCF staff and outreach workers on nutrition (optimal IYCF practices, lifesaving IYCF practices, rapid IYCF assessment and detection of poor IYCF practices)[2]
**IX.** Breastfeeding, HIV, and other considerations	1. Generate policies on empowering women with HIV on decision making and infant feeding[8]<br>2. IYCF staff should take appropriate measures to prevent mother-to-child transmission of HIV, as well as focus on improving child survival from HIV[2]<br>3. Promote the use of optimal infant and young child feeding guidelines when the HIV status of the mother is unknown or she is HIV negative[2-4, 10, 23, 24, 29]<br>4. In case of unavailability of HIV-testing, it is recommended to breastfeed the infant for six months, followed by adequate complementary feeding and continued breast feeding for two years[24, 29]<br>5. Mothers should know their HIV status and receive appropriate counselling. Those diagnosed as HIV positive should make an informed decision about feeding options by balancing the prevention of HIV transmission with the nutritional requirements of infants[1, 2, 4, 8, 10, 12, 23, 24, 29]<br>6. HIV positive mother should exclusively breastfeed her child for first 6 months of life unless replacement feeding is affordable, sustainable and safe for their infants. In case, replacement feeding is not acceptable, then complementary feeding with continued breastfeeding at 6 months is recommended, while mother and baby will be assessed regularly[2, 10, 24]<br>7. Supportive arrangements and personal attachment for HIV positive mothers helps to reduce isolation, build confidence, reduce conflicting messages, encourage age appropriate feeding, provide privacy and educate family members[24]<br>8. Ensure access to sustainable medical care for mothers with an HIV positive status by supporting the provision of ART and ARV. If due to emergencies, the supply of these medications is hindered then immediate action should be taken for its re-establishment[12, 23, 24, 29]<br>9. In circumstances during acute emergencies, when Antiretroviral Drug (ARVs) are unavailable, it is recommended to breastfeed HIV-exposed infants to increase his/her survival[2, 3]<br>10. In emergency contexts, HIV-positive mothers should be supported to initiate or continue exclusive breastfeeding/ continued breastfeeding with adequate complementary feeding depending upon the age of the infant. The risk of infection or malnutrition through the use of Breast Milk Substitutes (BMS) outweighs the risk of HIV transmission through breastfeeding[2, 8, 12, 21, 23, 24]<br>11. Support replacement feeding *only* when this option meets the AFASS criteria, i.e. acceptable, feasible, affordable, sustainable and safe[3-5, 23, 24, 29]<br>12. All HIV- positive mothers should receive full support and get regular follow ups[2, 29]<br>13. HIV-positive and caregivers of children born to HIV-positive mothers, who have chosen to discontinue or not breastfeed, should be provided with targeted, appropriate, breast milk substitutes (Ready to Use Formula (RUIF) and Powdered Infant Formula (PIF)[2, 12]<br>14. Promote and support specific counseling concerned with risks of mixed feeding and HIV transmission. Additionally, ensure the provision of Safe BMS kits, i.e. adapted to the type of BMS administered[2, 12]

For peer review only

	15. Services and activities linked to the Prevention/Elimination of Mother-to-Child Transmission (E/PMTCT) should be provided routinely as a part of nutritional interventions[2, 12]<br>16. Measures taken by ICYF-E staff should be sensitive and should avoid actions that may exacerbate any HIV-related stigma[2, 12]<br>17. Wet nursing should also be considered in case of HIV positive mothers and for infants who have lost their mothers. It should be administered by any person (other than mother). The wet nurse should be counselled before and after wet nursing to prevent her from catching infection[10, 12, 24, 29]<br>18. WHO recommends flash heated breastmilk rather than boiling breast milk to prevent significant nutritional damage of breastmilk[29]

References:

1. WHO. Guiding principles for feeding infants and young children during emergencies. 2004.
2. UNHCR, children St. Infant and Young Child Feeding in Refugee Situations:

A Multi-Sectoral Framework for Action. 2018 May, 2018.
3. UNHCR. Infant and young child feeding practices

Standard Operating Procedures for the Handling of Breastmilk Substitutes (BMS) in Refugee Situations for children 0-23 months. 2015 August 2015.
4. UNHCR. UNHCR Policy Related To The Acceptance, Distribution And Use Of Milk Products In Refugee Settings 2006.
5. Save the Children U, Food Security and Nutrition Network. ICFY-E Toolkit: Summary of Feeding the Non-breastfed child 6- 24 months of Age 2007.
6. Refugees UHCf. Interim Operational Considerations for the feeding support of Infants and Young Children under 2 years of age in refugee and migrant transit settings in Europe. 2015.
7. OXFAM. Feeding in Emergencies For Infants Under Six Months: Practical Guidelines 1996.
8. Machel G. Impact of Armed Conflict on Children. United Nations; 1996 26 August 1996.
9. Joint UNICEF U, WFP, WHO Statement Policy on infant feeding in the Balkan Region.
10. ENN. Infant Feeding in Emergencies: Policy, Strategy and Practice. 1999 May 1999.
11. Association ILC. Breastfeeding: A Vital Emergency Response. Are You Ready? . 2009. p. 2.
12. ACF-International. Infant and Young Child Feeding in Emergencies. 2015 December 2015.
13. Borrel A, Taylor A, McGrath M, Seal A, Hormann E, Phelps L, et al. From policy to practice: challenges in infant feeding in emergencies during the Balkan crisis. Disasters. 2001;25(2):149-63.
14. Program WIEPoB. Infant and Young Child Feeding in Emergency Situations. 2005.
15. Pediatrics AAo. Infant feeding in disasters and emergencies. 2015.
16. Save the Children U, Food Security and Nutrition Network. IYCF-E Toolkit: Summary of Operational Guidance on ICF-E Key Points and WHA Resolution. 2007.
17. UNHCR. HOW TO GUIDE reproductive health in refugee setting (Strengthening Safe Motherhood Services). 1999 November 1998.
18. Children St. Assessment of infant and young child feeding practices among refugees on Lesvos Island, Greece. 2016.
19. Focus I. The Code and infant feeding in emergencies. 2009 May 2009.
20. USAID TSG, International Medical Corps Camp-based Barrier Analysis of Early Initiation of Breastfeeding, Iron-rich Food Consumption, and Early Antenatal Care Seeking Behaviors of Syrian Refugees in Azraq Camp, Jordan. 2016.

For peer review only

21. WHO, UNICEF, LINKAGES, IBFAN, ENN. Infant feeding in emergencies Module 1 (for emergency relief staff). 2001 November 2001.

22. WHO. WHA RESOLUTION 47.5

Infant and young child nutrition. 1994.

23. Group IC. Infant and Young Child Feeding in Emergencies Operational Guidance for Emergency Relief Staff and Programme Managers

2017 October 2017.

24. ENN, IBFAN-GIFA, Fondation Terre des hommes, Action Contre la Faim, CARE USA, LINKAGES, et al. Infant feeding in emergencies Module 2 Version 1.1 (for health and nutrition workers in emergency situations). 2007.

25. WHO. International Code of Marketing of Breast-milk Substitutes. Geneva; 1981.

26. ACF-International. Baby friendly spaces

Holistic approach for Pregnant, Lactating Women anf their very young children in Emergency. 2014 December 2014.

27. Action Against Hunger U. Infant Feeding and Weaning Report 2000.

28. UNICEF. UNICEF Guidance on the provision and use of breastmilk substitutes in humanitarian settings. 2018 June 2018.

29. UNHCR. Guidance on Infant feeding and HIV in the context of refugees and displaced populations. 2008 April 2008.

For peer review only

PRISMA 2009 Checklist

Section/topic	#	Checklist item	Reported on page #
**TITLE**			
Title	1	Identify the report as a systematic review, meta-analysis, or both.	1
**ABSTRACT**			
Structured summary	2	Provide a structured summary including, as applicable: background; objectives; data sources; study eligibility criteria, participants, and interventions; study appraisal and synthesis methods; results; limitations; conclusions and implications of key findings; systematic review registration number.	2
**INTRODUCTION**			
Rationale	3	Describe the rationale for the review in the context of what is already known.	4,5
Objectives	4	Provide an explicit statement of questions being addressed with reference to participants, interventions, comparisons, outcomes, and study design (PICOS).	4,5
**METHODS**			
Protocol and registration	5	Indicate if a review protocol exists, if and where it can be accessed (e.g., Web address), and, if available, provide registration information including registration number.	
Eligibility criteria	6	Specify study characteristics (e.g., PICOS, length of follow-up) and report characteristics (e.g., years considered, language, publication status) used as criteria for eligibility, giving rationale.	5
Information sources	7	Describe all information sources (e.g., databases with dates of coverage, contact with study authors to identify additional studies) in the search and date last searched.	6
Search	8	Present full electronic search strategy for at least one database, including any limits used, such that it could be repeated.	6
Study selection	9	State the process for selecting studies (i.e., screening, eligibility, included in systematic review, and, if applicable, included in the meta-analysis).	6
Data collection process	10	Describe method of data extraction from reports (e.g., piloted forms, independently, in duplicate) and any processes for obtaining and confirming data from investigators.	6
Data items	11	List and define all variables for which data were sought (e.g., PICOS, funding sources) and any assumptions and simplifications made.	6
Risk of bias in individual studies	12	Describe methods used for assessing risk of bias of individual studies (including specification of whether this was done at the study or outcome level), and how this information is to be used in any data synthesis.	NA
Summary measures	13	State the principal summary measures (e.g., risk ratio, difference in means).	NA
Synthesis of results	14	Describe the methods of handling data and combining results of studies, if done, including measures of consistency (e.g., I^2) for each meta-analysis.	NA

For peer review only - http://bmjopen.bmj.com/site/about/guidelines.xhtml

PRISMA 2009 Checklist

Section/topic	#	Checklist item	Reported on page #
Risk of bias across studies	15	Specify any assessment of risk of bias that may affect the cumulative evidence (e.g., publication bias, selective reporting within studies).	NA
Additional analyses	16	Describe methods of additional analyses (e.g., sensitivity or subgroup analyses, meta-regression), if done, indicating which were pre-specified.	NA
**RESULTS**			
Study selection	17	Give numbers of studies screened, assessed for eligibility, and included in the review, with reasons for exclusions at each stage, ideally with a flow diagram.	7
Study characteristics	18	For each study, present characteristics for which data were extracted (e.g., study size, PICOS, follow-up period) and provide the citations.	7
Risk of bias within studies	19	Present data on risk of bias of each study and, if available, any outcome level assessment (see item 12).	NA
Results of individual studies	20	For all outcomes considered (benefits or harms), present, for each study: (a) simple summary data for each intervention group (b) effect estimates and confidence intervals, ideally with a forest plot.	NA
Synthesis of results	21	Present results of each meta-analysis done, including confidence intervals and measures of consistency.	NA
Risk of bias across studies	22	Present results of any assessment of risk of bias across studies (see Item 15).	N/A
Additional analysis	23	Give results of additional analyses, if done (e.g., sensitivity or subgroup analyses, meta-regression [see Item 16]).	NA
**DISCUSSION**			
Summary of evidence	24	Summarize the main findings including the strength of evidence for each main outcome; consider their relevance to key groups (e.g., healthcare providers, users, and policy makers).	16
Limitations	25	Discuss limitations at study and outcome level (e.g., risk of bias), and at review-level (e.g., incomplete retrieval of identified research, reporting bias).	18
Conclusions	26	Provide a general interpretation of the results in the context of other evidence, and implications for future research.	18
**FUNDING**			
Funding	27	Describe sources of funding for the systematic review and other support (e.g., supply of data); role of funders for the systematic review.	21

From: Moher D, Liberati A, Tetzlaff J, Altman DG, The PRISMA Group (2009). Preferred Reporting Items for Systematic Reviews and Meta-Analyses: The PRISMA Statement. PLoS Med 6(7): e1000097. doi:10.1371/journal.pmed1000097

For more information, visit: **www.prisma-statement.org**.

For peer review only - http://bmjopen.bmj.com/site/about/guidelines.xhtml

BMJ Open

A systematic review of Infant and Young Child Feeding Practices in Conflict Areas: What the evidence advocates

Journal:	*BMJ Open*
Manuscript ID	bmjopen-2020-036757.R1
Article Type:	Original research
Date Submitted by the Author:	16-Jun-2020

Complete List of Authors:	Rabbani, Amna; Aga Khan University<br>Padhani, Zahra Ali; Aga Khan University, Paediatrics<br>A. Siddiqui, Faareha; Institute of Business Administration Karachi,<br>Das, Jai K.; Aga Khan University<br>Bhutta, Zulfiqar; Hospital for Sick Children

**Primary Subject Heading**:	Public health
Secondary Subject Heading:	Public health, Nutrition and metabolism
Keywords:	PUBLIC HEALTH, Health policy < HEALTH SERVICES ADMINISTRATION & MANAGEMENT, Community child health < PAEDIATRICS

SCHOLARONE™ Manuscripts

I, the Submitting Author has the right to grant and does grant on behalf of all authors of the Work (as defined in the below author licence), an exclusive licence and/or a non-exclusive licence for contributions from authors who are: i) UK Crown employees; ii) where BMJ has agreed a CC-BY licence shall apply, and/or iii) in accordance with the terms applicable for US Federal Government officers or employees acting as part of their official duties; on a worldwide, perpetual, irrevocable, royalty-free basis to BMJ Publishing Group Ltd ("BMJ") its licensees and where the relevant Journal is co-owned by BMJ to the co-owners of the Journal, to publish the Work in this journal and any other BMJ products and to exploit all rights, as set out in our licence.

The Submitting Author accepts and understands that any supply made under these terms is made by BMJ to the Submitting Author unless you are acting as an employee on behalf of your employer or a postgraduate student of an affiliated institution which is paying any applicable article publishing charge ("APC") for Open Access articles. Where the Submitting Author wishes to make the Work available on an Open Access basis (and intends to pay the relevant APC), the terms of reuse of such Open Access shall be governed by a Creative Commons licence – details of these licences and which Creative Commons licence will apply to this Work are set out in our licence referred to above.

Other than as permitted in any relevant BMJ Author's Self Archiving Policies, I confirm this Work has not been accepted for publication elsewhere, is not being considered for publication elsewhere and does not duplicate material already published. I confirm all authors consent to publication of this Work and authorise the granting of this licence.

For peer review only

A systematic review of Infant and Young Child Feeding Practices in Conflict Areas: What the evidence advocates

Amna Rabbani[1], Zahra A. Padhani[1], Faareha A Siddiqui[2], Jai K Das[1], Zulfiqar A Bhutta[3,4]

1. Division of Women and Child Health, Aga Khan University, Karachi, Pakistan
2. Institute of Business Administration, Karachi, Pakistan
3. Center of Excellence in Women and Child Health, Aga Khan University, Karachi, Pakistan
4. Centre for Global Child Health, The Hospital for Sick Children, Toronto, Canada

Amna Rabbani - amnarabbani@gmail.com
Zahra A. Padhani - zahra.feroz@aku.edu
Faareha A Siddiqui – fsiddiqui@khi.iba.edu.pk
Jai K Das - jai.das@aku.edu
Zulfiqar A Bhutta - zulfiqar.bhutta@sickkids.ca

Corresponding Author

Zulfiqar A Bhutta
Centre for Global Child Health, The Hospital for Sick Children
686 Bay Street, Toronto, ON M5G 0A4, Canada
Tel: +1 416 813 7654x328532
Email: zulfiqar.bhutta@sickkids.ca

For peer review only

ABSTRACT

Background

Breastfeeding in conflict settings is known to be the safest way to protect infant and young children from malnourishment and increased risk of infections. This systematic review assesses the evidence on infant and young child feeding (IYCF) practices in conflict settings.

Methodology

We conducted a search in PubMed and CENTRAL and also searched for grey literature from the year 1980 to August, 2019. We included studies conducted in settings inflicted with armed conflict; which comprised of settings undergoing conflict, as well as, those within five years of its cessation. Studies were included if they discussed IYCF practices, barriers, programs and guidelines to promote and improve IYCF practices. Two review authors independently evaluated and screened studies for eligibility and extracted data; followed by a descriptive and thematic analysis.

Results

We included 56 studies in our review including 11 published articles and 45 reports from grey literature and broadly classified into four pre-determined sections: Epidemiology (n=24), barriers/enablers (n=18), programs/interventions (n=15) and implementation guidelines (n=30). Epidemiological evidence shows that IYCF practices were generally poor in conflict settings with median prevalence of exclusive breastfeeding at 25%, continued breastfeeding at 29%, bottle-feeding at 58.3%, introduction to solid, semi-solid or soft foods at 71.1% and minimum dietary diversity at 60.3%.

IYCF practices were affected by displacement, stress, maternal malnutrition and mental health, family casualties and free distribution of BMS. To improve IYCF, several interventions were implemented; including, training of health workers, educating mothers, community networking and mobilization, lactation-support service, baby friendly hospital initiative (BFHI), mother-baby friendly spaces and support groups.

Conclusion

The evidence suggests that IYCF practices are generally poor in conflict inflicted settings. However, there is potential for improvement by designing effective interventions, responsibly disseminating, monitoring and implementing IYCF guidelines as prescribed by WHO development partners, government and non-government organizations with dedicated funds and investing in capacity development.

Key Words

Breastfeeding, IYCF, Conflict, epidemiology, Barriers, programs, Guidelines

For peer review only

Word Count: 5940 words

STRENGTHS AND LIMITATIONS OF THIS STUDY

1. To our knowledge, this is the first systematic review of infant and young child feeding (IYCF) practices in conflict settings that looks at the evidence on the current practices of breastfeeding and complementary feeding, and assesses specific barriers to adapting optimal IYCF practices. This review also explores the evidence on effective strategies to improve IYCF practices, as well as evidence from implementation guidelines, which suggest the way IYCF should be approached in conflict contexts.
2. The review highlights the sub-optimal IYCF practices in children affected by conflict.
3. The review highlights the evidence derived from strategies/interventions implemented in conflict areas to improve IYCF practices, albeit weak, provides important insights for future approaches to improve IYCF practices.
4. The review is limited by the scarcity of evidence as many programs in conflict contexts are not reported. And even within published reports, very few studies have clearly specified the scale of the intervention, year of conflict, year of 'baseline' data or performed a formal evaluation of the program and its impact on IYCF outcomes.

For peer review only

INTRODUCTION

Optimal infant and young child feeding practices (IYCF) play a critical role in determining the nutritional status, health, growth and development of children, along with improving the health of mothers[1-4]. The current guidelines suggest breastfeeding should to be initiated within the first hour of birth and infants be exclusively breastfed for the first six-months of life, i.e. receive only breast milk, with the exception of oral rehydration syrups solutions and drops/ of vitamins, minerals and medicines [1, 5, 6]. Exclusive breastfeeding (EBF) offers the required nourishments for normal growth and development till six months of age[2]; thereafter safe, timely and nutritionally adequate complementary foods should be added to the diet of infants, along with continued breastfeeding up to two years of age[1, 5, 6].

Children who have been breastfed for longer periods of time tend to exhibit lower odds of infectious morbidity and mortality[7], as infants who are not breastfed have six-fold greater risk of infections related in the first two-months of life when compared to infants that have been adequately breastfed [8]. The current evidence suggests that high-income countries (HICs) practice shorter duration of breastfeeding (<20%) compared to low and middle- income countries (LMICs)[7]. However, even within LMICs, approximately only 37% of infants younger than six-months are exclusively breastfed[7]. Just scaling up and promoting breastfeeding to a universal level could possibly prevent 823,000 annual deaths in children under the age of five [7] and 13.8% of these under two years of age[7]. After six months of age, energy–and–nutrient dense foods that can be easily eaten and digested should be added to infants' diet in order meet their dietary demands [9]. Both breastfeeding and appropriate complementary feeding are pivotal for child growth and the prevention of disease and malnutrition[1]. Breastfeeding coupled with complementary feeding has the potential to reduce mortality among children under the age of five by 19%[5].

During times of armed conflict, vulnerable groups including children bear the greatest negative consequences. The onset of conflict increases death rates by up to 24 times, with adverse effects especially for children under the age of five years [10]. Newborns are specifically at a higher risk of dying if they are poor, exposed to unsafe environments, or if they are within a conflict setting [8]. Armed conflict significantly impacts breastfeeding practices with lower rates of breastfeeding observed in war-torn areas [11]. Before Lebanon's conflict in 2006, approximately 27% of mothers exclusively breastfed for the first four-months of life and after the conflict escalated, the breastfeeding practices were severely affected with most mothers discontinuing breastfeeding altogether or initiating mixed feeding and/or reduced breastfeeding [12]. Similarly, in these war-torn areas, complementary feeding may also become severely eroded and disrupted [1]. This could be attributed to safety, access to sufficient quantity and quality of complementary foods, and also appropriate knowledge of complementary feeding [1]. Often, misconceptions associated with the introduction of solid food results in mothers or caregivers, results in either initiating solid food early or waiting more than required[13]. Suboptimal complementary food intake can result in deterioration of health status of infants and young children, increasing the risk of morbidity and mortality[1].

In conflict settings; breastfeeding and appropriate complementary feeding is of crucial importance because it is regarded as the safest way to protect infants and young children from infections and malnourishment[14]. This can be corroborated by the fact that during emergency situations; mortality rates of artificially-fed infants are greatly elevated in comparison to breastfed babies [8],

For peer review only

as the risk mortality due to diarrhea and other infectious diseases are 20 times higher than infants who have been exclusively breastfed[12]. This is due to prevalent unhygienic conditions coupled with lack of safe water and facilities to sterilize feeding bottles and prepare formula safely. Support for optimal breastfeeding, re-lactation, and timely introduction of complementary foods should be the first choice of intervention during conflict situations to mitigate feeding problems for infants and young children[9] and should not be undermined by inappropriate distribution of Breast Milk Substitutes (BMS)[2, 9, 10]. There is currently no existing comprehensive review for IYCF in conflict settings. The objective of this systematic review is to assess the evidence on IYCF practices, factors associated with IYCF, evidence on the interventions undertaken and guidelines to improve IYCF practices in children under two years of age living in conflict settings.

CONCEPTUAL FRAMEWORK

During armed conflicts, IYCF practices are disrupted due to displacement, casualties, lack of resources, stress, and malnutrition, as well as due to unregulated BMS donations by agencies. We developed a conceptual framework to guide this review and highlighting the IYCF practices and factors responsible for improving IYCF practices in an armed conflict and post-conflict settings (Figure 1). It also focuses on the evidence of current IYCF practices, factors associated with improving IYCF and the evidence from interventions and programs implemented in these settings. We also explored existing implementation guidelines and program recommendations for improving IYCF practices from different agencies working in such settings.

METHODOLOGY

We conducted a systematic review for available published and grey literature, assessing four domains including: Epidemiology (coverage of key IYCF and malnutrition indicators), enablers/barriers (for recommended IYCF practices), interventions/programs (effectiveness in improving IYCF practices) and implementation guidelines to improve IYCF practices in conflict settings.

Eligibility Criteria

We included studies assessing the impact of conflict on IYCF practices and enablers/barriers, interventions/programs and guidelines to promote or facilitate IYCF in conflict settings. We included studies conducted in an armed conflict setting, defined as ‘a political conflict in which armed combat involves the armed forces of at least one state (or one or more armed factions seeking to gain control of all or part of the state) and in which people have been killed by the fighting during the course of the conflict’[15]. We included studies conducted during conflict and within five years of its cessation. Included studies comprised of primary research articles, reports and grey literature and policy and guidelines. We included all studies conducted in conflict settings of low, low-middle and upper-middle income countries classified by World Bank [16], during the period of 1980 to 2018. Additionally, studies conducted in refugee camps of HIC were also included for analysis.

For peer review only

We excluded studies conducted in HICs apart from refugee camps. Clinical studies (exclusively looking at microbiological/laboratory outcomes/screening or diagnostic test evaluations or surgical techniques/outcomes) and mathematical modeling or economic study (with no empirical data/information), systematic and literature reviews were excluded from the study. We also excluded studies conducted in humanitarian emergencies apart from armed conflict.

Data Management

We searched PubMed and CENTRAL using our search strategy constructed by using Medical Subject Headings (MeSH) and key words (Box 1). The bibliographies of relevant systematic reviews and included studies were searched to identify the missing records in the database search. We only included articles in English language. Additionally, a grey literature search was conducted on Google and websites and publications of relevant agencies such as WHO, UNICEF, UNHCR, International Baby Food Action Network, Emergency Nutrition Network, Baby Milk Action, Save the Children, Action Against Hunger and Wellstart International. We also searched for additional data by entering titles of all included studies on Google Scholar and reviewing the first 10 pages to include any relevant missing studies.

Box 1: Search Strategy

("Disasters"[Mesh] OR "Disaster Victims"[Mesh] OR "Natural Disasters"[Mesh] OR mass disaster OR catastrophe* OR crisis OR crises OR war OR "afghan campaign" OR "Armed Conflicts"[Mesh] OR conflict OR conflicts OR war OR avalanche* OR cyclone* OR drought* OR earthquake* OR flood* OR hurricane* OR landslid* OR land slide OR land slides OR mudslid* OR mudslides OR mud slide OR mud slides OR storm* OR tornado* OR tsunami* OR typhoon* OR volcano*) AND ("Refugees"[Mesh] OR refugee OR asylum seeker* OR returnees OR “IDP” OR “IDPs” OR internally displaced person* OR mother* OR “lactating” OR “lactating mother” OR “lactating mothers”) AND (Breastfeed* OR “breast feeding” OR “breastfed” OR “exclusive breastfeeding” OR “human milk” OR “lactation” OR infant feed* OR complementary feed* OR “IYCF” OR “Infant and Young Child Feeding” OR “early initiation” OR “colostrum” OR “breastfeeding practices” OR “breastfeeding promotion” OR “breastfeeding facilitation” OR barriers OR “breast milk collection" OR "breast milk collections" OR "breast milk expression" OR "breast milk expressions" OR "breast pumping" OR "breast pumpings" OR "breastmilk collection" OR "breastmilk collections" OR "breastmilk expression" OR "breastmilk expressions" OR “milk bank” OR “feeding bank”)

After running the electronic search, all records were imported to EndNote software[17] and were de-duplicated prior to title and abstract screening. Two reviewers independently screened titles and abstracts, followed by full text screening. All the discrepancies were resolved after discussion at each stage and a third reviewer was contacted incase two reviewers were unable to reach a consensus. Data was extracted independently by two reviewers from the included studies in an excel sheet after the full text screening. We calculated the median of all IYCF and malnutrition indicators from the studies identified and conducted a descriptive and thematic analysis of included

For peer review only - http://bmjopen.bmj.com/site/about/guidelines.xhtml

studies to explore and synthesize information on the contextual factors and intervention and recommended implementation strategies.

We obtained ethical approval of this study from the Ethical Review Committee of Aga Khan University, Karachi and the National Bioethics Committee, Pakistan. The funding for this review was received from the Family Larsson-Rosenquist Foundation.

Patient and Public Involvement

This research was done without patient involvement. Patients were not invited to contribute to the writing or editing of this document for readability or accuracy.

RESULTS

We identified a total of 56 studies (Figure 2) which were broadly classified into the pre-determined four sections; epidemiology, enablers/barriers, programs/interventions and implementation guidelines.

Epidemiology

Twenty-four studies reported on the prevalence of breastfeeding and the burden of disease in different conflict-affected regions [10, 13, 14, 18-38]. Eighteen were reports [10, 13, 14, 18-24, 26, 28-30, 32, 33, 37, 38], five were cross sectional studies[25, 27, 34-36], and one was a cohort study [31]. Seven studies were conducted in Middle East [13, 14, 19, 21-23, 26], six in Europe [18, 19, 27, 30, 33, 35], five in Africa [18, 20, 25, 31, 32], four in Asia [24, 28, 29, 38] and two reported on prevalence in more than one region which included Africa [10], Asia [10, 37] and Middle East [37]. The included studies collected data through various sources and methods; five studies included data from national assessments done by the WHO and other developmental partners [10, 19, 21, 22, 37], five conducted cross sectional household surveys [26, 27, 33, 34, 36], four collected data from multiple indicator cluster survey [13, 14, 29, 35], four conducted SMART surveys [18, 20, 28, 38], one distributed questionnaires [25], one collected data from national surveillance system registry [31], one through Disease Early Warning System surveillance network [24] and two did not specify [23, 32].

Twenty studies reported on IYCF according to the WHO IYCF indicators which are summarized in Table 1[13, 18-21, 23, 25-38]. These studies analyzed IYCF indicators divided into WHO eight core indicators (early initiation of breastfeeding, exclusive breastfeeding under six months, continued breastfeeding at one year, introduction of solid, semi-solid or soft foods, minimum dietary diversity, minimum meal frequency, minimum acceptable diet and consumption of iron-rich or iron-fortified foods) and seven optional indicators (children ever breastfed, continued breastfeeding at two years, age-appropriate breastfeeding, predominant breastfeeding under six months, duration of breastfeeding, bottle feeding and milk feeding frequency of non-breastfed children) [39]. Using the data extracted from included studies, the median prevalence of early initiation of breastfeeding in conflict-affected areas was 51% (range: 31.3% to 85%), EBF was 25% (range: 5.5% to 77.1%), proportion of children with appropriate introduction of solid, semi-solid or soft foods was 71.1% (range: 40.7% to 98.6%), proportion of children with minimum dietary diversity was 60.3% (range: 9.2% to 79.4%), children ever breast-fed was 92% (range: 62.8% to 98.4%), continued breastfeeding at two years was 29% (range: 9% to 66%), age-

For peer review only

222 appropriate breastfeeding was 43.2% (range: 19.5% to 77.8%), predominant breastfeeding was
223 31.3% (range: 7.1% to 77.3%) and bottle feeding was 58.3% (range: 31.8% to 71.4%).

224 Two studies [25, 27] reported on association of breastfeeding with malnutrition in displaced
225 Bosnian[27] and Saharawi children[25]. Infants were more likely to be malnourished who were never
226 breastfed (Odds Ratio (OR): 1.78, 95% Confidence Interval (CI): 1.26, 2.52) or who were not
227 breastfed for atleast four months (OR: 1.45, 95% CI: 1.02, 2.07) than those who were ever
228 breastfed or were breastfed for four months. Malnutrition persisted among infants who were not
229 exclusively breastfed and infants who were breastfed for less than five to six months were more
230 likely to be malnourished (OR:1.98, 95% CI: 1.01, 7.35) than those who were breastfed for more
231 than six months[27]. After adjusting for diseases, mother's body mass index and child's age;
232 prevalence of underweight and wasting was low among children who were predominantly or
233 exclusively breastfed (Mean Difference (MD): 0.62, 95%CI: 0.10, 1.13 and 0.41, 95% CI: -0.08,
234 0.91 respectively)[25].

For peer review only

Table 1: IYCF practices in conflict settings

	Indicators	Target Population: Country	Setting	Estimates Median (range)	Sample size Median (range)	Scale
**Core Indicators**	Early Initiation of Breastfeeding (n=10)[18, 20, 21, 25, 26, 28, 32, 35, 36, 38]	- IDPs: South Sudan, Ukraine<br>- Refugees: Algeria, Jordan, Kenya, Lebanon, Pakistan<br>- Not Specified: Bosnia-Herzegovina, CAR, Yemen	- Camp: Algeria, Jordan, Kenya, Lebanon<br>- Community: Pakistan, South Sudan, Ukraine, Yemen	51% (range:31.3% to 85%)	357 (range:111-1368)	- Camps: Algeria, Jordan, Kenya<br>- Village: Pakistan<br>- District: Yemen<br>- National: CAR
	Exclusive breastfeeding under 6 months (n=16)[10, 13, 20, 21, 25, 26, 28-30, 32-38]	- IDPs: Bosnia-Herzegovina, Ukraine, Sierra Leone, South Sudan<br>- Refugees: Algeria, Greece, Jordan, Kenya, Lebanon, Pakistan<br>- Not Specified: Afghanistan, Bosnia-Herzegovina, Iraq, Kosovo, Lebanon, Syria, Yemen	- Camp: Algeria, Kenya, Lebanon<br>- Community: Bosnia-Herzegovina, Iraq, Kosovo, Pakistan, South Sudan, Ukraine, Yemen<br>- Community and Healthcare Facility: Jordan	25% (range:5.5% to 77.1%)	432 (range:58-250,000)	- Camps: Algeria, Jordan, Kenya<br>- Village: Pakistan<br>- District: Yemen<br>- National: Kosovo
	Continued Breastfeeding at one years (n=7)[21, 28, 32, 33, 35, 36, 38]	- IDPs: Ukraine<br>- Refugees: Greece, Kenya, Lebanon, Pakistan<br>- Not Specified: Bosnia-Herzegovina, Yemen	- Camp: Kenya, Lebanon<br>- Community: Pakistan, Ukraine, Yemen	57.20% (range:8.5% to 78.2%)	326 (range:38-1368)	- Camp: Kenya<br>- Village: Pakistan<br>- District: Yemen
	Introduction of Solid, Semi Solid or Soft Food (n=6)[25, 32, 33, 35, 36, 38]	- IDPs: Ukraine<br>- Refugees: Algeria, Greece, Kenya, Pakistan<br>- Not Specified: Bosnia-Herzegovina,	- Camp: Algeria, Kenya<br>- Community: Pakistan, Ukraine	71.05% (range:40.7% to 98.6%)	477 (range:111-1368)	- Camps: Algeria, Kenya<br>- Village: Pakistan
	Minimum Dietary Diversity (n=5)[18, 20, 28, 33, 38]	- IDPs: South Sudan<br>- Refugees: Greece, Pakistan<br>- Not Specified: CAR, Yemen	- Community: Pakistan, South Sudan Yemen	60.25% (range:9.2% to 79.4%)	309 (range:148-1055)	- Village: Pakistan<br>- District: Yemen<br>- National: CAR
	Minimum Meal Frequency (n=6)[18, 20, 28, 33, 36, 38]	- IDPs: South Sudan, Ukraine<br>- Refugees: Greece, Pakistan<br>- Not Specified: CAR, Yemen	- Community: Pakistan, South Sudan, Ukraine, Yemen	58% (range:58% to 97.6%)	432 (range:148-1055)	- Village: Pakistan<br>- District: Yemen<br>- National: CAR
	Minimum Acceptable Diet (n=5)[18, 20, 28, 33, 38]	- IDPs: South Sudan<br>- Refugees: Greece, Pakistan<br>- Not Specified: CAR, Yemen	- Community: Pakistan, South Sudan, Yemen	24.95% (range:30.5% to 33%)	309 (range:148-1055)	- Village: Pakistan<br>- District: Yemen<br>- National: CAR

For peer review only

	Consumption of Iron Rich and Iron Fortified Food (n=2)[26, 36]	- IDPs: Ukraine<br>- Refugees: Jordan	- Camp: Jordan<br>- Community: Ukraine	51.61% (range:29% to 84.7%)	379 (range:281-477)	- Camp: Jordan
**Optional Indicators**	Children ever breastfed (n=5)[27, 30, 33, 36, 38]	- All (refugees, displaced and not displaced): Bosnia-Herzegovina<br>- IDPs: Ukraine<br>- Refugees: Greece, Macedonia, Pakistan	- Camp: Macedonia<br>- Community: Bosnia-Herzegovina, Pakistan, Ukraine	92% (62.8% to 98.4%)	766 (range:148-1123)	- Camp: Macedonia<br>- Village: Pakistan<br>- National: Bosnia-Herzegovina
	Continued Breastfeeding at two years (n=6)[21, 29, 33, 35, 36, 38]	- IDPs: Ukraine<br>- Refugees: Lebanon, Pakistan, Greece<br>- Not Specified: Bosnia-Herzegovina, Iraq	- Camp: Lebanon<br>- Community: Iraq, Pakistan, Ukraine	29% (range:9% to 66%)	477 (range:148-55194)	- Village: Pakistan<br>- National: Iraq
	Age appropriate breastfeeding (n=3)[33, 35, 38]	- Refugees: Greece, Pakistan<br>- Not Specified: Bosnia-Herzegovina	- Community: Pakistan	43.20% (range:19.5% to 77.8%)	602 (range:148-1055)	- Village: Pakistan
	Predominant breastfeeding under 6 months (n=5)[25, 30, 33, 35, 38]	- Refugees: Algeria, Greece, Pakistan<br>- Not Specified: Bosnia-Herzegovina, Kosovo	- Camp: Algeria<br>- Community: Kosovo, Pakistan	31.30% (range:7.1% to 77.30%)	176 (range:111-1055)	- Village: Pakistan
	Duration of breastfeeding (n=2)[27, 31]	- All (refugees, displaced and not displaced): Bosnia-Herzegovina<br>- IDPs: Guinea-Bissau	- Community: Bosnia-Herzegovina, Guinea-Bissau	22.7 months	2149 (range:1741-2556)	- City: Guinea-Bissau<br>- National: Bosnia-Herzegovina
	Bottle feeding (n=3)[21, 36, 38]	- IDPs: Ukraine<br>- Refugees: Lebanon, Pakistan	- Camp: Lebanon<br>- Community: Pakistan, Ukraine	58.30% (range:31.8% to 71.4%)	477 (range:174-1055)	- Village: Pakistan
	Milk feeding frequency of non-breastfed children (n=1)[33]	- Refugees: Greece	- Not Stated	33.90%	148	- Not Stated
Malnutrition	Underweight (n=4)[10, 25, 28, 38]	- IDPs: Afghanistan, Sierra Leone<br>- Refugees: Algeria, Pakistan<br>- Not Specified: East Timor, Yemen	- Camp: Algeria<br>- Community: Pakistan, Yemen	33.10% (range:12.01% to 48%)	1055 (range:111-250,000)	- Camp: Algeria<br>- Village: Pakistan<br>- District: Yemen
	Acute Malnutrition (n=3)[10, 24, 36]	- All ((IDPs, refugees and residents): Southern Somalia<br>- IDPs: Ukraine, Pakistan	- Camp: Southern Somalia, Ukraine<br>- Camps, hospitals and mobile clinics: Pakistan	30.10% (range:0.5% to 81%)	80,000 (range:477-3,000,000)	- Camps and Healthcare facilities: Pakistan

For peer review only

	GAM (n=7)[14, 18, 20, 23, 28, 32, 38]	- IDPs: South Sudan<br>- Refugees: Jordan, Kenya, Pakistan<br>- Not Specified: CAR, Yemen	- Camp: Jordan, Kenya<br>- Community: Pakistan, South Sudan, Yemen	9.95% (range:2.6% to 25.1%)	498 (range:208-1368)	- Camp: Jordan, Kenya<br>- Village: Pakistan, Yemen<br>- District: Yemen
	MAM (n=9)[10, 19, 20, 25, 28, 34, 36, 38, 40]<br>(Z- score <-2 to <-3 SD)	- IDPs: Afghanistan, Bosnia-Herzegovina, Sierra Leone, South Sudan, Sudan, Ukraine<br>- Refugees: Algeria, Jordan, Pakistan<br>- Not Specified: East Timor, Yemen	- Camp: Algeria, Ukraine<br>- Community: Bosnia- Herzegovina, Jordan, Pakistan, South Sudan, Sudan, Yemen	4.0% (range:0.27 to 25%)	563 (range:111-250,000)	- Camp: Algeria<br>- Village: Pakistan<br>- District: Yemen<br>- Governorates: Jordan
	SAM (n=8)[18-20, 23, 28, 32, 38, 40]<br>(Z- score <-3 SD)	- IDPs: South Sudan, Sudan<br>- Refugees: Jordan, Kenya, Pakistan<br>- Not Specified: CAR, Yemen	- Camp: Kenya<br>- Community: Jordan, Pakistan, South Sudan, Sudan, Yemen	1.50% (range:0.15% to 5.3%)	809 (range:208-46,383)	- Camp: Kenya<br>- Village: Pakistan, Yemen<br>- District: Yemen<br>- Governorates: Jordan
Chronic Malnutrition	Stunting (n=7)[10, 18, 20, 23, 25, 28, 38]	- IDPs: Afghanistan, Sierra Leone, South Sudan<br>- Refugees: Algeria, Pakistan<br>- Not Specified: East Timor, CAR, Yemen	- Camp: Algeria<br>- Community: Pakistan, South Sudan, Yemen	38.60% (range:13.6% to 53%)	432 (range:111-1055)	- Camp: Algeria<br>- Village: Pakistan, Yemen<br>- District: Yemen
	Overweight (n=1)[38]	- Refugees: Pakistan	- Community	18.1%	1055	- Village
	Anemia (n=1)[38]	- Refugees: Pakistan	- Community	23.2%	1055	- Village
Other Indicators	Diarrhea Prevalence (n=3)[10, 24, 28]	- IDPs: Guatemala, Pakistan<br>- Not Specified: Yemen	- Camps, hospital and mobile clinics: Pakistan<br>- Community: Yemen	30% (range:28.9% - 73.8%)	303 (range:42-563)	- Healthcare facilities: Pakistan<br>- District: Yemen
	Mortality due to Diarrhea (n=3)[10, 34, 38]	- IDPs: Bosnia-Herzegovina, Somalia<br>- Refugees: Iraq, Nepal, Pakistan, Uganda, Zaire<br>- Residents: Eastern DRC	- Camps: Southern Somalia<br>- Community: Bosnia-Herzegovina, Pakistan	39% (range:22.3% to 87%)	100, 000 (range:1055-3,000,000)	- Village: Pakistan

For peer review only

Enablers/ Barriers

Eighteen studies reported on enablers/barriers to IYCF practices in conflict settings (Figure 3) [13, 14, 19-22, 25, 27, 29, 33-37, 41-44]. Of these, 12 were reports [13, 14, 19-22, 29, 33, 37, 41, 43, 44] and six were cross sectional studies [25, 27, 34-36, 42]. Seven studies were conducted in Europe [27, 33-37, 44], five in Middle East [13, 14, 19, 21, 22], three in Africa[20, 25, 42], one in Asia [29], one study was conducted in both Asia and Europe[41], and one failed to report[42]. Nine studies reported on refugees [13, 14, 19, 21, 25, 32, 37, 41, 44], four on internally displaced persons (IDPs) [20, 22, 34, 36], three on non-displaced residents[27, 29, 42]and two failed to report on the target population [35, 43]. Nine studies were conducted in camps [13, 14, 19, 21, 25, 32, 37, 41, 44], seven in community [20, 22, 27, 29, 34, 36, 42] and two did not report [35, 43].

Conflict-related violence

Breastfeeding declined significantly in regions with high levels of conflict-related violence relative to areas which were considered the 'safest areas' in Iraq[29]. A mother residing in dangerous areas of Iraq was 17.4% more likely to stop breastfeeding compared to mothers living in the safe areas [29]. In Bosnia-Herzegovina, illness of mother/baby disrupted breastfeeding and a few mothers made a personal decision not to breastfeed[35]. Other reasons that negatively affected breastfeeding practices in conflict affected areas were unavailability of trained healthcare professionals, and disruption in knowledge created by violent conflict [19, 29].

Misconceptions

Ten studies reported the common misconception amongst refugees, media, and humanitarian workers that mothers produce poor quality, insufficient milk due to malnourishment and stress induced as an impact of war [19-22, 29, 33, 34, 37, 41, 43]. These misconceptions led to mothers feeding undiluted animal milk to infants younger than four months[20]. In Bosnia-Herzegovina and Jordan, health professionals discouraged mothers from breastfeeding due to significant maternal weight loss during conflict [19, 34]. Breastfeeding practices of mothers were affected by mental trauma from fighting and due to war-related casualties in male members of the family [20, 21, 27, 29, 41]. In Bosnia-Herzegovina, infants were less likely to be breastfed for more than 4 months if they resided closer to the area of conflict and if the household was not receiving remittances from abroad [27].

Initiation of breastfeeding

Studies conducted in Algeria, Jordan, and Northern Uganda mentioned factors leading to delayed initiation of breastfeeding by refugee mothers as home delivery, C-section, breast pain, discarding initial milk, illiteracy, lack of support from medical staff, previous experience, mother being ill and being the sole person responsible for decision of initiating breastfeeding [25, 26, 42]. Mothers used pre-lacteal feeds before initiating breastfeeding, as colostrum was believed to be of low nutritional value and was considered 'dirty' and harmful [25, 42]. The report from Iraq and Greece mentioned that many mothers assumed that their milk was 'dirty' if a previous infant died while breastfeeding, resulting in them refraining from breastfeeding the next baby [41].

Introduction of complementary foods

For peer review only

Early introduction of complementary food and infant formula also led to suboptimal breastfeeding practices [13, 21, 33, 35, 43]. They were introduced as early as 1-3 months because of the misconception that breastmilk was not sufficient for meeting the nutritional requirements of the infants [21, 35]. Seven studies focusing mainly on refugees from conflict affected countries, explored reasons for mix feeding or fully artificial feeding infants [13, 19, 21, 33, 35, 41, 43]. Breastfeeding and complementary feeding practices were influenced by religious and cultural determinants and frequent migrations [29, 33]. Studies from Jordan and Bosnia-Herzegovina mentioned about the role of grandmothers in influencing breastfeeding and complementary feeding practices and barriers[19, 35]. Grandmothers often pressurized mothers to feed BMS (water and herbs) to infants and asked mothers to follow their traditional approach. Moreover, there was a cultural belief that formula milk is safer than breast milk and to introduce complementary feeding early [19, 41].

Breast Milk Substitutes (BMS)

Refugees in Lebanon and Greece considered BMS as necessary food for infants during emergencies and many mothers pursued mixed feeding because of the physician's prescription of infant formula[13, 33, 43]. The study on Syrian refugees in Lebanon stated that in children above six months, child refusal of food was due to inadequate knowledge regarding complementary feeding practices and the quantity of solid food that a young child should receive per day [21]. In Azraq refugee camp in Jordan, poor quality food and lack of iron-rich foods in the market was the reason for low consumption of iron-rich foods by the infants and young children[26]. In Bosnia-Herzegovina and Lebanon, mothers introduced tea, sugar, water, juice and infant formula in the feeding practice to spend less time breastfeeding which negatively influenced the rate of EBF[21, 35]. Two studies' mentioned 'lack of milk' as the reason for early weaning by mothers[33, 35], and many mothers wrongly thought that weaning can't be reversed[43]. Another reason for suboptimal breastfeeding practices, mentioned by four studies from Iraq, Greece, Lebanon and Jordan, was the heavy marketing of artificial feeding and big push from infant formula companies, which made mothers believe that it was better[13, 14, 33, 41].

Programs/ Interventions

Breastfeeding in an emergency is known to be the safest way to protect infants and young children from an increased risk of infection and undernutrition [14]. To reduce the burden of disease and poor IYCF practices; several programs were launched in conflict-affected countries by different organizations (WHO, international NGOs and local NGOs). The interventions were mostly based on the WHO guidelines to promote, protect, and support appropriate IYCF practices.

We included fifteen studies which reported on promotion of optimal IYCF practices in conflict settings (Table 2) [10, 13, 14, 18-24, 32, 35, 40, 41, 44]. Fourteen were reports[10, 13, 14, 18-24, 32, 40, 41, 44] and one was a cross-sectional study[35]. These programs were formulated based on literature review, document review and extensive discussions of stakeholders during conferences, through semi-structured interviews (e.g., open group discussions of mothers/ caregivers), and knowledge, attitude and practices surveys. Six studies were conducted in Middle East[13, 14, 19, 21-23], four in Africa [18, 20, 32, 40], three in Europe[10, 35, 44], one in Asia[24] and one study was conducted at more than one place i.e. Europe and Asia[41]. Eight of these focused on refugees[10, 13, 14, 19, 21, 32, 41, 44], three involved IDPs[20, 22, 24], one reported on both hosts and IDPs[40] and three studies failed to report on it [18, 23, 35]. Six studies were conducted in refugee camps[10, 13, 21, 32, 41, 44], five in

For peer review only

community[18, 20, 22, 23, 40], three in both camps as well as in clinics[14, 19, 24], and one study failed to report on it[35].

The specific interventions in these programs included capacity building of healthcare staff, education and awareness activities for mothers, community mobilization, provision of baby friendly spaces, lactation support services, complementary and safe artificial feeding support services, baby friendly hospital initiative (BFHI) and monitoring and control of BMS[10, 13, 14, 18-24, 32, 35, 40, 41, 44]. Five studies focused only on one intervention for promotion of optimal breastfeeding practices [22-24, 35, 44], while rest had a multi-pronged approach [10, 13, 14, 18-21, 32, 40, 41]. Most of the studies/programs did not evaluate the programs and its impact on IYCF practices or health and nutrition indicators. A follow-up survey after three months of intervention, assessed breastfeeding practices among Syrian refugees in Jordan, which showed an increase in breastfeeding knowledge from 49.5% in 2013 to 71% in community and 91.2% in health facility in 2014. However, no improvement in breastfeeding practices was observed[19]. Similarly, in Greece, formula use in the refugee camps decreased from 60% to 0% within 6 months, through their IYCF program in emergencies [41].

For peer review only

Table 2: Programs/ Interventions to Promote Optimal IYCF Practices

Program	Target Population/ Countries	Setting	Health Workforce Involved	Program/ Intervention Details	Outcomes
Capacity Building and Program-Strengthening Projects for Health Workers (n=7)[13, 14, 19, 22, 32, 40, 41]	- IDPs: Sudan, Syria<br>- Refugees: Jordan, Kenya, Lebanon<br>- Not Specified: Greece, Iraq	- Camps: Jordan<br>- Fixed and mobile clinics: Jordan, Lebanon	- Trained Physician (doctors, nurses)<br>- Paramedic Staff (midwives, TBAs)<br>- Community Workers (facility and community based IYCF counsellors, local reproductive health worker)	- Training sessions on IYCF<br>- Malnutrition screening and treatment<br>- Continuous follow up and co-ordination	**Coverage of Training**:<br>- In Lebanon and Syria: >190 doctors and health workers[27, 29]
Education and Awareness Activities for Mothers (n=7)[13, 14, 18, 19, 21, 24, 32]	-IDPs: Pakistan<br>-Refugees: Jordan, Kenya, Lebanon<br>-Not specified: CAR	- Camps: Jordan, Kenya, Lebanon, Pakistan<br>- Community: CAR, Lebanon<br>- Fixed and mobile clinics: Jordan, Pakistan<br>- Healthcare facility: Jordan Pakistan	- Trained Physician (lactation specialists)<br>- Community Workers (Nutrition officer, IYCF educator and a community mobiliser)	- Counselling sessions on optimal IYCF-E practices<br>- Educational materials and counselling cards distributed<br>- Sensitizations on childcare practices and cooking demonstrations given to PLW<br>- Distribution of hygiene and baby kits (soaps, blankets, baby spoons, and cups for children), and bangles for mothers	**Coverage of IYCF Counselling**<br>- In Jordan: 30-40 mothers counselled/day[9]; 4,690 PLWs and 919 mothers counselled in 10 months[17]<br>- In Lebanon: 10,000 mothers were counselled in one year[27]<br>- In CAR: 758/900 (84.2%) PLW participated[16]
Community Networking and Mobilization (n=8)[13, 14, 19, 20, 23, 32, 40, 41]	- IDPs: South Sudan, Sudan<br>- Refugees: Jordan, Kenya, Lebanon<br>- Not Specified: Greece, Iraq, Yemen	- Camps: Jordan, Kenya<br>- Community: Lebanon, South Sudan, Sudan, Yemen	- Community Workers (refugee mothers, community leaders, 'leader mothers' (mothers trained by promotors to teach neighbor women), and 'neighbor women' (chosen by community) as community mobilisers)	- Training of community mobilizers by IYCF counsellors, educators, 'promotors', program supervisors and IYCF coordinators on IYCF and the importance of exclusive breastfeeding, nutrition<br>- Dissemination of messages among mothers through household visits, demonstrations and information sharing within care groups<br>- Screening and referring malnourished mothers	**Coverage of Health Workers Training:**<br>- In South Sudan, 320 'leader mothers' trained IYCF[18]<br>**Coverage of IYCF Counselling**<br>- In Jordan: 4,977 PLWs and 31,485 caregivers in 10 months[17]<br>- In South Sudan: 320 neighborhood groups, reaching 3,832 women[18]<br>- In Yemen, 50-60% increase in awareness of mothers/caregivers on nutritious food along with an increase in utilization of local foods for preparing nutritious meals for infants[31]<br>**Mass Screening and SAM/MAM Treatment**<br>- In Yemen: 90% of children <2 years screened for SAM and MAM, 2,563 children were treated for SAM, reduction in number of cases of SAM and MAM children; zero cases of SAM (MUAC <115mm) in 13/68 model villages by the end of the project[31]<br>- In Sudan: 150,617 children screened for malnutrition[43]<br>- In Yemen: Reduction in bottle feeding to almost zero[31]
Mother Baby Friendly Spaces and Mother to Mother Support	- IDPs: South Sudan, Sudan	- Camps: Albania, Kenya, Lebanon<br>- Caravans: Jordan	- Trained Physician: Pediatrician, lactation consultant<br>- Community Workers: Relief workers, psychosocial workers, pediatrician,	- Construction of Mother-baby friendly spaces, caravans and mother baby center for counselling and 24-hour support	**Coverage of IYCF counselling in baby friendly spaces and mother-to- mother support groups:**<br>- In Jordan: 15,600 mothers in 18 months in Jordan[9]<br>- In Kenya: 581 facilitators trained in IYCF[36]

For peer review only

Groups (n=10)(10, 13, 14, 18-21, 32, 40, 44)	- Refugees: Albania, Croatia, Jordan, Kenya, Lebanon<br>- Not Specified: CAR	- Community: South Sudan, Sudan<br>- Containers: Croatia<br>- Primary healthcare centers: Lebanon	lactation consultant, IYCF counsellors, and community mobilisers	- Formation of mother to mother/caregiver support groups<br>- Distribution of high energy biscuit, a bottle of water, and shawls for privacy<br>- Distribution of food vouchers to mothers for nutritional security and psychological support<br>- Screening of children for malnutrition	- In CAR: 199 mothers/ caregivers given psychosocial support[16]<br>**Other Outcomes:**<br>- In Jordan: 120-150 mothers/day visited baby friendly spaces in Jordan[9]<br>- In Jordan: 50 women attended support group gatherings[17]<br>- In Sudan: 14,272 infant/ mother pairs attended MtMSG[43]<br>- In Kenya: 713 MtMSG (2801 mothers/caregivers in MtMSG)[36]
Lactation Support Service (n=3)(10, 13, 19)	- Refugees: Albania, Jordan, Lebanon,	- Camps: Albania, Jordan<br>- Community: Lebanon	- Trained Physician (Lactation specialists, obstetrician/gynecologist)<br>- Paramedic Staff (midwives)	- Assist mothers for re-lactation and in breastfeeding difficulties (painful nursing, latching problems, low breast milk production, and on correct positioning for feeding)	**Coverage of counselling:**<br>- In Lebanon: 3,150 mothers in 6 months[27]
Baby Friendly Hospital Initiative (n=2)(13, 35)	- Refugees: Lebanon<br>- Not Specified: Bosnia-Herzegovina	- Camps: Lebanon	- Not Specified	- Labelling of maternity wards as "baby-friendly" to support breastfeeding<br>- Capacity building of health workforce<br>- Provision of tools and equipment to support breastfeeding, reducing use of BMS	In Bosnia-Herzegovina, from 1997-1999:<br>- Predominant breastfeeding increased from 64.3% to 77.3%<br>- Continued breastfeeding at 2 years increased from 8.5% to 40.7%
Breast Milk Substitutes (n=5)(13, 14, 19, 21, 41)	- Refugees: Jordan, Lebanon<br>- Not Specified: Greece, Iraq	- Camps: Greece, Iraq, Jordan and Lebanon	- Trained Physician (lactation specialists, and obstetrician/ gynecologist)<br>- Paramedic Staff (IYCF midwife)	- Training of healthcare staff and mothers on artificial feeding<br>- Counselling of mothers on importance of breastfeeding, appropriate use of infant formula and on adverse effects of artificial feeding on infant's health<br>- Monitor and control the distribution of infant formula- - Provision of BMS supplies and kits (cups and clean water) for safe preparation of infant formula	- In Lebanon: 50 infants were assisted with artificial feeding support[27]<br>- In Jordan: Seven mothers received artificial milk supplies[9]
**Key:** Breast Milk Substitutes (BMS); Internally Displaced Persons (IDPs); Infant and Young Child Feeding (IYCF); Infant and Young Child Feeding in Emergencies (IYCF-E); Traditional Birth Attendants (TBAs), Pregnant and Lactating Women (PLWs); Exclusive Breastfeeding (EBF); Central African Republic (CAR), Mother to mother support groups (MtMSG)					

For peer review only

Implementation Guidelines

We included 30 implementation guidelines by different organizations in our review, mentioning key policies and operational guidelines to be followed during conflicts to ensure optimal IYCF practices [1, 2, 6, 26, 30, 33, 43, 45-68]. Eight guidelines were by international NGOs[1, 30, 33, 43, 46, 51, 59, 67] nine by UN agencies[6, 47, 50, 53, 55-57, 65, 66], and three by academic organizations[48, 49, 64], while ten guidelines were formulated by collaboration between developmental partners and international NGOs[2, 26, 45, 52, 54, 58, 60-63]. These implementation guidelines were formulated after conducting random cluster surveys (including quantitative and qualitative analysis), meetings to gathering empirical evidence, past emergency experiences, and technical guidance from community, stakeholders, development and implementation partners (appendix table 1).

We included implementation guidelines from 1981 through 2018. Only one guideline was from 1981-1990[50], seven were from 1991-2000[30, 47, 51, 53, 54, 66, 67], 12 were from 2001-2010[2, 6, 43, 46, 48, 49, 52, 55, 56, 62, 63, 68], and ten were from 2011-2018[1, 26, 33, 45, 57-59, 61, 64, 65]. These guidelines focused on refugees and IDPs affected by conflict. We summarized these operational guidelines from different organizations, to be used during conflict settings, using the components from 'WHO's guiding principles for feeding infants and young children during emergencies'[6] (Figure 4).

Breastfeeding

The guidelines on protecting, promoting and supporting breastfeeding were mentioned by 28 publications [1, 2, 6, 26, 30, 33, 43, 45-55, 57-67]. They recommend to practice EBF till six months of age, followed by frequent breastfeeding until two years, along with complementary feeding to protect babies from infection, especially in crisis situations. There should be formation of practical guidelines, capacity building of healthcare staff, and continuous flow of funds for the sustainability of programs.

General population (including mothers) should be educated on breastfeeding, its benefits, colostrum use, feeding methods, and consequences of artificial feeding based on 'UNICEF/WHO Baby friendly Hospital's initiative's Ten Steps to breastfeeding'. Moreover, mothers should be counselled not to stop breastfeeding in emergency situations, sickness, and when malnourished. They should be encouraged to practice skin-to-skin contact, initiate breastfeeding within the first hour of birth, and use of pacifiers, artificial nipples and estrogen containing contraceptive pills should be discouraged.

Guidelines also recommend formation of baby friendly spaces and mother to mother support groups to encourage breastfeeding and privacy. There should be a system set in place for identification of newly arriving mothers and children, registration for rations and referral for immediate assistance. Mothers should be facilitated for re-lactation if separated from the baby or experiencing difficulties in breastfeeding. When natural breastfeeding is not possible, wet-nursing and the use of milk banks should be considered before providing infant formula or home-modified milk.

Breast-Milk Substitutes (BMS)

Twenty-six guidelines reported on the use of BMS in emergency situations [1, 2, 6, 30, 33, 43, 45-55, 57-62, 64, 65, 67]. Donations, procurement, promotion (through advertisements and gifts) and distribution of

BMS, bottles and teats should be strictly controlled and should comply with the International Code and World Health Assembly regulations, and all violations should be reported (see Box 2). The distribution should be managed by a single designated agency and blanket distribution of BMS should be discouraged in emergency situations, especially where hygienic conditions can't be ensured.

BMS are not recommended for children under 6 months of age and should only be distributed to infants who have no other viable breast milk options i.e. orphans, severe maternal illness or malnutrition (based on established criteria- where distribution can be targeted, the supply chain is secure, and the conditions for safe preparation and use can be met), determined by a qualified health worker trained in IYCF. There should be training of workforce handling BMS and demonstrations should be given to mothers on proper and safe use of infant formula, followed by regular infant health and growth monitoring. Availability of fuel, safe water and equipment should be ensured before BMS distribution and mothers should be advised to use cups instead of bottles for feeding. Moreover, it should be ensured that distribution of BMS to the targeted infant continues for as long as the infant needs (at least 6 months).

Infant formula (generic, unbranded formula) should be distributed with labels in the local language to the infants in need. These labels should have clear instructions on its safe preparation, along with stating the importance of breast milk. Infant formula should not be excluded from the commodities that a mother can access via cash/voucher schemes, but it should have clear information on superiority of breastfeeding. For infants under six months of age, the only suitable BMS is infant formula and condensed milk should not be used for infant feeding and home-modified milk should be the last resort.

Relief workers should ensure that milk products are received and distributed in a dry form and dried milk products are distributed only when pre-mixed with a milled staple food and should not be distributed as a single commodity. Moreover, it should be ensured that dried skimmed milk is not given to infants and for older children, it should be given after fortifying it with vitamin A. Relief and healthcare workers should counsel mothers to avoid baby juices and teas, and they should take appropriate measures to reduce spill-over by ensuring that feeding BMS to a minority of children doesn't undermine breastfeeding practices of the majority. In case of unfavorable circumstances for BMS distribution, an on-site supplementary "wet" feeding program should be conducted in closed spaces under supervision.

413

Box 2: Violation of IYCF-Emergency guidelines in conflict settings (Case Studies)
**Case Study 1: Campaigning by Blédina company during Hezbollah–Israel Conflict, 2006: Lebanon**<br><br>A marketing campaign by Bledina (production company of infant formula and complementary foods), after the war in 2006 in Beirut, offered mothers gift packages containing promotional leaflets. The attending pediatrician also gave mothers a card with the Blédina hotline. Moreover, free Blédina gift packages were also distributed at a health center which had a high number of refugees, as a promotional campaign after the war, which was a violation of the IYCF-Emergency guidelines [12, 46].
**Case Study 2: Civil Unrest, 2002: East Timor**<br><br>Healthcare facilities in Dili distributed infant formula donated by a service organization to assist infants who have been orphaned and mothers who are unable to breastfeed, which was a violation of the guidelines because health care facilities should not distribute infant formula as it can be wrongly interpreted as them promoting the products, even if they are doing so to meet the needs of the targeted population [46].<br><br>**Case Study 3: Israel-Hezbollah conflict, 2006: Lebanon**<br><br>1. During the Lebanon conflict, an NGO violated the IYCF-Emergency guidelines by distributing 1,500 'baby kits', including infant formula and bottles, directly to displaced households, as well as to hospitals and local municipalities. Even after the conflict, the same NGO distributed 'village kits' containing infant formula (25 boxes containing 24 cans each) and baby food (80 units) along with other items to each village municipality [12, 46].<br>2. Another violation was the donation of infant formula to the healthcare facilities by an international NGO during the Lebanon conflict [12].<br>3. Moreover, one local NGO distributed the infant formula amongst the displaced population, without clearly specifying on the tins that it should only be used on the advice of a health worker thus violating the labelling requirements, and promoting infant formula use [12].<br>4. Another violation occurred when single tins/samples of infant formula were distributed to mothers by health workers, without the undertaking that the infant formula supplies would continue for as long as the concerned infant needs them [12].
**Case Study 4: Israel-Hezbollah conflict, 2006: Lebanon**<br><br>1. The formulas received by Lebanese government organizations and NGOs had labels written in English and/or Greek, instead of the local language, Arabic [12, 46].<br>2. One local NGO distributed infant formula, advertising and promoting artificial feeding, which was in violation of labelling requirements [12, 46].

414

415 Complementary Feeding

Eighteen guidelines reported on complementary feeding in infants and young children 6-24 months of age [1, 6, 26, 43, 45, 48, 49, 51, 54, 55, 57-59, 61-65]. For normal growth and development of infants and children (>6 months), easily digestible complementary foods should be started along with continuation of breast milk. Discussions should be conducted in groups, and mothers should be encouraged to increase the frequency and variety of complementary food with the growing age of child to meet their nutritional demands. Mothers should be encouraged to use locally produced, inexpensive complementary foods. However, in emergency situations, micro-nutrient fortified blended foods, ready-to-use supplementary foods, lipid based nutrient supplements or ferrous sulphate iron solution (iron drops) can be used depending on nutritional situation. Relief workers should ensure that complementary food products are labelled in local language with instructions on preparation and do not have the images of bottle feeding on them, and donations of complementary foods, baby teas or juices should be refused. For children over the age of 12 months, it is recommended that they should eat the same food as older children. If safe complementary foods are not available, mothers should be advised to continue breastfeeding.

Caring for Caregivers and Protecting Children

Thirteen guidelines reported on caring for caregivers during emergencies [1, 6, 33, 45, 52, 58, 60-62, 64-67] and nine reported on protecting children[1, 33, 45, 48, 49, 52, 57-59, 61, 62, 64, 67]. Guidelines recommend provision of psychological support and empowering mothers during crisis and advising them to continue breastfeeding (via milk expression). It should be ensured that infants are screened for childhood illnesses and mothers of artificially-fed infants have access to cleaning facilities for washing utensils for safe preparation of BMS as artificially-fed infants are known to be at a greater risk of malnutrition, diarrhea and chest infections. If an infant is ill with reduced appetite, mothers should continue breastfeeding with smaller amounts and increased frequency. Relief staff should prioritize infants for re-lactation, re-establishment of exclusive breastfeeding and BMS provision and associated support services.

Malnutrition

Nine guidelines reported on management of malnutrition in infants and young children during emergencies [1, 6, 52, 54, 55, 58, 60, 65, 67]. Guidelines recommend continuous monitoring of nutritional status of mothers, infants and young children with the purpose of identifying, assessing, preventing and treating malnourished mothers and children. Malnutrition treatment and prevention programs should incorporate and prioritize IYCF in their agenda as the risk can be decreased with optimal IYCF practices. Education should be provided to community support networks on the prevention and treatment of acute malnutrition and cash/voucher programs should be introduced. For prevention of malnutrition, micronutrients should be distributed to all pregnant and lactating women in form of powders and tablets. It is recommended to identify malnourished children through regular monitoring and they should be referred and admitted along with their mothers to a nutritional rehabilitation program in case of severe malnourishment. In crisis situations, supplementary feeding should be the primary strategy for prevention and treatment of moderate acute malnutrition and pre-formulated therapeutic milk products or dried skimmed milk can be used to treat severe acute malnutrition.

The Acute Phase of Emergencies

For peer review only - http://bmjopen.bmj.com/site/about/guidelines.xhtml

Twelve guidelines reported on interventions to be undertaken during the acute phase of emergencies [1, 2, 6, 43, 45, 51, 55, 58, 61, 62, 64, 67]. In case of an emergency, interventions should start immediately with focus on capacity building to improve IYCF practices, supporting caregivers and catering to nutritional needs of children in order to minimize the negative impact of emergency. In places with prior high infant formula use, appropriate interventions should be undertaken by relevant organizations to increase prevalence of appropriate IYCF practices and increase the rate of EBF and measures should be taken to monitor BMS donations and distribution during early phases of emergencies. Moreover, mothers should be educated and encouraged to breastfeed every 2-3 hours at breastfeeding stations scattered across refugee sites. If mothers are experiencing difficulty in breastfeeding, chlorpromazine can be used to stimulate milk production and wet-nursing and milk banks can also be used as an alternative to BMS.

Assessment, Intervention and Monitoring

Fifteen guidelines mentioned assessment, intervention and monitoring during emergencies [1, 2, 6, 26, 45, 51, 52, 57-61, 64, 65, 67] . There should be regular systematic monitoring to track BMS distribution and careful monitoring of optimal feeding and nutritional status of infants and young children. It is recommended to conduct weight monitoring, assess intake, urination frequency, and activity level for those receiving BMS. Healthcare staff should use qualitative as well as quantitative methods to gather data regarding pre-crisis practices, demographics, morbidity, mortality, malnutrition and current IYCF practices.

Breastfeeding and HIV

Ten guidelines reported on breastfeeding in HIV situations [1, 45, 47, 55-58, 60, 63, 67]. Guidelines recommend that appropriate measures should be undertaken to prevent mother-to-child transmission of HIV, and improve child survival from HIV. If the mother's HIV status is negative or unknown (or HIV testing is not available), she should be advised to continue age appropriate breastfeeding and replacement feeding should only be supported if is acceptable, feasible, affordable, sustainable and safe. For sustainable access to medical care, HIV positive mothers should be provided with antiretroviral treatment and even in case of unavailability, breastfeeding should be continued.

If HIV-positive mothers choose not to breastfeed the infant, appropriate BMS should be provided along with counselling on the risks of mixed feeding and artificial feeding. Wet nursing should also be considered in cases of HIV-positive mothers and wet nurse should be counseled on prevention of disease transmission. IYCF staff should make supportive arrangements for HIV-positive mothers to build confidence, reduce isolation, encourage age appropriate feeding and educate the family members to provide full support to the mothers and conduct regular follow ups. Furthermore, activities related to Prevention/Elimination of Mother-to-Child Transmission should be regularly done as part of nutritional interventions and the measures taken by ICYF staff should be sensitive.

DISCUSSION

This review included a total of 56 primary studies, comprising of 11 published articles and 45 reports from grey literature. The included studies covered IYCF practices, programs and guidelines for the countries affected by armed conflict. The review shows that the coverage of IYCF practices are low in conflict settings; with only half of the children receiving early initiation of breastfeeding and only

a quarter being exclusively breastfed. The other IYCF indicators are also not encouraging, with high rates of bottle-feeding.

The low IYCF indicators can be attributed to a multitude of factors in conflict settings which may also exist in non-conflict settings, though armed conflicts tend to aggravate and amplify these and there are also factors unique to conflict settings. Displacement, stress, maternal malnourishment, lack of awareness, and unavailability of trained healthcare professionals, are all reasons contributing to poor IYCF. The death of the male members within the family also poses an additional barrier, as apart from increased maternal mental stress, it also leads to additional maternal responsibilities. This in turn may compromise the mothers' ability to provide attention to their children. The review highlights certain misconceptions in the community that contribute to the misguided belief that maternal malnourishment and stress lead to insufficient quantity and quality of breast milk and may stimulate the undesired use of BMS. Additionally, in some instances health workers due to their lack of awareness and knowledge, advocated for and prescribed BMS resulting in the unregulated marketing, provision and distribution of BMS.

The evidence from this review suggests that there is a need to enhance the capacity of health workers and to improve their communication with the community by using various channels including pictorials, videos or face to face meetings. Though various programs have been initiated in conflict settings[69]; unfortunately, none of them have been formally evaluated to gauge the impact of these different approaches on IYCF indicators. As shown in the review, the major evidence and recommendations derived from the experiences of the various implementation agencies working in such contexts rather than from scientific evaluation of programs. These guidelines suggest that first and foremost, the importance of IYCF should be underscored and it should be a top priority for improving the health of children in conflict settings. There should be early dissemination of policies to all concerned agencies and healthcare workers. And steps should be taken to register women and mothers in camps, as IDPs or as residents of conflict-inflicted areas. The educational approaches should be drafted with specific messages to alleviate the context-specific misconceptions within the community. There is evidence from various communications platforms which could be utilized to spread relevant messages including women support groups, involving prominent community members, designing pictorials, brochures or videos. The need for designated places like feeding tents was also emphasized as these could provide personal spaces for women to feed their children, seek support from peers and also use them as avenues for skin to skin care for preterm and low birth-weight infants. The provision of clean hygienic utensils and safe drinking water should also be ensured for preparing complementary feeds. The guidelines also emphasize the need for strengthening BFHI in the functioning health facilities. Apart from access to required healthcare, mothers should also be provided with lactation and psychological support. The factors identified which negatively affect IYCF practices include, high turnover rate of health workers, lack of funds, poor multi-sectoral coordination, poor monitoring and evaluation system, more focus on malnutrition treatment than prevention, strengthened marketing efforts of BMS by industries, and poor capacity at community level.

The additional approaches identified included 'wet nurse' and 'milk banks' which could be sought after confirmation from specialists solely for mothers who were unable to breastfeed. BMS should be treated as the last resort[3]and efforts should be put in place that allow for stringent regulatory checks on BMS, nipples and pacifiers [3, 70]. All of the commodities in a conflict setting should flow through a common medium and designated agency/agencies, who should be responsible for controlling

For peer review only - http://bmjopen.bmj.com/site/about/guidelines.xhtml

donations and distributing appropriately labeled infant formula, and any violation to these should be reported with timely action taken.

The major strength of our review is that it systematically looks at various areas of IYCF which includes current IYCF practices, specific barriers and strategies to improve breastfeeding and complementary feeding using evidence from implementation guidelines. The limitations of our review include restricted access to studies conducted by various NGOs/ agencies as most of them do not report and inclusion of articles was limited to English language only and only three studies were included from conflict-affected countries in the Asian region. Some of the included studies missed on reporting important information like reporting on study context (e.g. year and scale of conflict/surveys), outcomes, process indicators and program impact. Hence, there is a critical need of further research on the process of implementation, effectiveness of IYCF interventions and cost effectiveness of these interventions in conflict settings.

To ensure effective scale-up of interventions for promotion of IYCF in conflict settings, the emphasis of the stakeholders should be on advocacy and implementation of evidence-based context specific interventions. A multi-sectoral approach together with stringent monitoring and evaluation mechanisms should be in place with capacity building and accountability.

For peer review only

LIST OF ABBREVIATIONS

BFHI	Baby Friendly Hospital Initiative
BMS	Breast-Milk Substitutes
EBF	Exclusive Breastfeeding
HIC	High Income Countries
HIV	Human Immunodeficiency Virus
IYCF	Infant and Young Child Feeding
LMIC	Low- and Middle-Income Countries
MESH	Medical Subject Headings
NGO	Non-Governmental Organization
SMART	Standardized Monitoring and Assessment of Relief and Transition
UN	United Nations
UNHCR	The United Nations High Commissioner for Refugees
UNICEF	United Nations International Children's Emergency Fund
WHO	World Health Organization

For peer review only

DECLARATIONS

Ethical Approval

We obtained ethical approval of this study from the Ethical Review Committee of Aga Khan University, Karachi and the National Bioethics Committee, Pakistan.

Consent for Publication

Not Applicable

Acknowledgements

None

Competing Interests

All the authors declare no conflict of interest. The corresponding author is the overall guarantor and affirms that the manuscript is an honest, accurate, and transparent account of the study.

Data Sharing

The datasets used for analysis in this study are available from the corresponding author on reasonable request. As this is a systematic review, dissemination to the groups is not applicable.

Funding

The support of the Family Larsson-Rosenquist Foundation is greatly acknowledged. The funders had no role in the findings and writing of the manuscript.

Authors' Contributions

ZAB and JKD conceived the idea of the review. ZAP developed the search strategy; AR, ZAP and FAS conducted the search and data extraction with specific inputs from JKD. AR, ZAP and JKD developed the first draft of the paper. ZAB and JKD reviewed and finalized the final manuscript.

ORCID ID

Zahra A. Padhani: https://orcid.org/0000-0003-4777-7565

Faareha A Siddiqui: https://orcid.org/0000-0001-9623-890X

For peer review only

REFERENCES

1. ACF-International. Infant and Young Child Feeding in Emergencies. 2015 December 2015.
2. Borrel A, Taylor A, McGrath M, Seal A, Hormann E, Phelps L, et al. From policy to practice: challenges in infant feeding in emergencies during the Balkan crisis. Disasters. 2001;25(2):149-63.
3. Campbell S. Global challenges in protecting and promoting breastfeeding. primary health care. 2008;18(1).
4. Sinha B, Chowdhury R, Sankar MJ, Martines J, Taneja S, Mazumder S, et al. Interventions to improve breastfeeding outcomes: a systematic review and meta-analysis. Acta Paediatrica. 2015;104:114-34.
5. Essential nutrition actions: mainstreaming nutrition through the life-course. Geneva: World Health Organization; 2019. Licence: CC BY-NC-SA 3.0 IGO.
6. WHO. Guiding principles for feeding infants and young children during emergencies. 2004.
7. Victora CG, Bahl R, Barros AJD, França GVA, Horton S, Krasevec J, et al. Breastfeeding in the 21st century: epidemiology, mechanisms, and lifelong effect. The Lancet. 2016;387(10017):475-90.
8. Davanzo R. Newborns in adverse conditions: issues, challenges, and interventions. J Midwifery Womens Health 2004;49(4 Suppl 1):29-35.
9. Seal A, Taylor A, Gostelow L, McGrath M. Review of policies and guidelines on infant feeding in emergencies: common ground and gaps. Disasters. 2001;25(2):136-48.
10. Bustreo F, Genovese E, Omobono E, Axelsson H, Bannon I. Improving child health in post-conflict countries: Can the World Bank contribute. The World Bank. 2005.
11. O'Connor ME, Burkle FM, Jr., Olness K. Infant feeding practices in complex emergencies: a case study approach. Prehosp Disaster Med. 2001;16(4):231-8.
12. Maclaine A. Infant feeding in emergencies: experiences from Lebanon. 2007. Contract No.: 37.
13. Darjani P, Berbari LS. Infant and young child feeding support in Lebanon: strengthening the national system. ENN [Internet]. 2014 Nov [cited 2019 Apr 17]; (48):20. Available from: https://www.ennonline.net/fex/48/infant.
14. Alsamman S. Managing infant and young child feeding in refugee camps in Jordan. ENN [Internet]. 2014 Nov [cited 2019 Apr 17]; (48):85. Available form: https://www.ennonline.net/fex/48/managinginfant.
15. Ploughshares P. Armed Conflict 2016 [Available from: http://ploughshares.ca/armed-conflict/defining-armed-conflict/.
16. Bank W. New country classifications by income level: 2018-2019 2018 [Available from: https://blogs.worldbank.org/opendata/new-country-classifications-income-level-2018-2019.
17. Analytics C. Endnote Software [Available from: https://endnote.com/.
18. Dozio E, Peyre L, Morel SO, Bizouerne C, Black. Integrated psychosocial and food security approach in an emergency context: Central African Republic. Intervention: Journal of Mental Health and Psychosocial Support in Conflict Affected Areas. 2016;14(3):257-71.
19. Fander G, Frega M. Responding to nutrition gaps in Jordan in the Syrian Refugee Crisis: Infant and Young Child Feeding education and malnutrition treatment. ENN [Internet]. 2014 Nov [cited 2019 Apr 17]; (48): 82. Available from: https://www.ennonline.net/fex/48/responding.
20. Ndungu P, Tanaka J. Using care groups in emergencies in South Sudan. ENN [Internet]. 2017 Feb [cited 2019 Apr 17]; (54): 95. Available from: https://www.ennonline.net/fex/54/emergenciesinsouthsudan.

For peer review only

21. Seguin J. Challenges of IYCF and psychosocial support in Lebanon. ENN [Internet]. 2014 Nov [cited 2019 Apr 17]; (48): 24. Available from: https://www.ennonline.net/fex/48/challenges.
22. Khudari WHO response to malnutrition in Syria: a focus on surveillance, case detection and clinical management. ENN [Internet]. 2014 Nov [cited 2019 Apr 17]; (48): 118. Available from: https://www.ennonline.net/fex/48/whosyria.
23. Sallam AK, Albably K, Zvandaziva C, Singh K. Community engagement through local leadership: Increasing access to nutrition services in a conflict setting in Yemen. ENN [Internet]. 2018 Jan [cited 2019 Apr 17]; (9):10. Available from: https://www.ennonline.net/nex/9nutserviceaccessyemen.
24. Bile KM, Hafeez A, Kazi GN, Southall D. Protecting the right to health of internally displaced mothers and children: the imperative of inter-cluster coordination for translating best practices into effective participatory action. Eastern Mediterranean Health Journal. 2011;17(12):981-9.
25. Aakre I, Lilleengen AM, Aarsand ML, Strand TA, Barikmo I, Henjum S. Infant feeding practices in the Saharawi refugee camps Algeria, a cross-sectional study among children from birth to six months of age. International breastfeeding journal. 2016;12(1):8.
26. Amelia Reese Masterson, Shiromi M. Perera, Moghames P. Camp-based Barrier Analysis of Early Initiation of Breastfeeding, Iron-rich Food Consumption, and Early Antenatal Care Seeking Behaviors of Syrian Refugees in Azraq Camp, Jordan. International Medical Corps

USAID; 2016.
27. Andersson N, Paredes-Solis S, Legorreta-Soberanis J, Cockcroft A, Sherr L. Breast-feeding in a complex emergency: four linked cross-sectional studies during the Bosnian conflict. Public health nutrition. 2010;13(12):2097-104.
28. Children St. Emergency WASH & Nutrition for conflict affected people in Yemen – Taiz governorate. 2017.
29. Diwakar V, Malcolm M, Naufal G. Violent Conflict and Breastfeeding: The Case of Iraq. 2017.
30. Hunger AA. Infant Feeding and Weaning Report 2000.
31. Jakobsen M, Sodemann M, Nylen G, Bale C, Nielsen J, Lisse I, et al. Breastfeeding status as a predictor of mortality among refugee children in an emergency situation in Guinea-Bissau. Trop Med Int Health. 2003;8(11):992-6.
32. Mary S Lung'aho, Stone-Jiménez M. Mother-to-Mother Support Groups in the Dadaab Refugee Camps.
33. Prudhon C. Assessment of Infant and Young Child Feeding Practices among Refugees on Lesvos Island, Greece 2016.
34. Robertson A, Fronczak N, Jaganjac N, Hailey P, Copeland P, Duprat M. Nutrition and immunization survey of Bosnian women and children during 1993. Int J Epidemiol. 1995;24(6):1163-70.
35. Šumanović-Glamuzina D. Breastfeeding practice in Bosnia and Herzegovina. Journal of Pediatric and Neonatal Individualized Medicine (JPNIM). 2013;2(1):55-62.
36. Summers A, Bilukha OO. Suboptimal infant and young child feeding practices among internally displaced persons during conflict in eastern Ukraine. Public Health Nutr. 2018;21(5):917-26.
37. Theurich MA, Grote V. Are commercial complementary food distributions to refugees and migrants in Europe conforming to international policies and guidelines on infant and young child feeding in emergencies? Journal of Human Lactation. 2017;33(3):573-7.

For peer review only

38. UNHCR. UNHCR Nutrition Survey among Afghan Refugees Residing in Afghan Refugee Villages of Pakistan. 2014.
39. WHO. Indicators for assessing infant and young child feeding practices. 2008.
40. Saraswati Khalsa, Emily Hejn, Sarriot E. Systems Effects Of Save The Children Emergency Health & Projects Sudan. 2019.
41. Holland R. Frontline: Support for breastfeeding in crisis. Lancet. 2017;390(10097):834.
42. Mukunya D, Tumwine JK, Nankabirwa V, Ndeezi G, Odongo I, Tumuhamye J, et al. Factors associated with delayed initiation of breastfeeding: a survey in Northern Uganda. Glob Health Action. 2017;10(1):1410975.
43. Program WIEPoB. Infant and Young Child Feeding in Emergency Situations. 1996.
44. Renata Jelušić R. Support for breastfeeding and young child feeding in emergencies. Experiences gained during the migrant and refugee crisis in Croatia in 2015 and 2016. 2017.
45. Group IC. Infant and Young Child Feeding in Emergencies Operational Guidance for Emergency Relief Staff and Programme Managers. 2017 October 2017.
46. Focus I. The Code and infant feeding in emergencies. 2009 May 2009.
47. Machel G. Impact of Armed Conflict on Children. United Nations; 1996 26 August 1996.
48. Association ILC. Breastfeeding: A Vital Emergency Response. Are You Ready? . 2009.
49. Association ILC. Breastfeeding: A Vital Emergency Response. Are You Ready? For relief workers. 2009.
50. WHO. International Code of Marketing of Breast-milk Substitutes. Geneva; 1981.
51. OXFAM. Feeding in Emergencies For Infants Under Six Months: Practical Guidelines 1996.
52. WHO, UNICEF, LINKAGES, IBFAN, ENN. Infant feeding in emergencies Module 1 (for emergency relief staff). 2001 November 2001.
53. WHO. WHA RESOLUTION 47.5- Infant and young child nutrition. 1994.
54. Joint UNICEF U, WFP, WHO Statement Policy on infant feeding in the Balkan Region.
55. UNHCR. UNHCR Policy Related To The Acceptance, Distribution And Use Of Milk Products In Refugee Settings 2006.
56. UNHCR. Guidance on Infant feeding and HIV in the context of refugees and displaced populations. 2008 April 2008.
57. UNHCR. Infant and young child feeding practices- Standard Operating Procedures for the Handling of Breastmilk Substitutes (BMS) in Refugee Situations for children 0-23 months. 2015 August 2015.
58. UNHCR, children St. Infant and Young Child Feeding in Refugee Situations: A Multi-Sectoral Framework for Action. 2018 May, 2018.
59. ACF-International. Baby friendly spaces. Holistic approach for Pregnant, Lactating Women anf their very young children in Emergency. 2014 December 2014.
60. ENN, IBFAN-GIFA, Fondation Terre des hommes, Action Contre la Faim, CARE USA, LINKAGES, et al. Infant feeding in emergencies (for health and nutrition workers in emergency situations). 2007.
61. Refugees UHCf. Interim Operational Considerations for the feeding support of Infants and Young Children under 2 years of age in refugee and migrant transit settings in Europe. 2015.
62. Save the Children U, Food Security and Nutrition Network. IYCF-E Toolkit: Summary of Operational Guidance on ICF-E Key Points and WHA Resolution. 2007.
63. Save the Children U, Food Security and Nutrition Network. ICFY-E Toolkit: Summary of Feeding the Non-breastfed child 6- 24 months of Age 2007.

For peer review only

64. Pediatrics AAo. Infant feeding in disasters and emergencies. 2015.
65. UNICEF. UNICEF Guidance on the provision and use of breastmilk substitutes in humanitarian settings. 2018 June 2018.
66. UNHCR. HOW TO GUIDE reproductive health in refugee setting (Strengthening Safe Motherhood Services). 1999 November 1998.
67. ENN. Infant Feeding in Emergencies: Policy, Strategy and Practice. 1999 May 1999.
68. McGrath M, Seal A, Taylor A, Gostelow L. Meeting the nutritional needs of infants during emergencies: Recent experiences and dilemmas: Report of an international workshop, Institute of Child Health, London, November 1999. 2000.
69. members UCG. Humanitarian Development Nexus Stakeholder and Literature Analysis. 2019.
70. Aguayo VM RJ, Kanon S, Ouedraogo AN. Aguayo VM, Ross JS, Kanon S, Ouedraogo AN (2003) Monitoring compliance with the International Code of Marketing of Breastmilk Substitutes in west Africa: multisite cross sectional survey in Togo and Burkina Faso. British Medical Journal. 326, 127. 2003.

Figures and Tables

Table 1: IYCF practices in conflict settings
Table 2: Programs/ Interventions to Promote Optimal IYCF Practices

Figure 1: Conceptual Framework
Figure 2: Search Flow Diagram
Figure 3: Barriers to optimal breastfeeding practices
Figure 4: Implementation Guidelines

Figure 1: Conceptual Framework

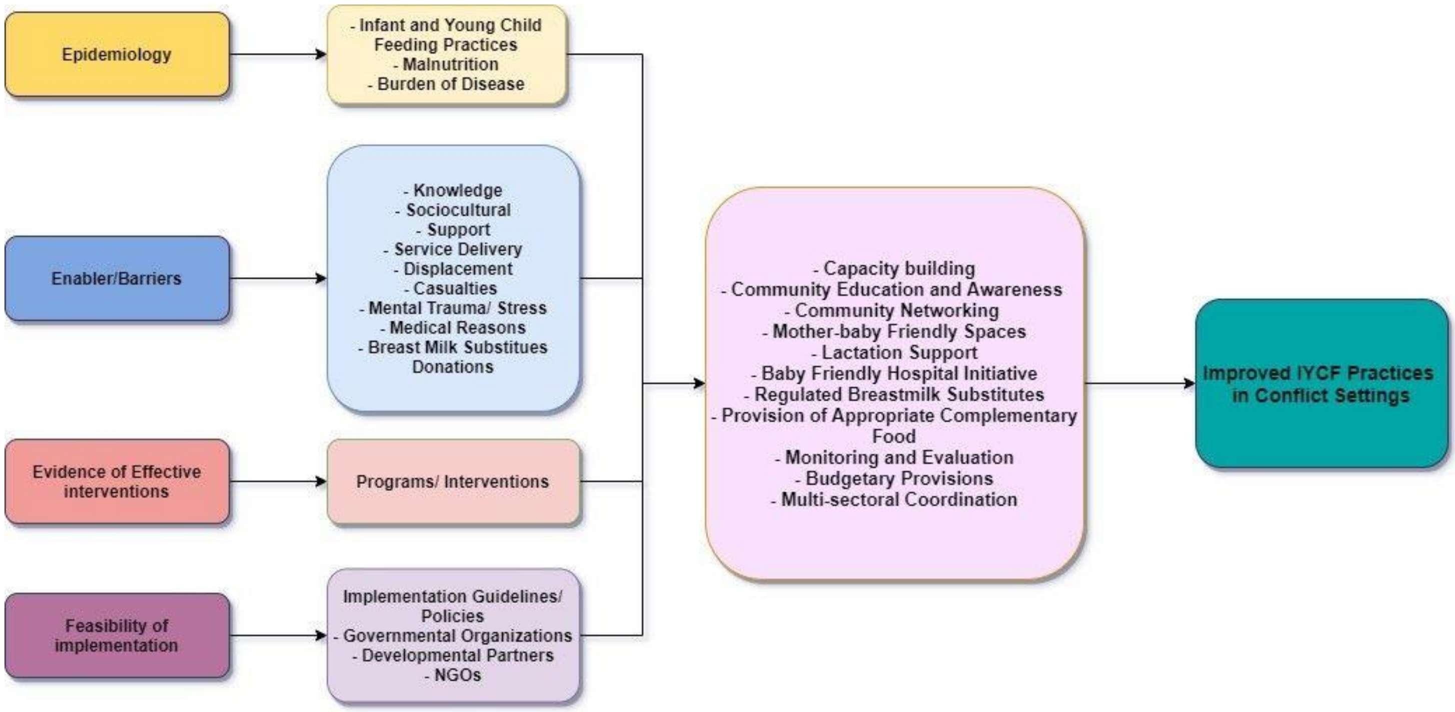

review only

Figure 2: Search Flow Diagram

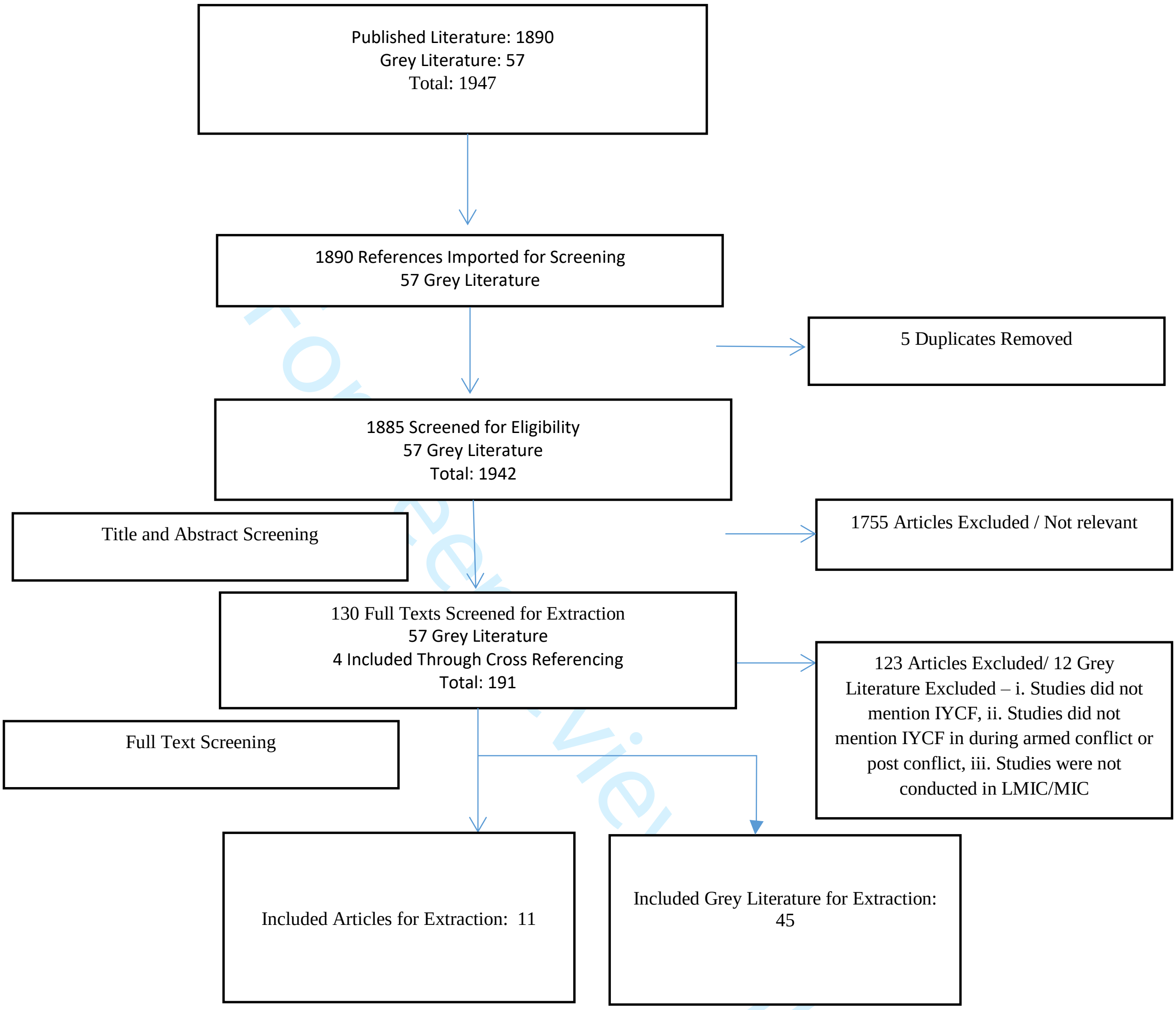

Figure 3: Barriers to optimal breastfeeding practices

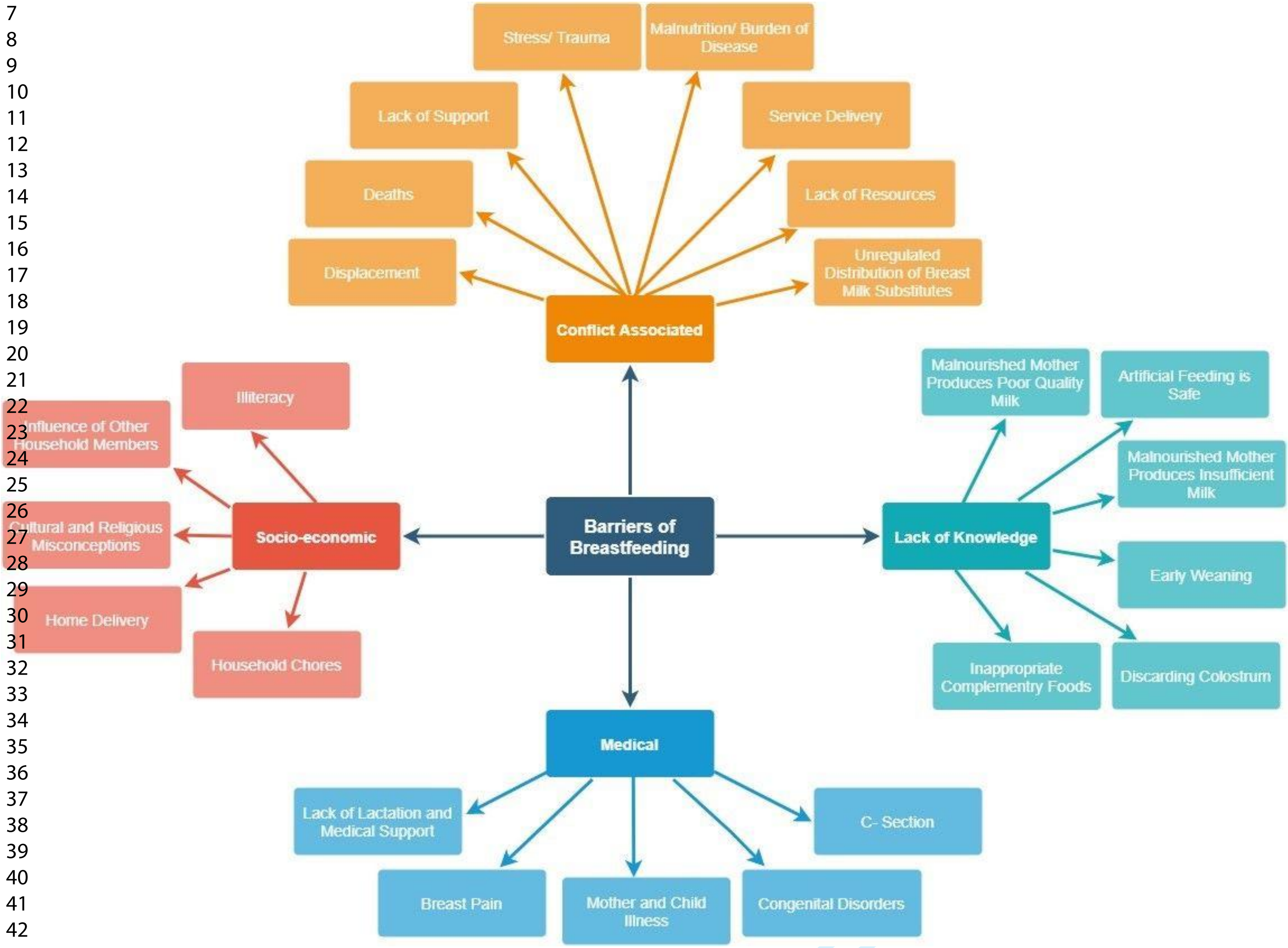

Figure 4: Implementation Guidelines

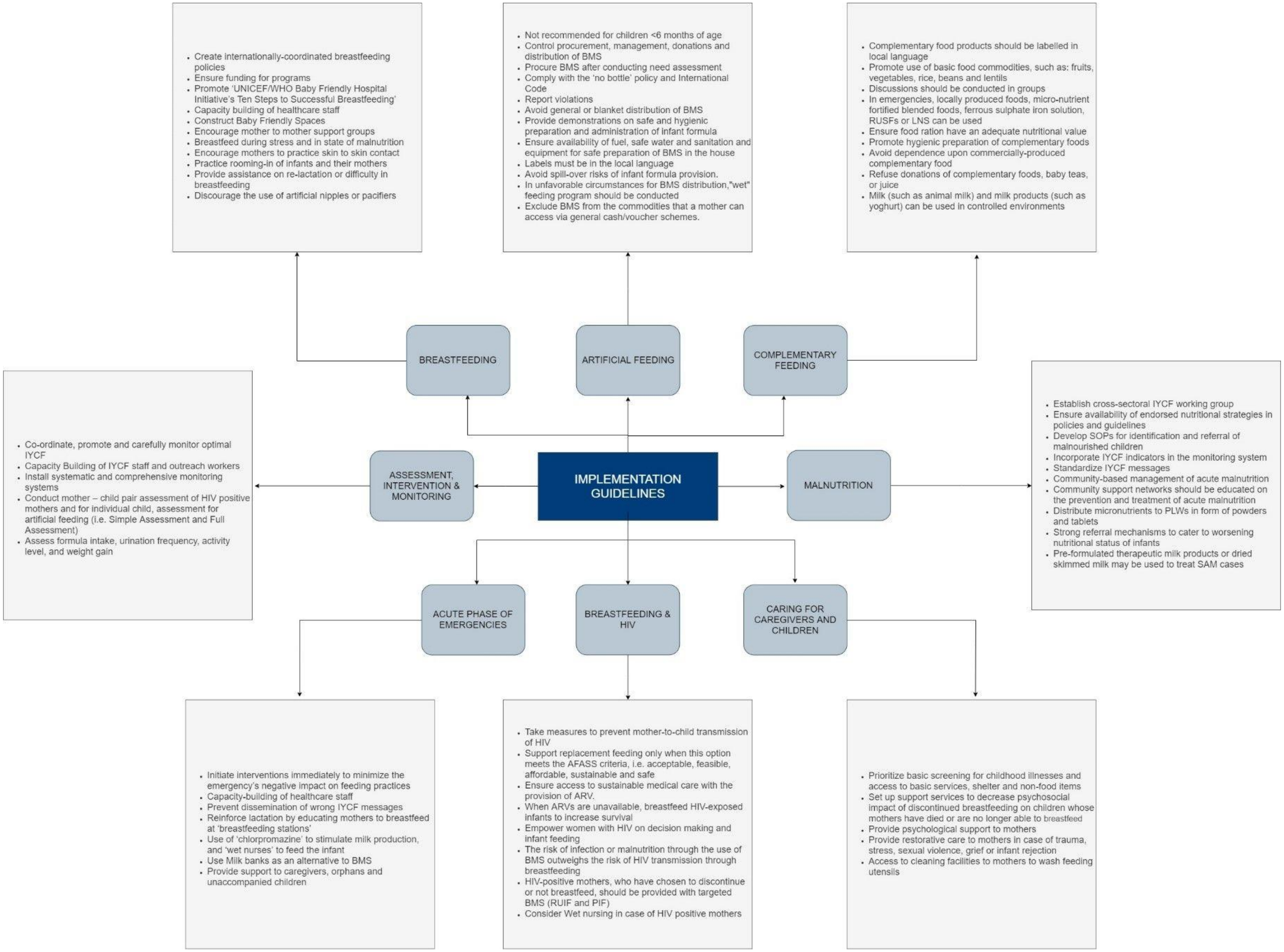

For peer review only - http://bmjopen.bmj.com/site/about/guidelines.xhtml

For peer review only

Appendix

Table 1: operational guidelines for IYCF in conflict

Components of breastfeeding	Operational Guidelines
**I.** Protecting, promoting and supporting breastfeeding	1. Infants should be exclusively breast fed for the first 6 months of life, followed by appropriate complementary feeding along with continued breastfeeding for two[1-14]<br>2. Relief agencies should create internationally-coordinated breastfeeding policies with practical guidelines explicitly stated for field workers and decision[2, 7, 10, 12, 13, 15, 16]<br>3. National policies and technical guidelines on IYCF- E should be explicitly stated and communicated to relevant staff in order to increase preparedness if an emergency strikes. This is particularly important for high-risk countries[2, 12, 13, 17, 18]<br>4. Funding should be provided for programs to support breastfeeding[2, 13, 19]<br>5. It should be ensured that optimal breastfeeding practices and maternal care is promoted according to 'UNICEF/WHO Baby Friendly Hospital Initiative's Ten Steps to Successful Breastfeeding'[2, 10, 20]<br>6. Ensure that relief workers, healthcare staff, technical and non-technical personnel are trained in appropriate infant and young child feeding practices using available training material and key information on IYCF integrated into routine assessment[2, 7, 10, 12-16, 20-23]<br>7. Appropriate and timely support and trainings for breastfeeding and young child feeding should be integrated at all levels of healthcare[2, 16, 21, 23]<br>8. Efforts should be made to raise awareness supporting the superiority of breastfeeding as a life-saving intervention to health personnel, relief staff, NGOs, stakeholders and the general public focusing primarily on pregnant and breastfeeding women[2, 10, 12, 20, 22, 24, 25]<br>9. In conflict and refugee settings, traditional birth attendants (TBAs) are more accessible to mothers than nurses and midwives, therefore TBAs should be trained regarding appropriate breastfeeding practices[17, 20, 24]<br>10. Pregnant women and breastfeeding mothers should be informed using clear language about maternal health, how to properly breastfeed, advantages and maintenance of breastfeeding, negative effects of bottle-feeding and the difficulty to reverse decision not to breastfeed[2, 6, 7, 10, 14, 17, 20, 22, 24, 25]<br>11. Encourage mothers to initiate breastfeeding within the first hour of birth, and to exclusively breastfeed for the first six months of life (do not give them extra water, juices, tea or food) unless medically indicated otherwise. Thereafter, solid foods should be introduced, but breastfeeding should continue for at least a year or two[2, 6, 7, 10, 11, 14, 15, 17, 26]<br>12. Educate mothers to not stop breastfeeding in emergency situations and spread awareness that exclusive breastfeeding provides the best nutrition to babies. Moreover, breastmilk contains ingredients that protects babies from infection, so it's particularly useful in emergency situations[2, 6, 10, 13, 15, 17]<br>13. Encourage mothers to breastfeed on demand[2, 7, 14, 17]<br>14. Practice rooming-in of infants and their mothers (allow mothers and infants to remain together 24 hours a day) to support breastfeeding practice [2, 7]<br>15. Colostrum should be given to the baby to improve its physical growth and feeding baby should not be interrupted in between. The baby lets go off the breast when he/she is done[10, 17, 26]<br>16. Discourage the use of artificial nipples or pacifiers[2, 7, 10, 24]

17. Educate mothers on breastfeeding and the procedure to maintain lactation even when the mother-infant pair is temporarily separated [2, 7, 10, 24]
18. Ensure that mothers are facilitated by the provision of counseling and other forms of assistance for the purpose of re-lactation or difficulty in breastfeeding[2, 6, 7, 10, 11, 14, 21, 23, 26, 27]
19. Mothers should also be made aware that breast milk supply is not reduced by stress, though the release of milk could be affected[6, 8, 10, 14, 18]
20. Encourage mothers to practice skin to skin contact as this aids in reducing stress (cortisol) levels and helps the flow of mother's milk. This can be practiced using slings and wraps. Moreover, correct positioning of baby during breastfeeding is important as effective suckling also triggers breast milk production[6, 10, 11, 14, 15, 17, 24, 26]
21. Malnourished mothers can produce enough milk to breastfeed, however, she should be treated for malnourishment[8, 10, 14, 15, 17, 26]
22. Relief workers should provide nutritional support by giving a general ration to pregnant and lactating women. If full general ration is not possible, then food and micronutrient supplements should be sufficiently provided. This is because an optimally fed mother will be able to optimally feed her infant. Give adequate food to the malnourished mother to prevent depletion of her own nutrients and closely monitor the weight and urine production of the infant[6, 10, 11, 15, 17, 18, 21, 23, 24, 26]
23. In emergency settings, extra breastfeeding support should be provided by encouraging Baby Friendly Spaces, which provide a platform to support mothers/caregivers and promote appropriate infant and young children feeding practices, privacy and safety[2, 11, 12, 15, 18, 21-24, 26]
24. For refugees and displaced populations, establish rest areas for pregnant women/caregivers/mothers and children in transit. These should be secluded, private and culturally appropriate areas that assist women and children to relax and nurse[2, 6, 11, 15, 18, 21, 23, 24]
25. Establish a program to encourage mother to mother/ women to women support[2, 6, 7, 20, 21, 23, 24]
26. Establish registration of newborn infants, i.e. within two weeks of delivery, to ensure timely additional rations and breastfeeding support for lactating and breastfeeding mothers. Nutrition workers should help mothers to establish exclusive breastfeeding[2, 6, 12, 16, 18, 23, 24]
27. Vulnerable groups, especially newly arriving mothers and infants with severe feeding problems should be identified and referred for immediate assistance[2, 6, 12, 15, 16, 18, 21, 23]
28. Create referral and follow-up systems for mothers/caregivers and their infants[2, 3, 6, 7]
29. Avoid giving estrogen containing contraceptive pills to mothers because they decrease breastmilk production[24]
30. Encourage mothers to build their confidence which leads to production of oxytocin to improve blood flow[26]
31. Continue to breastfeed sick children or when they are less hungry. In case they are not hungry then put them to breast more repeatedly to ensure that they take enough breastmilk[6, 10, 11, 14, 17, 26]
32. When natural breastfeeding is not possible, available alternatives should be evaluated before an appropriate choice is made. This may include evaluating between: wet-nursing, the use of milk bank, home-modified milk and the use of locally purchased commercial infant formula or generic unbranded infant formula[10, 12, 15, 23, 24, 28]
33. Access to infant formula should be based on the guidelines set by the WHO International Code of Marketing of Breast Milk Substitutes, 1981[10, 25]
34. Support should be provided for artificial feeding and this should be distinct from the support being provided for breastfeeding[1, 2, 4, 6, 10, 12, 19, 23]
35. Lactating women can take most medicines (including antibiotics) and can be immunized as well, as recommended for adults and adolescents to protect against infectious diseases (measles, mumps, rubella, tetanus, diphtheria, pertussis, influenza, Streptococcus pneumoniae, Neisseria meningitidis, hepatitis A, hepatitis B, varicella, and inactivated polio)[15]
36. In case of radiation exposure

For peer review only

	a. Women who were exposed to radiation should be advised to temporarily stop breastfeeding unless there is no other source of feeding available for the infant. These mothers should pump and discard their milk until the infant can resume breastfeeding<br>b. In case of interruption of breastfeeding, preferred source of infant feed is human milk that was pumped and stored prior to the radiation exposure or ready-to-feed infant formula. Mothers should use powdered or concentrated formula only if they are sure that water used to reconstitute is free from radiation<br>c. Mothers can resume breastfeeding when advised by local health officials or when they have been evacuated from the radiation affected area<br>d. Mothers do not need to stop breastfeeding if both mother and infant have been given appropriate doses of potassium iodide at the right time, according to the advice of local health officials[15]
**II.** Protecting non-breastfed infants and minimizing the risks of artificial feeding	1. Breast milk substitute (BMS) is not recommended for children <6 months of age[3, 4, 6, 7, 9, 10, 19, 21, 25, 28]<br>2. Procurement, management and distribution of breastmilk substitutes, bottles and teats, and commercial complementary foods should be controlled during emergencies and should comply with the 'no bottle' policy and International Code and WHA guidelines and violations should be reported[1-3, 10, 13, 16, 19, 21-23]<br>3. Procure BMS after conducting need assessment of artificial feeding at population level assessment. It may also include nutrition survey, household and community survey. This is recommended to be done in close collaboration with government bodies and by developing Program Cooperation Agreements (PCAs) with local bodies for task implementation[28]<br>4. On distribution of BMS, it should be ensured that workforce involved should have enough capacity for assessment, counselling, supply chain management and in providing support to families on WASH and IYCF practices[28]<br>5. Donations of free or subsidized breast-milk substitutes, bottles and teats and commercial baby foods at emergency sites should be refused and advocated against as this could put infants lives at risk and undermine breastfeeding practices[2-4, 6, 8, 11-13, 16, 21-23, 28]<br>6. The distribution, use and quantity of breast milk substitutes should be controlled by collecting unsolicited donations from all ports of entry and the recipient agencies, and should be stored and managed centrally by a single designated agency[1, 2, 4, 6, 10, 12, 13, 16, 19, 21, 23, 28]<br>7. BMS should only be distributed to the infants who really need it and have no viable breastmilk options, based on established criteria (where distribution can be targeted, the supply chain is secure, and the conditions for safe preparation and use can be met). This should be determined by a qualified health or nutrition worker trained in breastfeeding and infant feeding issues[1-4, 6, 7, 9-12, 14-16, 19, 21-23, 27]<br>8. Breast-milk substitutes, milk products, bottles and teats should never be part of a general or blanket distribution and their use should be avoided, especially in case of emergency situations or in situations where hygienic conditions can't be ensured and their use should be discouraged. Bottles should be exchanged for cups instead as it's easier to keep them clean[1-4, 6, 7, 10-12, 16, 18, 19, 21, 23, 24, 26-28]<br>9. Promotion of breast-milk substitutes at the point of distribution should be strictly discouraged. There should be no advertisement or display of products or items with milk company logos and BMS supplies should not be used as a sales inducement and there should be no provision of single tins/samples of BMS or gifts to mothers. Moreover, no incentives should be offered by manufacturers to health workers to promote BMS products[6, 9, 10, 21-23, 25, 28]<br>10. In case of crisis, WHO developmental partners and/or the designated nutrition co-ordinating agency should train as well as support training of staff and mothers on the proper and safe use of infant formula[2, 4, 10, 12, 16, 23]<br>11. When the use of infant formula is indicated, before its distribution, mothers and caregivers responsible for feeding should be educated on the specific care needed by a non-breast fed infant, and given practical training and one-

For peer review only

on one demonstrations by a skilled health worker on safe and hygienic preparation and administration of infant formula[1, 2, 4, 6, 7, 10, 12, 19, 21, 23]

12. There should be regular follow-up visits, to caregivers of targeted infants receiving BMS comprising of regular infant health and growth monitoring and distribution of BMS should be frequent and regular (at least bimonthly), with minimum delays[1, 3, 7, 10, 12, 19, 21]
13. It should also be ensured that there is availability of fuel, safe water and sanitation and equipment for safe preparation of BMS in the house, prior to distribution of BMS and implementing a household based program[2-4, 6, 7, 9-11, 14, 15, 21, 23, 25, 26, 28]
14. For infants determined to be in need of infant formula, the adequate and continued provision of infant formula must continue for as long as they require it, even if it needs to be purchased. IYCF-E programs that include an infant formula provision component should plan to provide it for at least 6 months, or if there is no availability of adequate complementary food, then 12 months[3, 4, 6, 7, 9-12, 14, 19, 22]
15. Non-breastfed infants over 6 months of age, provided with 6 months of BMS, may require 2-4 weeks of buffer supply to offer transition to non BMS feeding[3, 10]
16. An improvement should be brought about in health services to manage the adverse effects of artificial feeding, especially the increase in incidence of severe diarrhea and respiratory infections in infants[2, 10]
17. Generic, unbranded formula is recommended for infants who require infant formula, followed by locally purchased and relabeled infant formula (to be in compliance with The International Code). Home modified milk should only be used temporarily as the last resort for infants less than 6 months old. The type of infant formula should have a shelf life of at least 6 months and be appropriate for the infant, including their age[4, 6, 7, 9, 10, 15, 19, 21, 23]
18. Labels must adhere to the specific labelling requirements of The International Code and should be in the language of the local population. The BMS packaging should have clear instructions, with pictures, on how to use it, along with clearly specifying the superiority of breast milk[4, 6, 7, 9, 19, 21, 25, 28]
19. The labels should include a sign of “Important Notice” stating importance of breastfeeding and a statement stating, “The product should be used only on the advice of a health worker as to the need for its use and the proper method of use.” They instructions should also state about its safe preparation and health hazard on inappropriate use, and a warning against the health hazards on inappropriate preparation of infant formula[28]
20. Supply department should assure that BMS manufactured should follow Codex Alimentarius standards. UNICEF has not included BMS in it supply catalogue as a non-standard product[28]
21. For infants under 6 months of age, the only suitable BMS is infant formula. However, infants over the age of 6 months do not need infant formula but can use other sources of milk (pasteurized full-cream animal milk (cow, goat, sheep), Ultra High Temperature (UHT) milk, fermented milk or yogurt) as these are easier to find and are less dangerous than powdered milk. Condensed milk should not be used for infant feeding[4, 6, 10]
22. Liquid milk, if being used, should be consumed within a few hours of opening. Baby juices and teas should be avoided as they are low in nutrition and high in sugar. For infants over 6 months of age, infant formula can be mixed into the child’s food instead of giving it to drink[6]
23. Milk products should only be received and distributed in a dry form and even dried milk products should be distributed only when pre-mixed with a milled staple food and should not be distributed as a single commodity. However, dried skim milk is not an appropriate BMS for infant, and even for older children it must be fortified with vitamin A and not given on its own[4, 7, 9, 19, 23]
24. It should be ensured that there are no spill-over risks of infant formula provision. Measures should be taken to reduce spill-over by ensuring that feeding BMS to a minority of children doesn’t undermine breastfeeding practices of the majority. Advertising of infant formula should be stopped, and the provision of infant formula should be discrete, monetary support should be

For peer review only

	provided to breastfeeding mothers, and a separate space dedicated for breastfeeding support and counseling[1, 2, 4, 6, 10, 12, 19, 23]<br>25. When circumstances are not favorable for BMS distribution (safe preparation and use of infant formula can't be ensured), an on-site supplementary "wet" feeding program should be conducted in closed spaces under supervision[4, 7, 21, 23]<br>26. Infant formula should not be excluded from the commodities that a mother can access via general cash/voucher schemes. However, it should be accompanied by interventions such as providing essential information on breastfeeding and on how to reduce the risks of formula feeding[6]<br>27. Breast milk donations should be authorized and be regulated by the Ministry of Health. Until the arrival of the beneficiary, cold chain should be maintained for donated frozen breast milk[2, 26, 28]<br>28. The psychosocial impact of discontinued breastfeeding on children whose mothers have died or are no longer able to breastfeed should also be considered and support services should be set up to address this[12, 16]<br>29. One guideline mentioned that it should be known and taken care of that transitioning out from an emergency IYCF-E program with an infant formula provision component requires a solid, long-term exit strategy with ties to both facility and community-based structures[12]
**III.** Protecting, promoting and supporting appropriate, safe and timely complementary feeding	1. To promote growth and development of infants, encourage appropriate, timely and safe complementary feeding for infants (aged > 6 months) and young children (aged between 12 to < 24 months), along with continued breastfeeding[1-7, 9, 11, 12, 14, 18, 23]<br>2. Children over the age of 6 months of age should be given complementary foods (solid, semi-solid and soft foods) that are easy to eat and digest and nutritionally complement breast milk[1, 2, 6, 18]<br>3. Conduct discussion in groups on complementary feeding and weaning[26]<br>4. Promote the growth of locally produced food and encourage mothers/caregivers to use these locally-produced, inexpensive foods for complementary feeding. These include basic food commodities, such as: fruits, vegetables, rice, beans and lentils[9, 12, 18, 23]<br>5. Increase frequency and variety of complementary food with the growing age of child to meet his/her nutritional needs[26]<br>6. In emergencies, locally produced foods, micro-nutrient fortified blended foods, ferrous sulphate iron solution (iron drops), Ready-to-Use Supplementary Foods (RUSFs) or Lipid based Nutrient Supplements (LNS) can be used for complementary feeding depending on the nutritional situation[12, 18, 23, 28]<br>7. Special attention must be given to ensure that the food ration distributed for old infants and young children have an adequate nutritional value (food should be fortified with vitamins and minerals[4, 6, 16, 20, 23]<br>8. Establish services to extend nutritional support to vulnerable groups, such as: orphans and unaccompanied infants and young children[2, 6, 12, 15, 16, 21, 23]<br>9. Promote hygienic preparation of complementary foods by providing mothers/caregivers necessary information and support[6, 23]<br>10. In emergencies, special consideration should be made to mitigate the obstacles faced by mothers/caregivers in the preparation (cook, mash, etc.) of age-appropriate complementary foods. Measures needed to counter these obstacles should be incorporated in the program design[6, 12, 18]<br>11. IYCF-E programs relating to complementary feeding should include:<br>a. Group sensitization and education<br>b. Develop local food recipes focusing on nutritional value, affordability and food dietary diversity<br>c. Provide cooking demonstrations, and<br>d. Support mothers/caregivers to prepare complementary foods via initiatives such as fresh food vouchers[12]<br>12. Dependence upon commercially-produced complementary foods should be avoided[9, 23]<br>13. Mothers should be assured that in the absence of safe complementary foods, breastmilk is a significant source of nutrition for infants the first year of life and beyond[15]

For peer review only

		14. Donations of complementary foods, baby teas, or juices should be refused. If there are donated supplies, they should be directed to the designated coordinating agency on nutrition/health to be managed appropriately[6]<br>15. Promotion of a varied diet, dairy products in particular, to ensure that energy, protein, mineral and vitamin requirements are met. Moreover, when preparing food for infants such as porridges, mashed potatoes, etc., milk and infant formula can be added to it to increase the nutrient content[4, 6, 20, 26]<br>16. Pastoral communities mainly use milk and milk products, which contains a significant amount of nutrition for children over six months. As a part of complementary feeding, milk (such as animal milk) and milk products (such as yoghurt) can be provided to infants over 6 months of age. In such cases, distribution of milk products can only be conducted in controlled environments under strict supervision, such as on-the-spot feeding[3]<br>17. Good nutrient sources for infants are animal source foods, such as yogurt and cheese whereas products containing only fruits/vegetables are less energy and nutrient dense. Baby teas and juices should not be given to infants as they do not have much nutrient value[6]<br>18. Complementary food products should be labelled in the language of the targeted population and be acceptable to them, according to their culture. The products should also have information on how to prepare the food[6]<br>19. Moreover, these products should not have images of bottle feeding on them or recommend it in any way[6]<br>20. If needed, commercial 'baby' foods should be purchased and distributed to mothers or products recommended to mothers, for infants over 6 months of age, that have the most nutritional value[6]<br>21. It is recommended that children over the age of 12 months eat the same foods as older children[6]
**IV.**	Caring for care-givers	1. Efforts should be made to increase caregivers' coping capacity because the number of caregivers is often reduced during emergencies and stress levels increase, so psychological support and encouragement plays an important role in enhancing optimal IYCF-E practices[1, 2, 15, 17, 18]<br>2. Treat the mother during illness, keep the infant close to the mother and do not stop breastfeeding. The mother should be educated on the appropriate method of milk expression as she can maintain breastmilk flow through milk expression[17, 24]<br>3. Breastfeeding mothers require identification, protection and active support. Conflict situations and displacement can negatively affect maternal confidence and breastfeeding practices. Breastfeeding mothers tend to stop or reduce breastfeeding in such situations and thus a lot of support and counselling should be provided to newly arriving refugee mothers, caregivers and infants with special needs (orphans and unaccompanied children)[2, 5, 6, 12, 17, 18, 21, 23]<br>4. Provide restorative care to mothers in case of trauma, stress, sexual violence, grief or infant rejection[24]<br>5. Adequately trained and skilled staff should support mothers with difficulties in breastfeeding their infants and provide private safe spaces for mothers to breastfeed and to connect with other mothers. Access to cleaning facilities should also be provided to mothers to wash feeding utensils, especially to mothers who are formula feeding. Prioritize mothers of infants and young children for basic screening of childhood illnesses, access to registration and basic services, shelter and non-food items[2, 6, 10, 15, 18]<br>6. It should be ensured that mothers/caregivers of artificially fed infants are given targeted support and receive counselling as well as kits for preparing BMS safely, including soap, fuel, water purification tablets[2, 18]
**V.**	Protecting Children	1. Artificially-fed infants need more protection and support than breast-fed children[6, 10]<br>2. Infants are at higher risk of malnutrition and illness like diarrhea and chest infections if they are being fed infant formula. In emergency conditions, this could be fatal[2, 6, 10, 11]<br>3. In case the infant is ill (e.g. if the infant has diarrhea), continue feeding the infant since breastmilk contains water to replace losses through diarrhea and important minerals and vitamins to help prevent dehydration. It also contains

For peer review only

	proteins to help strengthen the immune system of the baby. Give smaller amounts of breastfeed and more frequently if the infant's appetite is reduced. However, in serious or prolonged cases of diarrhea, rehydration therapy may be required[6, 10, 11, 15, 26]<br>4. Newborn infants are the most vulnerable group which should be targeted and ensured that they are exclusively breast fed and artificial feeding is prevented[2, 6, 12, 16, 21, 23]<br>5. Support breastfeeding as the best way to safeguard infants against cholera. Infants with cholera should continue breastfeeding as soon as they are able to suckle, and mothers with cholera should re-initiate breastfeeding as soon as they are stable[12]<br>6. Efforts should be made to protect cholera-free breast-fed babies from cross-contamination[12]<br>7. IYCF managers, field staff and registration staff should collaborate and ensure screening of infants at registration to identify and refer infants at risk or those who are highly vulnerable (i.e. orphans and unaccompanied infants and young children, non-breastfed infants <6 months, ill infants or malnourished infants) and establish services to give these vulnerable infants nutritional support[2, 6, 12, 16, 18, 21, 23]<br>8. Prioritize support service:<br>a. For re lactation: Prioritize non-breastfed infants 0 - <6 months or of 0 - <2 months whose mother/wet nurse is willing to re lactate<br>b. Re-establish exclusive breastfeeding: Always prioritize infants aged 0 - <2 months (including newborns). Infants 0 - <4 months are a priority, but it's an ideal situation for infants 0 - <6 months<br>c. BMS provision and associated support services: prioritize not breastfed infants who are <6 months[3]<br>9. The psychosocial impact of discontinued breastfeeding on children whose mothers have died or are no longer able to breastfeed should also be considered and support services should be set up to address this[12, 16]<br>10. Consideration should also be given to special needs of artificially fed children (0-23 months) and PLW (i.e. insecticide treated mosquito nets; hygiene items including soap and washing containers; cooking and eating items; breastfeeding shawls; blankets and infant/young child clothing and shoes with thermal needs considered; potties, diapers), with a particular preference for items that can be locally sourced[2]
**VI.** Malnutrition	1. Ensure availability of endorsed nutritional strategies in policies and guidelines and establish cross-sectoral IYCG working group to discuss challenges, needs and success[2]<br>2. IYCF teams should coordinate and generate a treatment plan where mothers should be admitted at stabilization centers along with her children where they would be provided with nutritional support and counselling to improve lactation[2]<br>3. Select an IYCF specialist from each nutrition post or community group[2]<br>4. Standardize IYCF messages for caregivers and PLWs with children of 0-23 months and incorporate IYCF indicators in the monitoring system[2]<br>5. Develop standard procedure for health workers for identification and referral of malnourished children[2]<br>6. Promote IYCF practices (i.e. early initiation, exclusive breastfeeding and complementary feeding)[2]<br>7. Timely introduction of complementary food in infants of 6-9 months[2]<br>8. Micronutrients should be distributed to all PLWs in form of powders and tablets[2]<br>9. Malnutrition treatment and prevention programs should incorporate and prioritize infant and young child feeding in their agenda[2, 12]<br>10. Efforts should be made to investigate the underlying cause of malnutrition and measures should be taken to correct it[1, 2, 10]<br>11. Monitor nutritional status of mothers, infants and young children with the purpose of identifying, assessing, preventing and treating malnourished children[1, 2, 10]<br>12. Strong referral mechanisms should be put in place for acute malnutrition to cater to worsening nutritional status of infants[2, 12]

For peer review only

	13. Admit malnourished children along with their mothers to a nutritional rehabilitation program[12, 21, 24]<br>14. There should be community-based management of acute malnutrition, if conditions are favorable. In disasters, supplementary feeding should be the primary strategy for prevention and treatment of moderate acute malnutrition (MAM). Depending on vulnerable population groups and malnutrition level/risk of an increase in acute malnutrition, supplementary feeding can be blanket or targeted[2]<br>15. Pre-formulated therapeutic milk products or dried skimmed milk (DSM) may be used to treat cases of severe acute malnutrition (SAM). However, attention must be given to ensure that supplementary food aid commodities are carefully regulated and distributed to only target vulnerable groups[4, 9]<br>16. Therapeutic milks are not appropriate for BMS use. Therapeutic milk like F75 and F100 should be used for the treatment of children with severe acute malnutrition (SAM). It can be given to infants less than 6 months. SAM children require appropriate treatment at right time with immediate referral[28]<br>17. Cash/voucher programmes (conditional) could be started that promote good nutrition outcomes i.e. preventing malnutrition. Unconditional cash programs should be promoted in case of food security and livelihood. But in this case optimal IYCF practices should be considered through an expert. Women should be educated and sensitized for appropriate use on food and nonfood items for children of 0-23 months[2]<br>18. Community support networks should be educated on the prevention and treatment of acute malnutrition[2]
**VII.** The acute phase of emergencies (prevention through interventions)	1. In case of an emergency, interventions should start immediately to minimize the emergency's negative impact on feeding practices and every agency should develop a policy on infant feeding in emergencies, focusing on supporting caregivers and nutritional needs children[1, 2, 4, 23]<br>2. An appropriate agency should be appointed and resourced at the start of an emergency to co-ordinate IYCF-E practices and ensure the implementation of policies and it should be conveyed to all agencies working in the area[13]<br>3. In emergencies, donations of BMS are not needed and may put endanger infant lives due to poor hygienic conditions[2, 4, 10, 12, 13, 23]<br>4. If emergencies occur in places where there was already high infant formula use, promotion of IYCF-E can be even more difficult and WHO, and its developmental partners, along with local authorities and/or the national Nutrition Cluster (if activated) should ensure that appropriate IYCF-E is adequately promoted, protected and supported[2, 10, 12, 23]<br>5. Interventions should be undertaken to increase the prevalence of appropriate IYCF-E practices such as culturally-appropriate behavior -change approaches, along with capacity-building, to increase the rate of exclusive breastfeeding[12, 13, 23]<br>6. A joint statement for protection and support of appropriate IYCF-E should be released and ensured that BMS donations and distributions are carefully monitored[2, 4, 7, 12, 13, 23]<br>7. WHO, its developmental partners, local governments and national Nutrition Cluster (if activated) should work on this and provide this information to all staff, potential donors (including governments and the military) and the media, and ensure that no wrong messages are being disseminated both in emergency preparedness and particularly during the early phase of an emergency response[2, 4, 12, 13, 23]<br>8. Breastfeeding and IYCF support should be a major component of all services for mothers, infants and children and measures should be put in place to ensure that their needs are met in the early stages of an emergency[2, 4, 7, 16]<br>9. Support should also be provided to caregivers and infants with special needs (orphans and unaccompanied children)[2, 6, 12, 14-16, 21, 23]<br>10. It should be ensured that artificial feeding is strictly restricted to the targeted group of infants that require it and mothers who need help with breastfeeding are provided lactation support by mobilizing 'local breastfeeding facilitators'[7, 10]<br>11. Lactation should be reinforced by educating mothers to breastfeed every 2-3 hours at 'breastfeeding stations' scattered across refugee sites[7]

For peer review only

		12. One guideline also suggested the use of 'chlorpromazine' to stimulate milk production, according to the protocol and also 'wet nurses' to feed the infant[7]<br>13. Milk banks can also be used as an alternative to BMS and also as a source of employment in emergency setting[7, 10]
**VIII.**	Assessment, intervention and monitoring	1. In emergencies, co-ordinate, promote and carefully monitor optimal feeding in infants and young children[1, 2, 15, 23]<br>2. There should be systematic and comprehensive monitoring systems to track all infant feeding products being distributed[13]<br>3. Conduct mother – child pair assessment of HIV positive mothers and for individual child, assessment for artificial feeding (i.e. Simple Assessment and Full Assessment)[3, 24]<br>4. In areas with high prevalence of HIV, the risk of infant getting HIV via breastfeeding should be weighed against the risk of facing infection and malnutrition as a consequence of not being breastfed[1]<br>5. The prevalence of HIV in the affected population, knowledge of HIV status, and availability of counselling and testing facilities should be assessed (including pre-emergency estimates) using secondary sources and relevant information from health information systems[10]<br>6. In emergencies, implement an IYCF-E program focusing on infant feeding provision, with robust mechanisms that estimate the number of children that don't have access to breast milk and then provide them with targeted supply of infant formula[12]<br>7. Monitor the nutritional status of infants and young children, particularly weight monitoring for those receiving formula feed and assess intake, urination frequency, activity level, whether infant is feeding vigorously and weight gain[6, 10, 12, 15, 24, 26]<br>8. Establish a strong referral system to treat acute malnutrition should the infant's nutritional status deteriorate[2, 12]<br>9. To monitor and to conduct rapid assessments, gather information and statistics regarding: demographic profile, morbidity, mortality, predominant feeding practice, reported feeding problems for infants and young children including problems related to breastfeeding and complementary feeding, pre-crises approach to orphaned children, security risks and availability of conspicuous BMS products and bottles/ teats/ breast pumps[7, 10, 21, 23]<br>10. To monitor and assess, use qualitative methods to gather data regarding:<br>a. Appropriate complementary foods in the general ration or targeted feeding programs<br>b. Maternal and child health facilities including antenatal, delivery, postnatal and child care<br>c. Capacity of potential support-givers including breastfeeding mothers, trained health workers, trained counselors and experienced women from the community<br>d. Factors that may disturb breastfeeding practices<br>e. Key decision-makers at household, community and local health facility level that may influence infant and young child feeding practices<br>f. Cultural barriers affected practices of re-lactation, wet-nursing, etc.<br>g. General health environment including: water and sanitation, housing, facilities of food preparation and cooking[10, 21, 23, 28]<br>11. To monitor and assess, use quantitative methods to gather data regarding:<br>a. Estimated number of unaccompanied and accompanied children under two years of age, pregnant and lactating women<br>b. Statistics regarding morbidity, mortality, and levels of malnutrition<br>c. Information concerning nutritional adequacy of food rations<br>d. Pre-crisis and recent patterns in infant and young child feeding practices<br>e. Availability and management of BMS in accordance to The International Code[2, 10, 21, 23]<br>12. Governments should monitor and apply The International Code collaboratively with the assistance of International agencies such as WHO and UNICEF, NGO's, refugee camp staff, professional groups and customer organizations to ensure that manufacturers and distributors of BMS remain within the scope of the established Code[2, 10, 25]

For peer review only

	13. Manufacturers and distributors should monitor their market prices and the practices of their marketing personnel in accordance to The Code. Non-governmental organizations (NGO's) along with professional groups, institutions and concerned individuals should monitor and criticize manufacturers and distributors that don't follow the principles of The Code[10, 25]<br>14. Review and monitor the following:<br>a. Advice and knowledge regarding breastfeeding and BMS usage<br>b. Estimate the number of women breastfeeding, weaning and incorporating the use of BMS and bottle in the feed of their infant and young children<br>c. Constraints associated with hygienic BMS preparation<br>d. Availability and management of BMS[4, 7, 10, 20, 21, 23, 25]<br>15. Promote importance of breastfeeding and optimal hygiene practices especially handwashing before preparation of BMS. We should also dispel myths among mothers regarding breastfeeding[28]<br>16. Capacity Building of IYCF staff and outreach workers on nutrition (optimal IYCF practices, lifesaving IYCF practices, rapid IYCF assessment and detection of poor IYCF practices)[2]
**IX.** Breastfeeding, HIV, and other considerations	1. Generate policies on empowering women with HIV on decision making and infant feeding[8]<br>2. IYCF staff should take appropriate measures to prevent mother-to-child transmission of HIV, as well as focus on improving child survival from HIV[2]<br>3. Promote the use of optimal infant and young child feeding guidelines when the HIV status of the mother is unknown or she is HIV negative[2-4, 10, 23, 24, 29]<br>4. In case of unavailability of HIV-testing, it is recommended to breastfeed the infant for six months, followed by adequate complementary feeding and continued breast feeding for two years[24, 29]<br>5. Mothers should know their HIV status and receive appropriate counselling. Those diagnosed as HIV positive should make an informed decision about feeding options by balancing the prevention of HIV transmission with the nutritional requirements of infants[1, 2, 4, 8, 10, 12, 23, 24, 29]<br>6. HIV positive mother should exclusively breastfeed her child for first 6 months of life unless replacement feeding is affordable, sustainable and safe for their infants. In case, replacement feeding is not acceptable, then complementary feeding with continued breastfeeding at 6 months is recommended, while mother and baby will be assessed regularly[2, 10, 24]<br>7. Supportive arrangements and personal attachment for HIV positive mothers helps to reduce isolation, build confidence, reduce conflicting messages, encourage age appropriate feeding, provide privacy and educate family members[24]<br>8. Ensure access to sustainable medical care for mothers with an HIV positive status by supporting the provision of ART and ARV. If due to emergencies, the supply of these medications is hindered then immediate action should be taken for its re-establishment[12, 23, 24, 29]<br>9. In circumstances during acute emergencies, when Antiretroviral Drug (ARVs) are unavailable, it is recommended to breastfeed HIV-exposed infants to increase his/her survival[2, 3]<br>10. In emergency contexts, HIV-positive mothers should be supported to initiate or continue exclusive breastfeeding/ continued breastfeeding with adequate complementary feeding depending upon the age of the infant. The risk of infection or malnutrition through the use of Breast Milk Substitutes (BMS) outweighs the risk of HIV transmission through breastfeeding[2, 8, 12, 21, 23, 24]<br>11. Support replacement feeding *only* when this option meets the AFASS criteria, i.e. acceptable, feasible, affordable, sustainable and safe[3-5, 23, 24, 29]<br>12. All HIV- positive mothers should receive full support and get regular follow ups[2, 29]<br>13. HIV-positive and caregivers of children born to HIV-positive mothers, who have chosen to discontinue or not breastfeed, should be provided with targeted, appropriate, breast milk substitutes (Ready to Use Formula (RUIF) and Powdered Infant Formula (PIF)[2, 12]<br>14. Promote and support specific counseling concerned with risks of mixed feeding and HIV transmission. Additionally, ensure the provision of Safe BMS kits, i.e. adapted to the type of BMS administered[2, 12]

For peer review only

	15. Services and activities linked to the Prevention/Elimination of Mother-to-Child Transmission (E/PMTCT) should be provided routinely as a part of nutritional interventions[2, 12]<br>16. Measures taken by ICYF-E staff should be sensitive and should avoid actions that may exacerbate any HIV-related stigma[2, 12]<br>17. Wet nursing should also be considered in case of HIV positive mothers and for infants who have lost their mothers. It should be administered by any person (other than mother). The wet nurse should be counselled before and after wet nursing to prevent her from catching infection[10, 12, 24, 29]<br>18. WHO recommends flash heated breastmilk rather than boiling breast milk to prevent significant nutritional damage of breastmilk[29]

References:

1. WHO. Guiding principles for feeding infants and young children during emergencies. 2004.
2. UNHCR, children St. Infant and Young Child Feeding in Refugee Situations:

A Multi-Sectoral Framework for Action. 2018 May, 2018.
3. UNHCR. Infant and young child feeding practices

Standard Operating Procedures for the Handling of Breastmilk Substitutes (BMS) in Refugee Situations for children 0-23 months. 2015 August 2015.
4. UNHCR. UNHCR Policy Related To The Acceptance, Distribution And Use Of Milk Products In Refugee Settings 2006.
5. Save the Children U, Food Security and Nutrition Network. ICFY-E Toolkit: Summary of Feeding the Non-breastfed child 6- 24 months of Age 2007.
6. Refugees UHCf. Interim Operational Considerations for the feeding support of Infants and Young Children under 2 years of age in refugee and migrant transit settings in Europe. 2015.
7. OXFAM. Feeding in Emergencies For Infants Under Six Months: Practical Guidelines 1996.
8. Machel G. Impact of Armed Conflict on Children. United Nations; 1996 26 August 1996.
9. Joint UNICEF U, WFP, WHO Statement Policy on infant feeding in the Balkan Region.
10. ENN. Infant Feeding in Emergencies: Policy, Strategy and Practice. 1999 May 1999.
11. Association ILC. Breastfeeding: A Vital Emergency Response. Are You Ready? . 2009. p. 2.
12. ACF-International. Infant and Young Child Feeding in Emergencies. 2015 December 2015.
13. Borrel A, Taylor A, McGrath M, Seal A, Hormann E, Phelps L, et al. From policy to practice: challenges in infant feeding in emergencies during the Balkan crisis. Disasters. 2001;25(2):149-63.
14. Program WIEPoB. Infant and Young Child Feeding in Emergency Situations. 2005.
15. Pediatrics AAo. Infant feeding in disasters and emergencies. 2015.
16. Save the Children U, Food Security and Nutrition Network. IYCF-E Toolkit: Summary of Operational Guidance on ICF-E Key Points and WHA Resolution. 2007.
17. UNHCR. HOW TO GUIDE reproductive health in refugee setting (Strengthening Safe Motherhood Services). 1999 November 1998.
18. Children St. Assessment of infant and young child feeding practices among refugees on Lesvos Island, Greece. 2016.
19. Focus I. The Code and infant feeding in emergencies. 2009 May 2009.
20. USAID TSG, International Medical Corps Camp-based Barrier Analysis of Early Initiation of Breastfeeding, Iron-rich Food Consumption, and Early Antenatal Care Seeking Behaviors of Syrian Refugees in Azraq Camp, Jordan. 2016.

For peer review only

21. WHO, UNICEF, LINKAGES, IBFAN, ENN. Infant feeding in emergencies Module 1 (for emergency relief staff). 2001 November 2001.
22. WHO. WHA RESOLUTION 47.5

Infant and young child nutrition. 1994.
23. Group IC. Infant and Young Child Feeding in Emergencies Operational Guidance for Emergency Relief Staff and Programme Managers

2017 October 2017.
24. ENN, IBFAN-GIFA, Fondation Terre des hommes, Action Contre la Faim, CARE USA, LINKAGES, et al. Infant feeding in emergencies Module 2 Version 1.1 (for health and nutrition workers in emergency situations). 2007.
25. WHO. International Code of Marketing of Breast-milk Substitutes. Geneva; 1981.
26. ACF-International. Baby friendly spaces

Holistic approach for Pregnant, Lactating Women anf their very young children in Emergency. 2014 December 2014.
27. Action Against Hunger U. Infant Feeding and Weaning Report 2000.
28. UNICEF. UNICEF Guidance on the provision and use of breastmilk substitutes in humanitarian settings. 2018 June 2018.
29. UNHCR. Guidance on Infant feeding and HIV in the context of refugees and displaced populations. 2008 April 2008.

For peer review only

PRISMA 2009 Checklist

Section/topic	#	Checklist item	Reported on page #
**TITLE**			
Title	1	Identify the report as a systematic review, meta-analysis, or both.	1
**ABSTRACT**			
Structured summary	2	Provide a structured summary including, as applicable: background; objectives; data sources; study eligibility criteria, participants, and interventions; study appraisal and synthesis methods; results; limitations; conclusions and implications of key findings; systematic review registration number.	2
**INTRODUCTION**			
Rationale	3	Describe the rationale for the review in the context of what is already known.	4,5
Objectives	4	Provide an explicit statement of questions being addressed with reference to participants, interventions, comparisons, outcomes, and study design (PICOS).	4,5
**METHODS**			
Protocol and registration	5	Indicate if a review protocol exists, if and where it can be accessed (e.g., Web address), and, if available, provide registration information including registration number.	
Eligibility criteria	6	Specify study characteristics (e.g., PICOS, length of follow-up) and report characteristics (e.g., years considered, language, publication status) used as criteria for eligibility, giving rationale.	5
Information sources	7	Describe all information sources (e.g., databases with dates of coverage, contact with study authors to identify additional studies) in the search and date last searched.	6
Search	8	Present full electronic search strategy for at least one database, including any limits used, such that it could be repeated.	6
Study selection	9	State the process for selecting studies (i.e., screening, eligibility, included in systematic review, and, if applicable, included in the meta-analysis).	6
Data collection process	10	Describe method of data extraction from reports (e.g., piloted forms, independently, in duplicate) and any processes for obtaining and confirming data from investigators.	6
Data items	11	List and define all variables for which data were sought (e.g., PICOS, funding sources) and any assumptions and simplifications made.	6
Risk of bias in individual studies	12	Describe methods used for assessing risk of bias of individual studies (including specification of whether this was done at the study or outcome level), and how this information is to be used in any data synthesis.	NA
Summary measures	13	State the principal summary measures (e.g., risk ratio, difference in means).	NA
Synthesis of results	14	Describe the methods of handling data and combining results of studies, if done, including measures of consistency (e.g., I^2) for each meta-analysis.	NA

For peer review only

PRISMA 2009 Checklist

Section/topic	#	Checklist item	Reported on page #
Risk of bias across studies	15	Specify any assessment of risk of bias that may affect the cumulative evidence (e.g., publication bias, selective reporting within studies).	NA
Additional analyses	16	Describe methods of additional analyses (e.g., sensitivity or subgroup analyses, meta-regression), if done, indicating which were pre-specified.	NA
**RESULTS**			
Study selection	17	Give numbers of studies screened, assessed for eligibility, and included in the review, with reasons for exclusions at each stage, ideally with a flow diagram.	7
Study characteristics	18	For each study, present characteristics for which data were extracted (e.g., study size, PICOS, follow-up period) and provide the citations.	7
Risk of bias within studies	19	Present data on risk of bias of each study and, if available, any outcome level assessment (see item 12).	NA
Results of individual studies	20	For all outcomes considered (benefits or harms), present, for each study: (a) simple summary data for each intervention group (b) effect estimates and confidence intervals, ideally with a forest plot.	NA
Synthesis of results	21	Present results of each meta-analysis done, including confidence intervals and measures of consistency.	NA
Risk of bias across studies	22	Present results of any assessment of risk of bias across studies (see Item 15).	N/A
Additional analysis	23	Give results of additional analyses, if done (e.g., sensitivity or subgroup analyses, meta-regression [see Item 16]).	NA
**DISCUSSION**			
Summary of evidence	24	Summarize the main findings including the strength of evidence for each main outcome; consider their relevance to key groups (e.g., healthcare providers, users, and policy makers).	16
Limitations	25	Discuss limitations at study and outcome level (e.g., risk of bias), and at review-level (e.g., incomplete retrieval of identified research, reporting bias).	18
Conclusions	26	Provide a general interpretation of the results in the context of other evidence, and implications for future research.	18
**FUNDING**			
Funding	27	Describe sources of funding for the systematic review and other support (e.g., supply of data); role of funders for the systematic review.	21

From: Moher D, Liberati A, Tetzlaff J, Altman DG, The PRISMA Group (2009). Preferred Reporting Items for Systematic Reviews and Meta-Analyses: The PRISMA Statement. PLoS Med 6(7): e1000097. doi:10.1371/journal.pmed1000097

For more information, visit: **www.prisma-statement.org**.

For peer review only - http://bmjopen.bmj.com/site/about/guidelines.xhtml
